# Design, Synthesis, Antimicrobial Properties, and Molecular Docking of Novel Furan-Derived Chalcones and Their 3,5-Diaryl-∆^2^-pyrazoline Derivatives

**DOI:** 10.3390/antibiotics13010021

**Published:** 2023-12-24

**Authors:** Inas S. Mahdi, Ahmed Mutanabbi Abdula, Abdulkadir M. Noori Jassim, Younis Baqi

**Affiliations:** 1 Department of Chemistry, College of Science, Mustansiriyah University, Baghdad P.O. Box 14022, Iraq; inas.salim@coagri.uobaghdad.edu.iq (I.S.M.); kadirchem@uomustansiriyah.edu.iq (A.M.N.J.); 2 Department of Chemistry, College of Science, Sultan Qaboos University, Muscat P.O. Box 36, Oman

**Keywords:** antimicrobial, docking, chalcone, pyrazoline, synthesis

## Abstract

The present work focuses on the synthesis and preliminary structure activity relationships (SARs) of furan-derived chalcones and their corresponding ∆^2^-pyrazoline derivatives as antimicrobial agents. Eight novel chalcone derivatives and eight ∆^2^-pyrazoline compounds were synthesized in moderate to good isolated yields. The target compounds were evaluated as antimicrobial agents against two Gram-positive (*Staphylococcus aureus* and *Staphylococcus epidermidis*), two Gram-negative (*Escherichia coli* and *Klebsiella pneumoniae*), and fungi (*Candida albicans*) species. Based on the SARs, chalcones **2a** and **2h** showed inhibition activity on all tested microbial species, while ∆^2^-pyrazoline **3d** was found to be selective for some microbial species. The most potent compounds (**2a**, **2h,** and **3d**) were docked into glucosamine-6-phosphate synthase (GlcN-6-P), the molecular target enzyme for antimicrobial agents, utilizing the Autodock 4.2 program, in order to study their virtual affinity and binding mode with the target enzyme. The selected potent compounds were found to bind to the active site of the enzyme probably in a similar way to that of the substrate as suggested by the docking study. In summary, the newly developed furan-derived chalcones and their ∆^2^-pyrazoline derivatives could serve as potent leads toward the development of novel antimicrobial agents.

## 1. Introduction

Antimicrobial resistance (AMR) is a chronic public health problem, estimated to be the cause of 10 million deaths per year globally by 2050 [1]. According to the World Health Organization (WHO), AMR is one of the top 10 global public health threats facing humanity [2,3,4]. AMR is often a result of the misuse and overuse of existing antimicrobial drugs, which is the main driver in the development of drug-resistant pathogens [5,6,7]. Therefore, it is urgently needed to develop new antimicrobial drugs bearing a new scaffold as a backup medication for the treatment of microbial infections.

Nature is rich in natural products that have been utilized for the discovery of new scaffolds for various health problems and many drugs have been developed and used in modern medicine that originated from a natural product [8].

Chalcones are a polyphenolic natural product belonging to the flavonoid family and are key intermediates in flavonoid biosynthesis [9]. Chalcones are 1,3-diaryl propenone, which comprises two aryl moieties separated by three carbon atoms, furnishing α,β-unsaturated ketone derivatives. Chalcones have been reported to exhibit a wide range of pharmacological activities such as antimicrobial [10,11,12,13], anticancer [14,15,16], anti-inflammatory [17,18,19], antiviral [20], and antioxidant [21,22]. Furthermore, heterocyclic compounds each bearing a pyrazoline scaffold have been reported as antimicrobial [23,24,25], anticancer [26,27,28], anti-inflammatory [29,30,31], antiviral [32], and antioxidant [33].

**Scheme 1:**
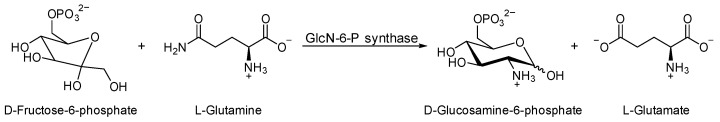
GlcN-6-P synthase catalyzes the biosynthesis of D-glucosamine-6-phosphate.

**Figure 1:**
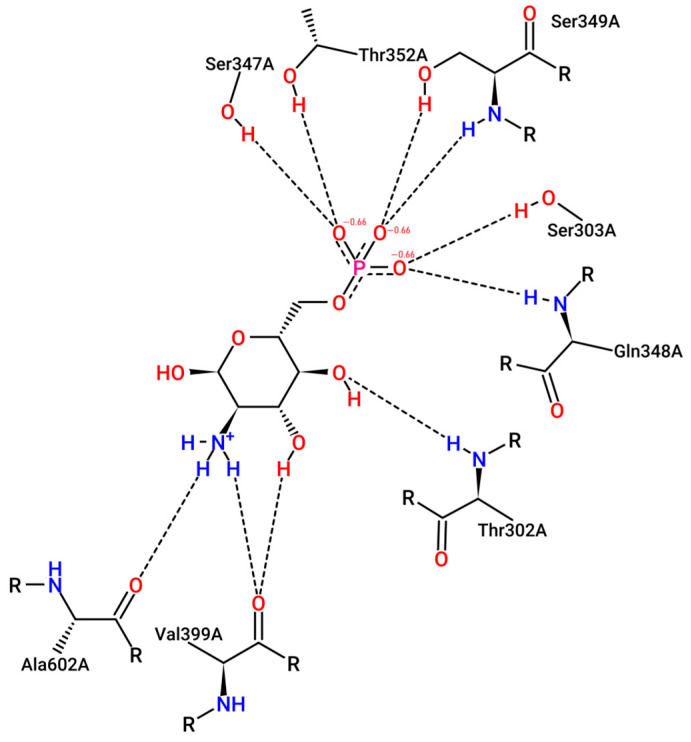
Two-dimensional diagrams of GlcN-6-P synthase in complex with the physiological ligand (glucosamine-6-phosphate) binding in the active site. Generated using an online website of pose view at https://proteins.plus/ (accessed on 17 December 2023).

Glucosamine-6-phosphate synthase (GlcN-6-P synthase), the molecular target enzyme in antimicrobial therapy [34], catalyzes the biotransformation of fructose-6-phosphate (Fru-6-P) and glutamine, as an amine source, to produce uridine diphosphate-*N*-acetylglucosamine (UDP-GlcNAc) [35]; see Scheme 1. *N*-Acetylglucosamine is a vital compound for cell membrane construction in a microorganism [36]. The binding pocket of the target enzyme (GlcN-6-P synthase) comprises Thr302, Ser303, Ser347, Gln348, Ser349, Thr352, Val399, and Ala602. The pose view of the active binding site is illustrated in Figure 1.

In the current study, novel chalcones bearing a furan ring and their corresponding 3,5-diaryl-∆^2^-pyrazoline derivatives were synthesized and characterized. In order to explore the biological activity of the newly synthesized compounds, they were screened against several microbial species, such as two Gram-positive species (*Staphylococcus aureus* and *Staphylococcus epidermidis*), two Gram-negative species (*Escherichia coli* and *Klebsiella pneumoniae*), and fungi (*Candida albicans*). The most active compounds in vitro were subjected to a molecular docking study targeting GlcN-6-P synthase.

## 2. Results and Discussion

### 2.1. Chemistry

**Scheme 2:**
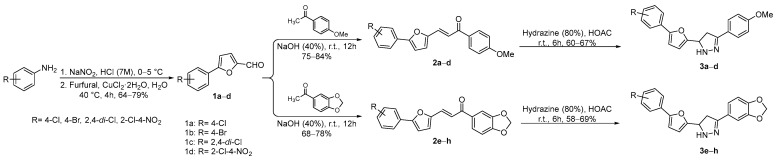
Synthesis of intermediates (**1a**–**d**) and target compounds (**2a**–**h** and **3a**–**h**).

The intermediate compounds (5-aryl-2-furaldehyde derivatives, **1a**–**d**) were accessed following Meerwein procedure. In the first step, arylamines (ArNH_2_) were diazotized with sodium nitrite in seven molar hydrochloric acid at 0–5 °C for 10 min, followed by the addition of furfural and cuprous chloride in water as solvent. The temperature was raised to 40 °C and the reaction mixture let to stir for 4 h. The desired products (**1a**–**d**) were obtained in good isolated yields. The chalcone compounds (**2a**–**h**) were synthesized via Claisen–Schmidt condensation reaction of equimolar concentration between acetophenones (*p*-methoxy acetophenone and 3,4-(methylenedioxy)acetophenone) and the aldehydes (**1a**–**d**) in the presence of 40% sodium hydroxide in ethanol as solvent at rt for 12 h, furnishing chalcones (**2a**–**h**) in good isolated yields. In the final step, the substituted chalcones (**2a**–**h**) were treated with hydrazine hydrate (80%) in the presence of glacial acetic acid as catalyst, yielding pyrazoline derivatives (**3a**–**h**) in moderate isolated yields; see Scheme 2.

The structures of the twenty newly synthesized compounds (**1a**–**d**, **2a**–**h** and **3a**–**h**) were characterized and confirmed via spectral data analysis, including IR, ^1^H-NMR, and GC-MS spectra. As an example, the structure characterization of compounds **1a**, **2a**, and **3a** is considered in the following discussion. The IR spectrum of the intermediate compound (**1a**) showed absorption at 3100 cm^−1^, which is due to aromatic C-H stretching, while the band around 2830 cm^−1^ represents C-H aldehyde stretching. The C=O absorption band appeared at 1670 cm^−1^. The bands around 1600 cm^−1^ relate to the C=C absorption. Moreover, ^1^H-NMR of **1a** showed two doublet signals, each one proton, at 6.83 and 7.32 ppm, reflecting the furan protons at position 3 and 4, while the other two doublet signals, each two protons, at 7.41 and 7.75 ppm represent the AA′BB′ system of the aryl ring. The aldehyde proton appeared as a singlet at 9.65 ppm. In addition, the mass spectra (EI) showed *m*/*z* at 206 Dalton, which represents the mass in the positive mode (M^+^) and further confirms the structure of compound **1a**. The IR analysis of chalcone **2a** showed stretching absorption around 3100 cm^−1^, which is related to aromatic C-H, while the stretching absorption of C=O appeared around 1650 cm^−1^. The ^1^H-NMR of **2a** showed new signals for the α,β-unsaturated carbonyl and the *p*-methoxy-phenyl moieties. A singlet signal at 3.89 ppm, with integration of three protons (3H), was related to the methoxy group. The multiplet signal at 7.54–7.58 ppm related to two aromatic protons as well as one proton of CH=CHC=O. The other CH proton of the α,β-unsaturated carbonyl appeared as a doublet at 7.73 ppm. Furthermore, the two doublet signals at 7.96 and 8.15 ppm related to the new AA′BB′ system of the *p*-methoxy-phenyl moiety. The IR spectrum of the pyrazoline product **3a** showed a new signal around 3350 cm^−1^ for N-H absorption, while the new addition of C=N of the heterocyclic ring showed a band around 1600 cm^−1^. The structures were further confirmed via ^1^H-NMR spectra, which did not reveal the presence of CH=CHC=O signals of the chalcone moiety. In addition, it exhibited three signals at 3.18, 3.30–3.37, and 4.86–4.91 ppm that are related to the new installment of a heterocyclic ring, which is in agreement with the cyclization of chalcone to ∆^2^-pyrazoline. Spectra of IR, ^1^H-NMR, and GC-MS are provided in the Supplementary Materials on pages S1–S10, S11–S20, and S21–S30, respectively.

### 2.2. Biology

The in vitro activities of the newly synthesized compounds (**2a**–**h** and **3a**–**h**) were conducted against microbial species, including Gram-positive and Gram-negative bacteria and fungi. These were tested and compared with the known standard drugs, amoxicillin and fluconazole, for antibacterial and antifungal activity, respectively. The chalcone derivatives (**2a**, **2b**, **2f**, and **2g**) showed moderate to good activity against all tested microbial species. Chalcone **2c** showed moderate activity against Gram-negative bacterial species (*Escherichia coli* and *Klebsiella pneumonia*) and strong activity against *Staphylococcus aureus* (Gram-positive) with no activity on the fungi (*Candida albicans*), while chalcone **2d** showed moderate activity vs. Gram-negative (*Escherichia coli* and *Klebsiella pneumonia*) species, Gram-positive (*Staphylococcus epidermidis*) species, and fungi (*Candida albicans*).

The minimal inhibitory concentration (MIC) determinations for the potent derivatives (**2a**–**c**, **2e**, **2f**, and **2h**) were conducted and revealed that compounds **2a**, **2b**, and **2c** exhibit antibacterial activity in lower concentrations (256 μg/mL) against *Staphylococcus aureus*, while compounds **2a** and **2c** were less potent, at 512 and 1024 μg/mL, respectively, against *Escherichia coli,* see Table 1. Moreover, the novel ∆^2^-pyrazoline derivatives (**3a** and **3b**) showed weak but selective activity vs. Gram-negative species (*Escherichia coli* and *Klebsiella pneumonia*), while **3c** showed weak activity but was selective for *Klebsiella pneumonia*. Finally, compound **3d** was the most active on several microbial species, namely *Staphylococcus epidermidis*, *Escherichia coli*, *Klebsiella pneumonia*, and *Candida albicans*; see Table 1. The Figures that illustrate the inhibition zones of the potent derivatives are provided in the Supplementary Materials on pages S31–S33.

### 2.3. Docking Study

The molecular docking simulation study of the potent active derivatives **2a, 2h**, and **3d** inside the active site of glucosamine-6-phosphate synthase, the potential target for antibacterial and antifungal agents, was explored by using AutoDock 4.2 software. The AutoDock tool allows the flexible docking of ligands into the site of action of proteins such as enzymes. It has the ability to use all the rotatable bonds of the ligands to give a number of conformations (10 by default) from which the best mode could be explored. As indicated by the X-ray crystallography, the binding pocket of glucosamine-6-phosphate synthase includes the following residues: Thr302, Ser303, Ser347, Gln348, Ser349, Thr352, Val399, and Ala602 (Figure 1). Autodock 4.2 was used to estimate the binding affinity, inhibition constant (*K*_i_), and other parameters of the selected active compounds (**2a**, **2h**, and **3d**) inside the known 3D structure of the target enzyme (Figure 1). The binding energy of the best-generated conformers (the first conformer) for the most active compounds (**2a**, **2h**, and **3d**) were −7.91, −8.31, and −7.45 Kcal·mol^−1^, respectively.

**Figure 2:**
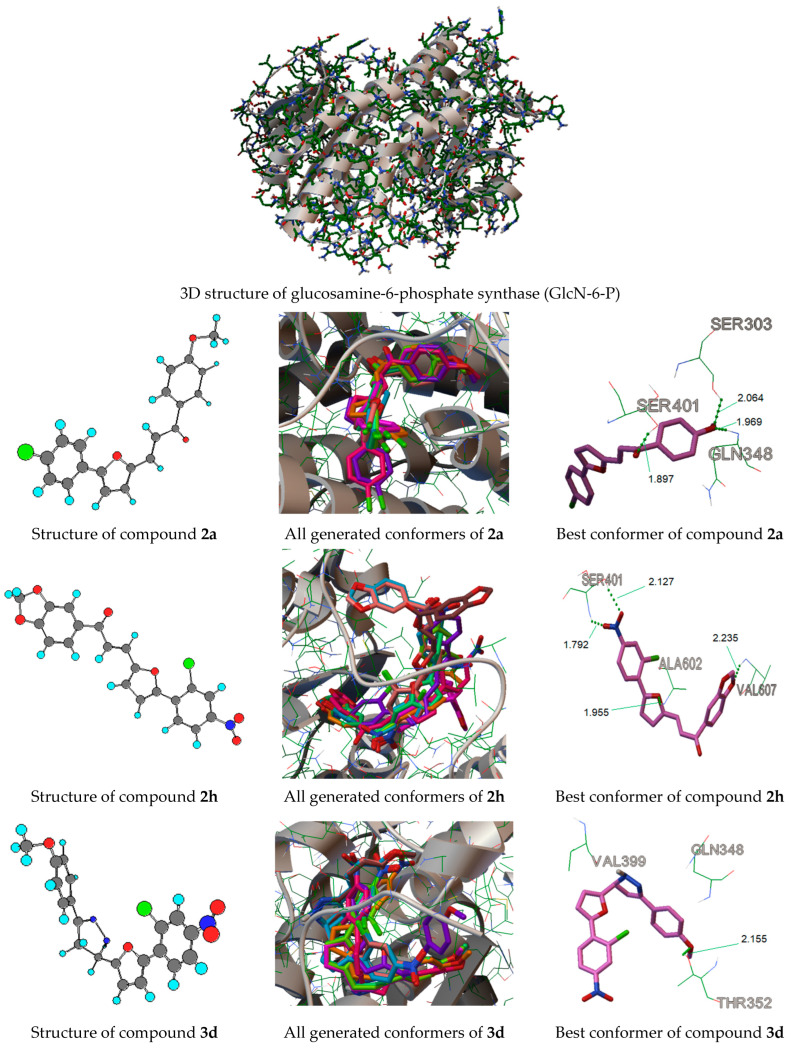
Three-dimensional structure of glucosamine-6-phosphate synthase (GlcN-6-P) and the docking of the best-generated conformers of the potent discovered hits **2a**, **2h**, and **3d** inside the binding pocket of GlcN-6-P. The green dot lines represent the H bonds in angstrom (Å).

The calculated *K*_i_ values for these compounds were found to be 1.60, 0.80 × 10^−3^, and 2.96 μM, respectively. The generated best conformers of compounds **2a**, **2h**, and **3d** are provided in Figure 2. As illustrated in Figure 2, the first constructed conformer of compound **2a** binds to the active site of the enzyme GlcN-6-P with three hydrogen bonds involving three amino acids; these are Ser303, Ser401, and Gln348, with a binding energy of −7.91 Kcal·mol^−1^ and a *K*_i_ value of 1.60 µM. The best score energy was shown by the first generated conformer of compound **2h** (−8.31 Kcal·mol^−1^) with its *K*_i_ value in the subpicomolar range (0.8 × 10^−3^ μM). The first conformer exhibited four hydrogen bonds with three amino acid residues: these are Ser401, Ala602, and Val607. Furthermore, the first generated conformer of compound **3d** was the least active hit (binding energy equal to −7.45 Kcal·mol^−1^) and was found to bind to the active site with one hydrogen bond involving amino acid Thr352. The docking results suggest that the potent discovered hits bind to the active site of the enzyme in a similar way to the substrate. In silico approaches reveal that the discovered hits (**2a**, **2h**, and **3d)** are promising inhibitors for targeting GlcN-6-P synthase, and the docking results are in agreement with in vitro antimicrobial evaluation. The docking parameters are provided in the Supplementary Materials on pages S34–S36.

## 3. Materials and Methods

### 3.1. Materials

The starting materials and solvents used in this study were purchased from commercial suppliers and were used without any purification. The melting ranges of the synthesized compounds were accomplished by using an electro thermal capillary apparatus and were uncorrected. The infrared spectrum (FT-IR) was measured using a Bruker ALPHA II FTIR Spectrometer-PLATINUM-ATR in the range of (400–4000 cm^−1^). The mass spectrum was collected on Shimadzu model GCMS-QP2010 PLUS. The ^1^H-NMR spectra of the prepared compounds were recorded on a Bruker Avance Neo (400 MHz) NMR spectrometer using the solvent peak as standard, with δ 2.5 and 7.26 ppm DMSO-*d*_6_ and CDCl_3_, respectively.

### 3.2. Chemistry

#### 3.2.1. General Procedure A: Synthesis of 5-Aryl-furan-2-carboxaldehyde Derivatives (**1a**–**d**)

The synthesis of aldehydes (**1a**–**d**) were accessed following a published procedure [37,38]. 4-Substituted anilines (0.136 mol) were dissolved in hydrochloric acid (56.2 mL, 7M) prepared from mixing conc. HCl (33.7 mL) and water (22.5 mL). The solution temperature was brought to 0 °C using an ice-water bath and the aniline derivatives were diazotized at 0–5 °C with a solution of sodium nitrite (9.5 g, 0.138 mol) in water (25 mL). The mixture was stirred for another 10 min, filtered off, and the filtrate was treated with furan-2-carboxaldehyde (15.4 g, 0.160 mol) dissolved in water (50 mL), followed by the addition of a solution of CuCl_2_·2H_2_O (5.0 g, 0.03 mol) in water (25 mL) at 10–15 °C. The resulting mixture was then stirred for 4 h at 40 °C. The formed precipitate was filtered off using a Büchner funnel, then washed with water, an aqueous solution of sodium hydrogen carbonate (5%), and water, each 20 mL. The precipitate was air-dried under suction filtration and recrystallized from ethanol.

#### 3.2.2. General Procedure B: Synthesis of Chalcone Compounds (**2a**–**h**)

Eight chalcone compounds (**2a**–**h**) were newly synthesized via a Claisen–Schmidt condensation reaction using a modified published procedure [24]. In brief, sodium hydroxide (40%, 1 mL) was added to a stirred solution of 1 mmol acetophenone derivatives (4-methoxy or 3,4-(methylenedioxy)acetophenone) in ethanol (10 mL). The resulting mixture was stirred at rt for 30 min. This was followed by the addition of equimolar concentration (1 mmol) of the prepared 5-aryl-2-furaldehydes (**1a**–**d**) in the previous step (general procedure A), and stirring continued at rt for 12 h. Upon completion of the reaction, monitored via TLC using ethyl acetate/hexane (1:2) as the eluent, ice-cold water was added and the precipitate filtered off, washed with water (3 × 10 mL), air-dried, and recrystallized from ethanol.

#### 3.2.3. General Procedure C: Synthesis of 3,5-Diaryl-∆^2^-pyrazoline Derivatives (**3a**–**h**)

The new ∆^2^-pyrazoline derivatives were synthesized in analogy to our previously published procedure [24,39,40]. To a solution of chalcone derivatives (0.01 mol) in ethanol (10 mL) was added glacial acetic acid (7 drops). The resulting mixture was stirred for 15 min at rt followed by dropwise addition of hydrazine hydrate (80%, 2.42 mL, 0.05 mol), and the reaction was let to stir at rt for 6h. TLC using ethyl acetate/hexane (2:1) as the eluent was used to monitor the reaction progress. After reaction completion, the formed precipitate was filtered off, washed with diethyl ether (3 × 10 mL), air-dried, and recrystallized from ethanol.

### 3.3. Spectral Data of All Synthesized Compounds (**1a**–**d**, **2a**–**h**, and **3a**–**h**)

5-(4-Chlorophenyl)furan-2-carboxaldehyde (**1a**).

Following general procedure A, there was a 64% yield of dark brown solid, m.p. 114–116 °C; FT-IR (cm^−1^): 3110 (C-H furan), 3059 (C-H aromatic), 2834 (C-H aldehyde), 1673 (C=O), 1661 (C=C furan ring), 1589 (C=C aromatic ring), 1010 (C-Cl). ^1^H-NMR (400 MHz, CDCl_3_) δ (ppm): 6.83 (d, 1H, CH furan, *J* = 4.0 Hz), 7.32 (d, 1H, CH furan, *J* = 4.0 Hz), 7.41 (d, 2H, Ar-H, *J* = 8.0 Hz), 7.75 (d, 2H, Ar-H, *J* = 8.0 Hz), 9.65 (s, 1H, CHO). GC-MS (EI) *m*/*z*: 206 M^+^ for C_11_H_7_ClO_2_.

5-(4-Bromophenyl)furan-2-carboxaldehyde (**1b**).

Following general procedure A, there was a 65% yield of brown solid, m.p. 143–145 °C; FT-IR (cm^−1^): 3110 (C-H furan), 3056 (C-H aromatic), 2858 (C-H aldehyde), 1672 (C=O), 1660 (C=C furan ring), 1595 (C=C aromatic ring), 1039 (C-Br). ^1^H-NMR (400 MHz, CDCl_3_) δ (ppm): 6.84 (d, 1H, CH furan, *J* = 4.0 Hz), 7.32 (d, 1H, CH furan, *J* = 4.0 Hz), 7.57 (d, 2H, Ar-H, *J* = 8.0 Hz), 7.68 (d, 2H, Ar-H, *J* = 8.0 Hz), 9.65 (s, 1H, CHO). GC-MS (EI) *m*/*z*: 250 M^+^ for C_11_H_7_BrO_2_.

5-(2,4-Dichlorophenyl)furan-2-carboxaldehyde (**1c**).

Following general procedure A, there was a 79% yield of brown powder, m.p. 148–150 °C; FT-IR (cm^−1^): 3158 (C-H furan), 3079 (C-H aromatic), 2842 (C-H aldehyde), 1677 (C=O), 1663 (C=C furan ring), 1585 (C=C aromatic ring), 1034 (C-Cl). ^1^H-NMR (400 MHz, CDCl_3_) δ (ppm): 7.32 (d, 1H, Ar-H, *J* = 12.0 Hz), 7.37 (d, 1H, CH furan *J* = 4.0 Hz), 7.50 (d, 1H, CH furan *J* = 4.0 Hz), 7.96 (d, 1H, Ar-H, *J* = 12.0 Hz), 7.35 (s, 1H, Ar-H), 9.70 (s, 1H, CHO). GC-MS (EI) *m*/*z*: 240 M^+^ for C_11_H_6_Cl_2_O_2_.

5-(2-Chloro-4-nitrophenyl)furan-2-carboxaldehyde (**1d**).

Following general procedure A, there was a 75% yield of orange solid, m.p. 124–126 °C; FT-IR (cm^−1^): 3105 (C-H furan), 3034 (C-H aromatic), 2832 (C-H aldehyde), 1680 (C=O), 1664 (C=C furan ring), 1582 (C=C aromatic ring), 1341sym.,1513asym. (NO2), 1034 (C-Cl). ^1^H-NMR (400 MHz, CDCl_3_) δ (ppm): 7.41 (d, 1H, CH furan, *J* = 4.0 Hz), 7.56 (d, 1H, CH furan, *J* = 4.0 Hz), 8.22 (d, 1H, Ar-H, *J* = 8.0 Hz), 8.25 (d, 1H, Ar-H, *J* = 8.0 Hz), 8.38 (s, 1H, Ar-H), 9.77 (s, 1H, CHO). GC-MS (EI) *m*/*z*: 251 M^+^ for C_11_H_6_ClNO_4_.

3-(5-(4-Chlorophenyl)furan-2-yl)-1-(4-methoxyphenyl)prop-2-en-1-one (**2a**).

Following general procedure B, there was a 75% yield of yellow solid, m.p. 169–171 °C; FT-IR (cm^−1^): 3109 (C-H aromatic), 2839 (C-H aliphatic), 1646 (C=O chalcone), 1603 (CH=CH chalcone), 1587 (C=C aromatic), 1031 (C-Cl). ^1^H-NMR (400 MHz, DMSO-d_6_) δ (ppm): 3.89 (s, 3H, OCH_3_), 7.11 (d, 2H, Ar-H, *J* = 8.0 Hz), 7.19 (d, 1H, CH furan, *J* = 4.0 Hz), 7.24 (d, 1H, CH furan, *J* = 4.0 Hz), 7.55 (d, 2H, Ar-H, *J* = 8.0 Hz), 7.57–7.75 (m, 2H, CH chalcone), 7.96 (d, 2H, Ar-H, *J* = 8.0 Hz), 8.15 (d, 2H, Ar-H, *J* = 8.0 Hz). GC-MS (EI) *m*/*z*: 338 M^+^ for C_20_H_15_ClO_3_.

3-(5-(4-Bromophenyl)furan-2-yl)-1-(4-methoxyphenyl)prop-2-en-1-one (**2b**).

Following general procedure B, there was a 76% yield of yellow solid, m.p. 185–187 °C; FT-IR (cm^−1^): 3109 (C-H aromatic), 2939 (C-H aliphatic), 1646 (C=O chalcone), 1599 (CH=CH chalcone), 1587 (C=C aromatic), 1029 (C-Br). ^1^H-NMR (400 MHz, DMSO-d_6_) δ (ppm): 3.88 (s, 3H, OCH_3_), 7.11 (d, 2H, Ar-H, *J* = 8.0 Hz), 7.20 (d, 1H, CH furan, *J* = 4.0 Hz), 7.27 (d, 1H, CH furan, *J* = 4.0 Hz), 7.56 (d, 1H, C*H*=CH-C=O, *J* = 16.0 Hz), 7.69 (d, 2H, Ar-H, *J* = 8.0 Hz), 7.75 (d, 1H, CH=C*H*-C=O, *J* = 16.0 Hz), 7.91 (d, 2H, Ar-H, *J* = 8.0 Hz), 8.16 (d, 2H, Ar-H, *J* = 8.0 Hz). GC-MS (EI) *m*/*z*: 383 M^+^ for C_20_H_15_BrO_3_.

3-(5-(2,4-Dichlorophenyl)furan-2-yl)-1-(4-methoxyphenyl)prop-2-en-1-one (**2c**).

Following general procedure B, yield 84% of yellow solid, m.p. 114–116 °C; FT-IR (cm^−1^): 3112 (C-H aromatic), 2836 (C-H aliphatic), 1648 (C=O chalcone), 1603 (CH=CH chalcone), 1583 (C=C aromatic), 1023 (C-Cl). ^1^H-NMR (400 MHz, DMSO-d_6_) δ (ppm): 3.88 (s, 3H, OCH_3_), 7.11 (d, 2H, Ar-H, *J* = 8.0 Hz), 7.25 (d, 1H, CH furan, *J* = 4.0 Hz), 7.39 (d, 1H, CH furan, *J* = 4.0 Hz), 7.54–7.57 (m, 1H, CH=C*H*-C=O and 1H, CH, Ar-H), 7.75–7.77 (m, 1H, CH=C*H*-C=O and 1H, CH, Ar-H), 8.12 (d, 2H, Ar-H, *J* = 8.0 Hz), 8.20 (d, 1H, Ar-H, *J* = 8.0 Hz). GC-MS (EI) *m*/*z*: 373 M^+^ for C_20_H_14_Cl_2_O_3_.

3-(5-(2-Chloro-4-nitrophenyl)furan-2-yl)-1-(4-methoxyphenyl)prop-2-en-1-one (**2d**).

Following general procedure B, there was a 80% yield of orange solid, m.p. 164–166 °C; FT-IR (cm^−1^): 3110 (C-H aromatic), 2838 (C-H aliphatic), 1648 (C=O chalcone), 1605 (CH=CH chalcone), 1583 (C=C aromatic), 1339sym., 1510asym. (NO_2_), 1019 (C-Cl). ^1^H-NMR (400 MHz, DMSO-d_6_) δ (ppm): 3.88 (s, 3H, OCH_3_), 7.11 (d, 2H, Ar-H, *J* = 8.0 Hz), 7.31 (d, 1H, CH furan, *J* = 4.0 Hz), 7.59 (d, 1H, C*H*=CH-C=O, *J* = 16.0 Hz), 7.65 (d, 1H, CH furan, *J* = 4.0 Hz), 7.84 (d, 1H, CH=C*H*-C=O, *J* = 16.0 Hz), 8.15 (d, 2H, Ar-H, *J* = 8.0 Hz), 8.25–8.46 (m, 3H, Ar-H). GC-MS (EI) *m*/*z*: 384 M^+^ for C_20_H_14_ClO_5_.

1-(Benzo[d][1,3]dioxol-5-yl)-3-(5-(4-chlorophenyl)furan-2-yl)prop-2-en-1-one (**2e**).

Following general procedure B, there was a 73% yield of yellow solid, m.p, 165–167 °C; FT-IR (cm^−1^): 3108 (C-H aromatic), 2902 (C-H aliphatic), 1644 (C=O chalcone), 1603 (CH=CH chalcone), 1580 (C=C aromatic), 1042 (C-Cl). ^1^H-NMR (400 MHz, DMSO-d_6_) δ (ppm): 6.18 (s, 2H, CH_2_), 7.12 (d, 1H, Ar-H, *J* = 8.0 Hz), 7.20 (d, 1H, CH furan, *J* = 4.0 Hz), 7.255 (d, 1H, CH furan, *J* = 4.0 Hz), 7.55 (d, 2H, Ar-H, *J* = 4.0 Hz), 7.57 (d, 2H, Ar-H, *J* = 4.0 Hz), 7.65 (d, 1H, CH, Ar-H, *J* = 4.0 Hz), 7.70 (s, 1H, CH, Ar-H), 7.86 (d, 1H, C*H*=CH-C=O, *J* = 8.0 Hz), 7.98 (d, 1H, CH=C*H*-C=O, *J* = 8.0 Hz). GC-MS (EI) *m*/*z*: 352 M^+^ for C_20_H_13_ClO_4_.

1-(Benzo[d][1,3]dioxol-5-yl)-3-(5-(4-bromophenyl)furan-2-yl)prop-2-en-1-one (**2f**).

Following general procedure B, there was a 68% yield of yellow solid, m.p. 180–182 °C; FT-IR (cm^−1^): 3106 (C-H aromatic), 2937 (C-H aliphatic), 1643 (C=O chalcone), 1602 (CH=CH chalcone), 1581 (C=C aromatic), 1022 (C-Br). ^1^H-NMR (400 MHz, DMSO-*d*_6_) δ (ppm): 6.18 (s, 2H, CH_2_), 7.11 (d, 1H, Ar-H, *J* = 8.0 Hz), 7.20 (d, 1H, CH furan, *J* = 4.0 Hz), 7.27 (d, 1H, CH furan, *J* = 4.0 Hz), 7.55 (d, 2H, Ar-H, *J* = 16.0 Hz), 7.67 (d, 1H, C*H*=CH-C=O, *J* = 12.0 Hz), 7.70 (s, 1H, Ar-H), 7.72 (d, 2H, Ar-H, *J* = 16.0 Hz), 7.86 (d, 1H, Ar-H, *J* = 8.0 Hz), 7.92 (d, 1H, CH=C*H*-C=O, *J* = 12.0 Hz). GC-MS (EI) *m*/*z*: 397 M^+^ for C_20_H_13_BrO_4_.

1-(Benzo[d][1,3]dioxol-5-yl)-3-(5-(2,4-dichlorophenyl)furan-2-yl)prop-2-en-1-one (**2g**).

Following general procedure B, there was a 75% yield of yellow solid, m.p. 158–160 °C; FT-IR (cm^−1^): 3111 (C-H aromatic), 2900 (C-H aliphatic), 1645 (C=O chalcone), 1606 (CH=CH chalcone), 1576 (C=C aromatic), 1024 (C-Cl). ^1^H-NMR (400 MHz, DMSO-*d*_6_) δ (ppm): 6.18 (s, 2H, CH_2_), 7.11 (d, 1H, Ar-H, *J* = 8.0 Hz), 7.26 (d, 1H, CH furan, *J* = 4.0 Hz), 7.40 (d, 1H, CH furan, *J* = 4.0 Hz), 7.56 (s, 1H, Ar-H), 7.58–7.64 (d, 2H, C*H*=CH-C=O and Ar-H), 7.76 (d, 1H, CH=C*H*-C=O, *J* = 16.0 Hz), 7.80 (d, 1H, Ar-H, *J* = 4.0 Hz), 7.85 (d, 1H, Ar-H, *J* = 8.0 Hz), 8.23 (d, 1H, Ar-H, *J* = 8.0 Hz). GC-MS (EI) *m*/*z*: 387 M^+^ for C_20_H_12_Cl_2_O_4_.

1-(Benzo[d][1,3]dioxol-5-yl)-3-(5-(2-chloro-4-nitrophenyl)furan-2-yl)prop-2-en-1-one (**2h**).

Following general procedure B, there was a 78% yield of yellow solid, m.p. 211–213 °C; FT-IR (cm^−1^): 3105 (C-H aromatic), 2911 (C-H aliphatic), 1650 (C=O chalcone), 1607 (CH=CH chalcone), 1586 (C=C aromatic), 1352sym., 1477asym. (NO_2_), 1036 (C-Cl). ^1^H-NMR (400 MHz, DMSO-*d*_6_) δ (ppm): 6.19 (s, 2H, CH_2_), 7.13 (d, 1H, Ar-H, *J* = 8.0 Hz), 7.34 (d, 1H, CH furan, *J* = 4.0 Hz), 7.61 (d, 1H, C*H*=CH-C=O, *J* = 16.0 Hz), 7.56 (d, 1H, Ar-H, *J* = 4.0 Hz), 7.69 (d, 1H, CH furan, *J* = 4.0 Hz), 7.86 (d, 1H, CH=C*H*-CO, *J* = 16.0 Hz), 8.29–8.52 (m, 3H, Ar-H). GC-MS (EI) *m*/*z*: 397 M^+^ for C_20_H_12_ClNO_6_..

5-(5-(4-Chlorophenyl)furan-2-yl)-3-(4-methoxyphenyl)-4,5-dihydro-1*H*-pyrazole (**3a**).

Following general procedure C, there was a 60% yield of white solid, m.p. 122–124 °C; FT-IR (cm^−1^): 3352 (NH pyrazoline), 3124 (C-H aromatic), 2935 (C-H aliphatic), 1605 (C=N pyrazoline), 1592 (C=C aromatic), 1025 (C-Cl). ^1^H-NMR (400 MHz, DMSO-*d*_6_) δ (ppm): 3.175 (dd, 1H, Ha-pyrazoline, *J* = 12.0, 16.0 Hz), 3.36 (d,1H,Hb-pyrazoline, *J* = 8.0 Hz), 3.78 (s, 3H, OCH_3_), 4.86–4.91 (t, 1H, Hx-pyrazoline, *J* = 8.0 Hz), 6.49 (d, 1H, CH furan, *J* = 4.0 Hz), 6.95 (d, 1H, CH furan, *J* = 4.0 Hz), 6.97 (d, 2H, Ar-H, *J* = 8.0 Hz), 6.47 (d, 2H, Ar-H, *J* = 8.0 Hz), 7.61 (d, 2H, Ar-H, *J* = 12.0 Hz), 7.70 (d, 2H, Ar-H, *J* = 8.0 Hz). GC-MS (EI) *m*/*z*: 352 M^+^ for C_20_H_17_ClN_2_O_2_.

5-(5-(4-Bromophenyl)furan-2-yl)-3-(4-methoxyphenyl)-4,5-dihydro-1*H*-pyrazole (**3b**).

Following general procedure C, there was a 62% yield of light yellow solid, m.p. 138–140 °C; FT-IR (cm^−1^): 3349 (NH pyrazoline), 3172 (C-H aromatic), 2932 (C-H aliphatic), 1605 (C=N pyrazoline), 1568 (C=C aromatic), 1023 (C-Br). ^1^H-NMR (400 MHz, DMSO-*d*_6_) δ (ppm): 3.17 (dd, 1H, Ha-pyrazoline, *J* = 8.0, 16.0 Hz), 3.34 (d, 1H, Hb-pyrazoline, *J* = 12.0 Hz), 3.78 (s, 3H, OCH_3_), 4.86–4.91 (t, 1H, Hx-pyrazoline, *J* = 8.0 Hz), 6.49 (d, 1H, CH furan, *J* = 4.0 Hz), 6.96–7.65 (m, 1H, CH furan and 8H, Ar-H), 7.44 (bs, 1H, NH pyrazoline). GC-MS (EI) *m*/*z*: 397 M^+^ for C_20_H_17_BrN_2_O_2_.

5-(5-(2,4-Dichlorophenyl)furan-2-yl)-3-(4-methoxyphenyl)-4,5-dihydro-1*H*-pyrazole (**3c**).

Following general procedure C, there was a 67% yield of light yellow solid, m.p. 112–114 °C; FT-IR (cm^−1^): 3297 (NH pyrazoline), 3077 (C-H aromatic), 2889 (C-H aliphatic), 1602 (C=N pyrazoline), 1553 (C=C aromatic), 1024 (C-Cl). ^1^H-NMR (400 MHz, DMSO-*d*_6_) δ (ppm): 3.205 (dd, 1H, Ha-pyrazoline, *J* = 8.0, 16.0 Hz), 3.31 (d, 1H, Hb-pyrazoline ring, *J* = 16.0 Hz), 3.78 (s, 3H, OCH_3_), 4.91 (bs, 1H, Hx-pyrazoline), 6.56 (d, 1H, CH furan, *J* = 4.0 Hz), 6.97 (d, 2H, Ar-H, *J* = 12.0 Hz), 7.11 (d, 1H, CH furan, *J* = 4.0 Hz), 7.50–7.87 (m, 4H, Ar-H). GC-MS (EI) *m*/*z*: 387 M^+^ for C_20_H_16_Cl_2_N_2_O_2_.

5-(5-(2-Chloro-4-nitrophenyl)furan-2-yl)-3-(4-methoxyphenyl)-4,5-dihydro-1*H*-pyrazole (**3d**).

Following general procedure C, there was a 64% yield of yellow solid, m.p 179–181 °C; FT-IR (cm^−1^): 3316 (NH pyrazoline), 3158 (C-H aromatic), 2914 (C-H aliphatic), 1606 (C=N pyrazoline), 1585 (C=C aromatic), 1338sym., 1509asym. (NO_2_), 1026 (C-Cl). ^1^H-NMR (400 MHz, DMSO-*d*_6_) δ (ppm): 3.23 (dd, 1H, Ha-pyrazoline, *J* = 8.0, 16.0 Hz), 3.42 (d, 1H, Hb-pyrazoline, *J* = 16.0 Hz), 3.78 (s, 3H, OCH_3_), 4.93–4.98 (t, 1H, Hx-pyrazoline, *J* = 8.0 Hz), 6.67 (d, 1H, CH furan, *J* = 4.0 Hz), 6.97 (d, 2H, Ar-H, *J* = 8.0 Hz), 7.43 (d, 1H, CH furan, *J* = 4.0 Hz), 7.54 (s, 1H, NH pyrazoline), 7.61 (d, 2H, Ar-H, *J* = 8.0 Hz), 8.10–8.36 (m, 3H, Ar-H). GC-MS (EI) *m*/*z*: 398 M^+^ for C_20_H_16_ClN_3_O_4_.

3-(Benzo[d][1,3]dioxol-5-yl)-5-(5-(4-chlorophenyl)furan-2-yl)-4,5-dihydro-1*H*-pyrazole (**3e**).

Following general procedure C, there was a 58% yield of light yellow solid, m.p. 121–123 °C; FT-IR (cm^−1^): 3333 (NH pyrazoline), 3169 (C-H aromatic), 2900 (C-H aliphatic), 1610 (C=N pyrazoline), 1572 (C=C aromatic), 1038 (C-Cl). ^1^H-NMR (400 MHz, DMSO-*d*_6_) δ (ppm): 3.17 (dd, 1H, Ha-pyrazoline, *J* = 12.0, 16.0 Hz), 3.34 (dd, 1H, Hb-pyrazoline, *J* = 12.0, 16.0 Hz), 4.86–4.92 (t, 1H, Hx-pyrazoline, *J* = 12.0 Hz), 6.06 (s, 2H, O-CH_2_-O), 6.49 (d, 1H, CH furan, *J* = 4.0 Hz), 6.94–6.96 (m, 1H, CH furan and 1H, Ar-H) 7.11 (d, 1H, Ar-H, *J* = 8.0 Hz), 7.47 (d, 2H, Ar-H, *J* = 8.0 Hz), 7.70 (d, 2H, Ar-H, *J* = 8.0 Hz). GC-MS (EI) *m*/*z*: 366 M^+^ for C_20_H_15_ClN_2_O_3_.

3-(Benzo[d][1,3]dioxol-5-yl)-5-(5-(4-bromophenyl)furan-2-yl)-4,5-dihydro-1*H*-pyrazole (**3f**).

Following general procedure C, there was a 60% yield of light yellow solid, m.p. 136–138 °C; FT-IR (cm^−1^): 3339 (NH pyrazoline), 3119 (C-H aromatic), 2900 (C-H aliphatic), 1603 (C=N pyrazoline), 1586 (C=C aromatic), 1038 (C-Br). ^1^H-NMR (400 MHz, DMSO-*d*_6_) δ (ppm): 3.17 (dd, 1H, Ha-pyrazoline, *J* = 8.0, 16.0 Hz), 3.37 (d, 1H, Hb-pyrazoline, *J* = 12.0 Hz), 4.90 (bs, 1H, Hx-pyrazoline), 6.06 (s, 2H, O-CH_2_-O), 6.18 (s, 1H, NH pyrazoline), 6.49 (d, 1H, CH furan, *J* = 4.0 Hz), 6.94–7.11 (m, 3H, Ar-H), 7.26 (d, 1H, CH furan, *J* = 4.0 Hz), 7.60 (d, 2H, Ar-H, *J* = 8.0 Hz), 7.64 (d, 2H, Ar-H, *J* = 8.0 Hz). GC-MS (EI) *m*/*z*: 411 M^+^ for C_20_H_15_BrN_2_O_3_.

3-(Benzo[d][1,3]dioxol-5-yl)-5-(5-(2,4-dichlorophenyl)furan-2-yl)-4,5-dihydro-1*H*-pyrazole (**3g**).

Following general procedure C, there was a 69% yield of light yellow solid, m.p. 128–130 °C; FT-IR (cm^−1^): 3320 (NH pyrazoline), 3175 (C-H aromatic), 2958 (C-H aliphatic), 1609 (C=N pyrazoline), 1581 (C=C aromatic), 1020 (C-Cl). ^1^H-NMR (400 MHz, DMSO-*d*_6_) δ (ppm): 3.165 (d, 1H, Ha-pyrazoline, *J* = 4.0 Hz), 3.35 (dd, 1H, Hb-pyrazoline, *J* = 8.0, 16.0 Hz), 4.92 (bs, 1H, Hx-pyrazoline), 6.06 (s, 2H, O-CH_2_-O), 6.19 (s, 1H, NH pyrazoline), 6.56 (d, 1H, CH furan, *J* = 4.0 Hz), 6.95 (d, 1H, Ar-H, *J* = 8.0 Hz), 7.105 (d, 1H, CH furan, *J* = 4.0 Hz), 7.25 (s, 1H, Ar-H), 7.51 (d, 1H, Ar-H, *J* = 4.0 Hz), 7.53 (d, 1H, Ar-H, *J* = 4.0 Hz), 7.71 (s, 1H, Ar-H), 7.86 (d, 1H, Ar-H, *J* = 8.0 Hz). GC-MS (EI) *m*/*z*: 401 M^+^ for C_20_H_14_Cl_2_N_2_O_3_.

3-(Benzo[d][1,3]dioxol-5-yl)-5-(5-(2-chloro-4-nitrophenyl)furan-2-yl)-4,5-dihydro-1*H*-pyrazole (**3h**).

Following general procedure C, there was a 66% yield of orange solid, m.p. 170–172 °C; FT-IR (cm^−1^): 3271 (NH pyrazoline), 3101 (C-H aromatic), 2912 (C-H aliphatic), 1649 (C=N pyrazoline), 1584 (C=C aromatic), 1337sym., 1501asym. (NO_2_), 1037 (C-Cl). ^1^H-NMR (400 MHz, DMSO-*d*_6_) δ (ppm): 3.22 (dd, 1H, Ha-pyrazoline, *J* = 8.0, 16.0 Hz), 3.40 (dd, 1H, Hb-pyrazoline, *J* = 8.0, 16.0 Hz), 4.93–4.98 (t, 1H, Hx-pyrazoline, *J* = 8.0 Hz), 6.06 (s, 2H, O-CH_2_-O), 6.19 (s, 1H, NH pyrazoline), 3.84 (d, 1H, Ar-H, *J* = 8.0 Hz), 7.86 (s, 1H, Ar-H), 7.875 (d, 1H, CH furan, *J* = 4.0 Hz), 8.11 (d, 1H, Ar-H, *J* = 8.0 Hz), 8.26 (d, 1H, Ar-H, *J* = 8.0 Hz), 8.30 (d, 1H, CH furan, *J* = 4.0 Hz), 8.37–8.42 (dd, 1H, Ar-H), 8.49 (d, 1H, Ar-H, *J* = 8.0 Hz). GC-MS (EI) *m*/*z*: 411 M^+^ for C_20_H_14_ClN_3_O_5_.

### 3.4. Antimicrobial Studies

#### 3.4.1. Evaluation of Antimicrobial Activity

The synthesized compounds (**2a**–**h** and **3a**–**h**) were subjected to in vitro antimicrobial activity tests using the agar well-diffusion method [41] against four bacterial strains, namely *Staphylococcus aureus* and *Staphylococcus epidermidis* (Gram-positive), *Escherichia coli* and *Klebsiella pneumoniae* (Gram-negative), and fungi, *Candida albicans*. Amoxicillin and fluconazole (standard drugs) and the synthesized final compounds (**2a**–**h** and **3a**–**h**) were tested at concentration of 10 mg/mL dissolved in DMSO. In brief, the sterilized agar [Müller-Hinton agar] media were poured in petri dishes and let to solidify. The testing bacteria were distributed on the surface of the agar with sterile cotton swab, then holes were made in the agar and it was left for 15 min. After that, 50 µL from the tested compound solution was placed in the holes and the dishes were incubated at 37 °C for 24 h. The zone of inhibition observed around the holes was measured in millimeters (mm).

#### 3.4.2. Estimation of Minimal Inhibitory Concentration (MIC)

A serial dilution agar method was used for the MIC measurement for all compounds that showed good activity in the antimicrobial activity test using double-fold serial dilution: 2, 4, 8, 16, 32, 64, 128, 256, 512, and 1024 µg/mL to the most active compounds were freshly prepared from stock solution. In a separate manner, these dilutions were added to Müller-Hinton agar at 50 °C into sterile glass tubes, each alone and mixed very well, then poured into sterile plates. These plates were let cool down to 37 °C, then let solidify at rt. The prepared plates were kept in a refrigerator at 4 °C for use within 24 h. Some colonies (4–5) from the overnight bacteria culture were placed into 5 mL of normal saline for the preparation of bacterial suspension and were adjusted to 0.5 McFarland turbidity (equal to 1.5 × 10^8^ CFU/mL). A small amount (5 μL) of bacterial suspension was taken using a micropipette and used to inoculate plates, then left for 10 min. The plates were incubated at 37 °C for 24 h [42].

### 3.5. Docking Study

The binding affinity of the most potent derivatives (**2a**, **2h**, and **3d**) to the active site of GlcN-6-P synthase was examined using the AutoDock 4.2 bundle program. The PDB file of the target enzyme (GlcN-6-P synthase) was obtained from the Protein Data Bank website (PDB code 1MOQ, https://www.rcsb.org/structure/1MOQ (accessed on 17 December 2023)) and was employed as a static structure for the docking study. In the first step, water molecules were eliminated, the natural ligand was removed, and hydrogen atoms were added to the protein residues. ChemDraw Ultra 7.0 was used to create the two-dimensional structures of the new hits, and Open Babel 2.3.1 was employed to convert these structures to mol-formatted files. Following our previously published procedure [24], the three-dimensional axes (X, Y, and Z) were specified as 30.5, 17.5, and −2.2, respectively. The points are separated by 0.358 Å and were built on the center of the catalytic site of the enzyme. A Lamarckian genetic algorithm with 10 runs and 150-population size was used as the docking algorithm. By default, 2.5 × 10^5^ and 27 × 10^3^ generations worth of energy evaluations were optimized [24]. The accuracy and reproducibility of the docking protocol was validated. In brief, the co-crystalized ligand (GlcN-6-P) with the protein was removed from the binding site, prepared, and redocked into the active catalytic site of the enzyme. The ligand (GlcN-6-P) was found to fit back into its original position within the active site of the isolated protein.

## 4. Conclusions

Novel furan-derived chalcones and their ∆^2^-pyrazoline derivatives were synthesized, characterized, and evaluated as antimicrobial agents. The target compounds were obtained in multistep reaction procedures, which start with the chemical reaction between two substituted acetophenones and the newly synthesized furfural intermediates (**1a**–**d**). The chalcone derivatives (**2a**–**h**) were further treated with hydrazine hydrate to undergo cycloaddition reactions and to furnish the 3,5-diaryl-∆^2^-pyrazoline derivatives (**3a**–**h**). All synthesized products (**2a**–**h** and **3a**–**h**) were evaluated as antimicrobial agents against two Gram-positive (*Staphylococcus aureus* and *Staphylococcus epidermidis*), two Gram-negative (*Escherichia coli* and *Klebsiella pneumoniae*), and one fungi (*Candida albicans*) species. Two of the newly synthesized chalcone derivatives (**2a** and **2h**) were found to be active on all investigated microbial species, while the ∆^2^-pyrazoline compound **3d** was found to be selective versus certain microbial species. The most potent compounds (**2a**, **2h**, and **3d**) were docked in a GlcN-6-P synthase model in order to study their virtual affinity and binding mode with the enzyme. The active compounds were found to bind to the active site of the enzyme probably in a similar way to that of the substrate, as suggested by the docking study. In summary, the newly developed chalcones and their ∆^2^-pyrazoline derivatives could serve as potent leads toward the development of novel antimicrobial agents. In future work, it is necessary to synthesize other derivatives to further investigate the structure–activity relationships (SARs) and to explore the cytotoxicity of the synthesized derivatives.

## Figures and Tables

**Table 1:** Antimicrobial activity of the synthesized compounds (**2a**–**h** and **3a**–**h**) and standard drugs at concentration (10 mg/mL) and their (MICs) ^a^.

Diameter of Inhibition Zone (mm), (MIC μg/mL) ^a^							
Compd.	Gram-Positive Bacteria			Gram-Negative Bacteria			Fungi
	*Staphylococcus aureus*		*Staphylococcus epidermidis*	*Escherichia coli*		*Klebsiella pneumoniae*	*Candida albicans*
**2a**	15	(256) ^a^	10	12	(512) ^a^	12	14
**2b**	13	(256) ^a^	11	12		10	14
**2c**	16	(256) ^a^	–	10	(1024) ^a^	10	–
**2d**	–		12	12		12	15
**2e**	17		–	11		11	8
**2f**	16		11	11		11	10
**2g**	–		–	–		–	–
**2h**	17		12	12		12	10
**3a**	–		–	9		9	–
**3b**	–		–	9		9	–
**3c**	–		–	-		9	–
**3d**	–		10	9		9	9
**3e**	–		–	–		–	–
**3f**	–		–	–		–	–
**3g**	–		–	–		10	–
**3h**	–		–	–		–	–
**Amoxicilline**	32		27	20		18	n.d.
**Floconazol**	n.d.		n.d.	n.d.		n.d.	17

^a^ Activity note: no inhibition observed (–), weak activity (<10), moderate activity (12–15), strong activity > 15; n.d.: not determined.

## Data Availability

All data are provided in the Supporting Information.

## Supplementary Materials

The following supporting information can be downloaded at: https://www.mdpi.com/article/10.3390/antibiotics13010021/s1, Pages S1–S10 (FTIR), Pages S11–S20 (^1^H-NMR), Pages S21–S30 (MS), Pages S31–S33 inhibition zone, and Pages S34–S36 docking parameters.
