# Supplementary material for: Design, Synthesis, Antimicrobial Properties, and Molecular Docking of Novel Furan-Derived Chalcones and Their 3,5-Diaryl-∆2-pyrazoline Derivatives
Source: Antibiotics (Basel). 2023 Dec 24;13(1):21. doi: 10.3390/antibiotics13010021 (PMC10812776; doi:10.3390/antibiotics13010021)
Supplement: Supplementary file 1 [file antibiotics-13-00021-s001.zip › antibiotics-2702622-supplementary.pdf]

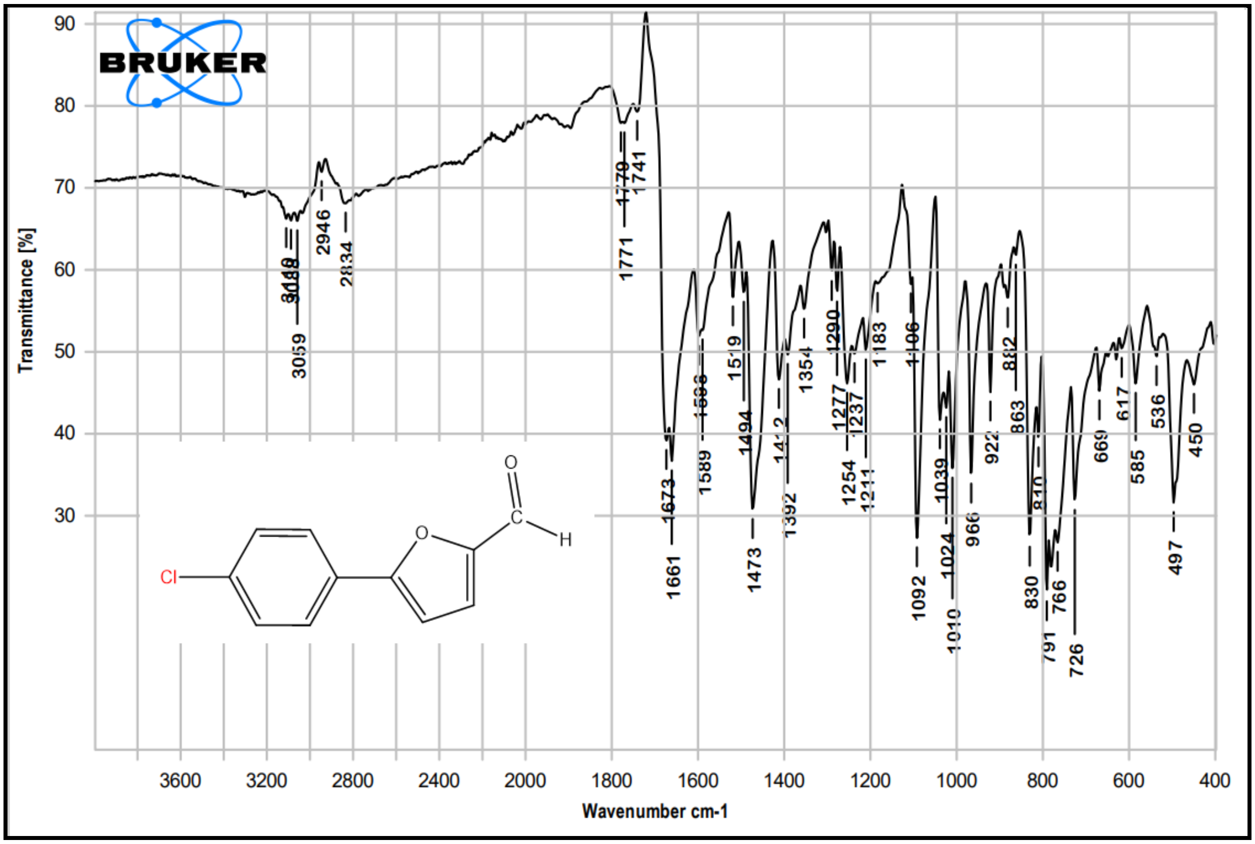

FTIR spectrum of compound 1a

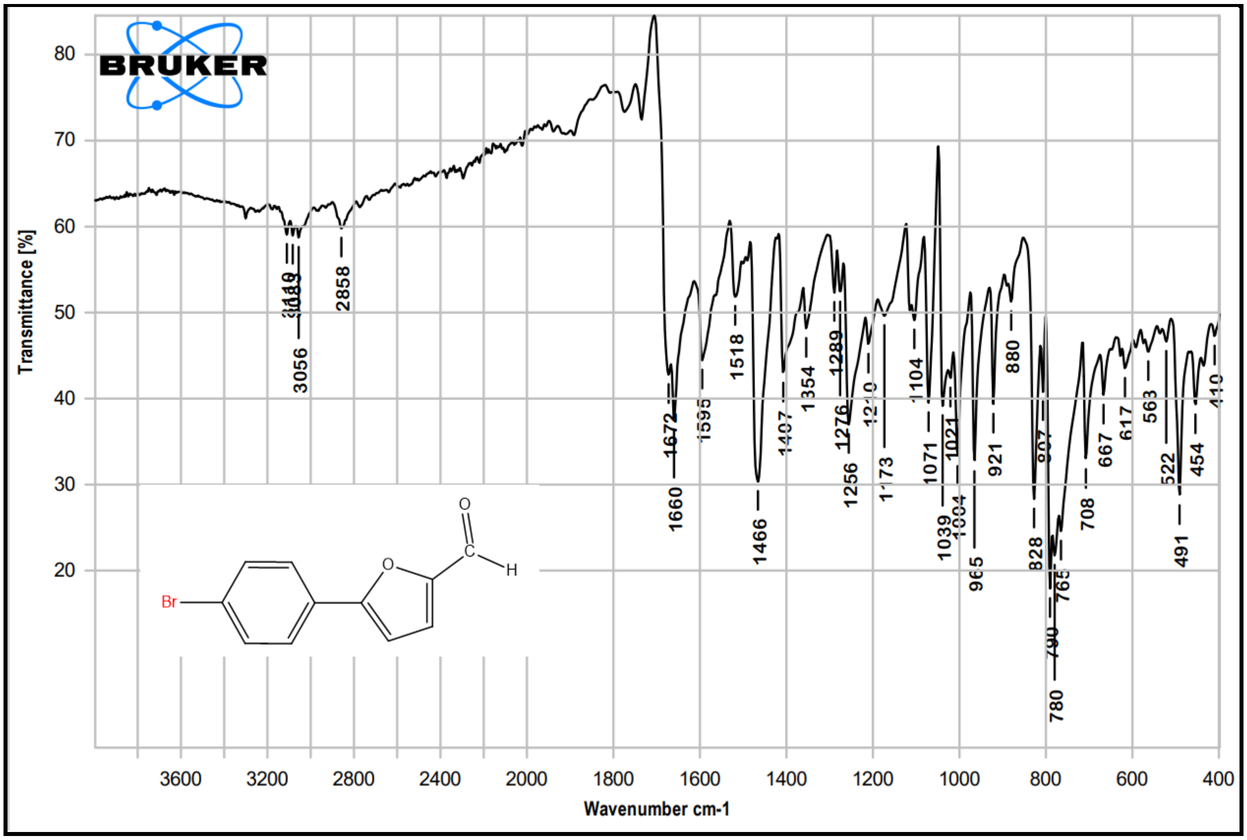

FTIR spectrum of compound 1b

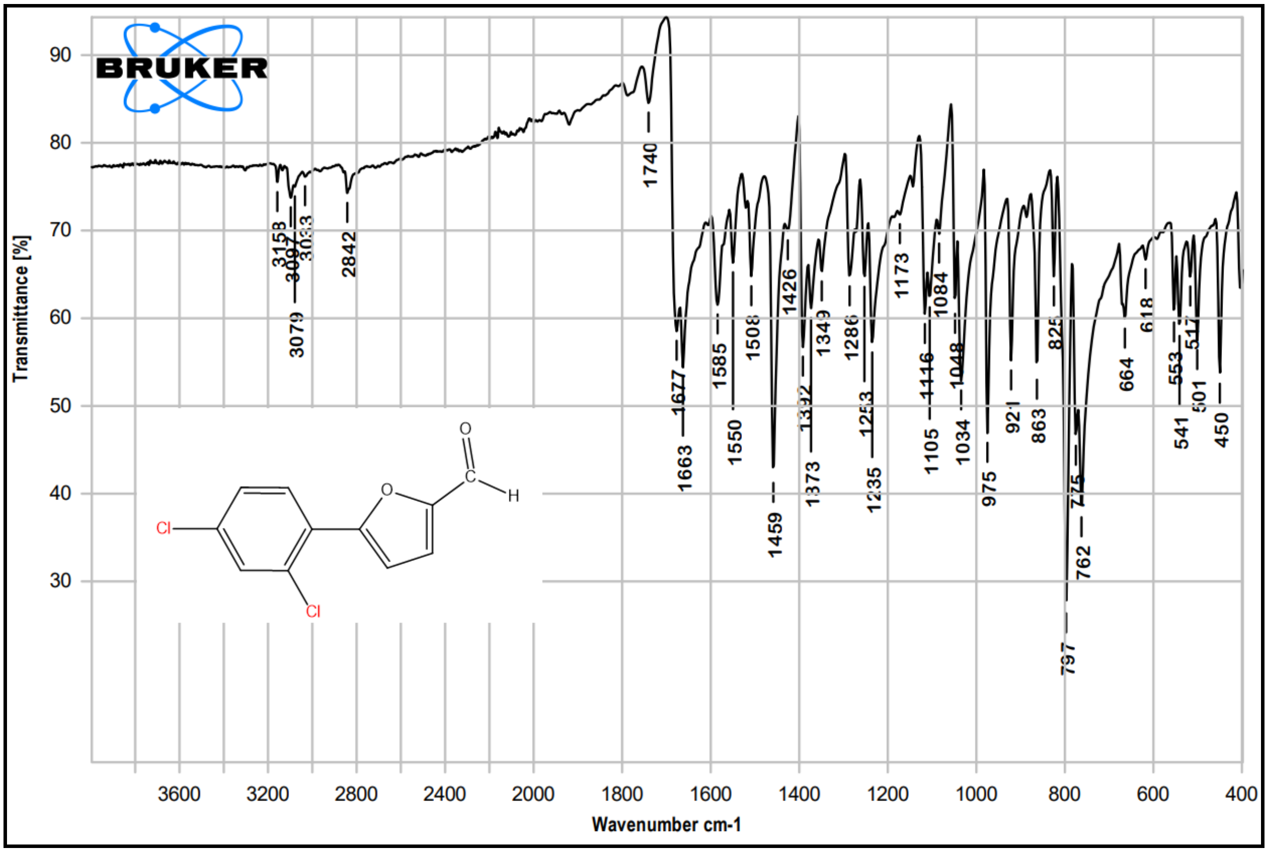

FTIR spectrum of compound 1c

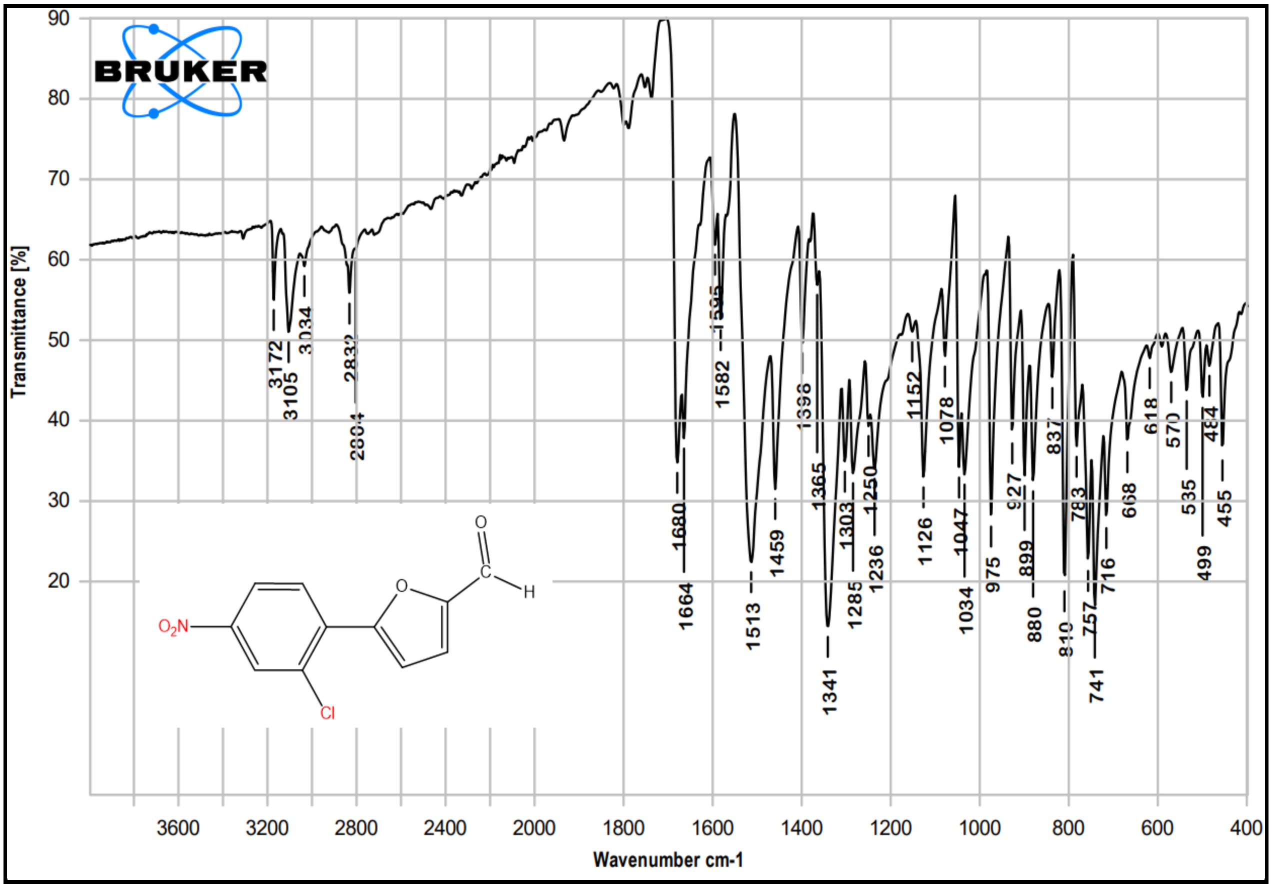

FTIR spectrum of compound 1d

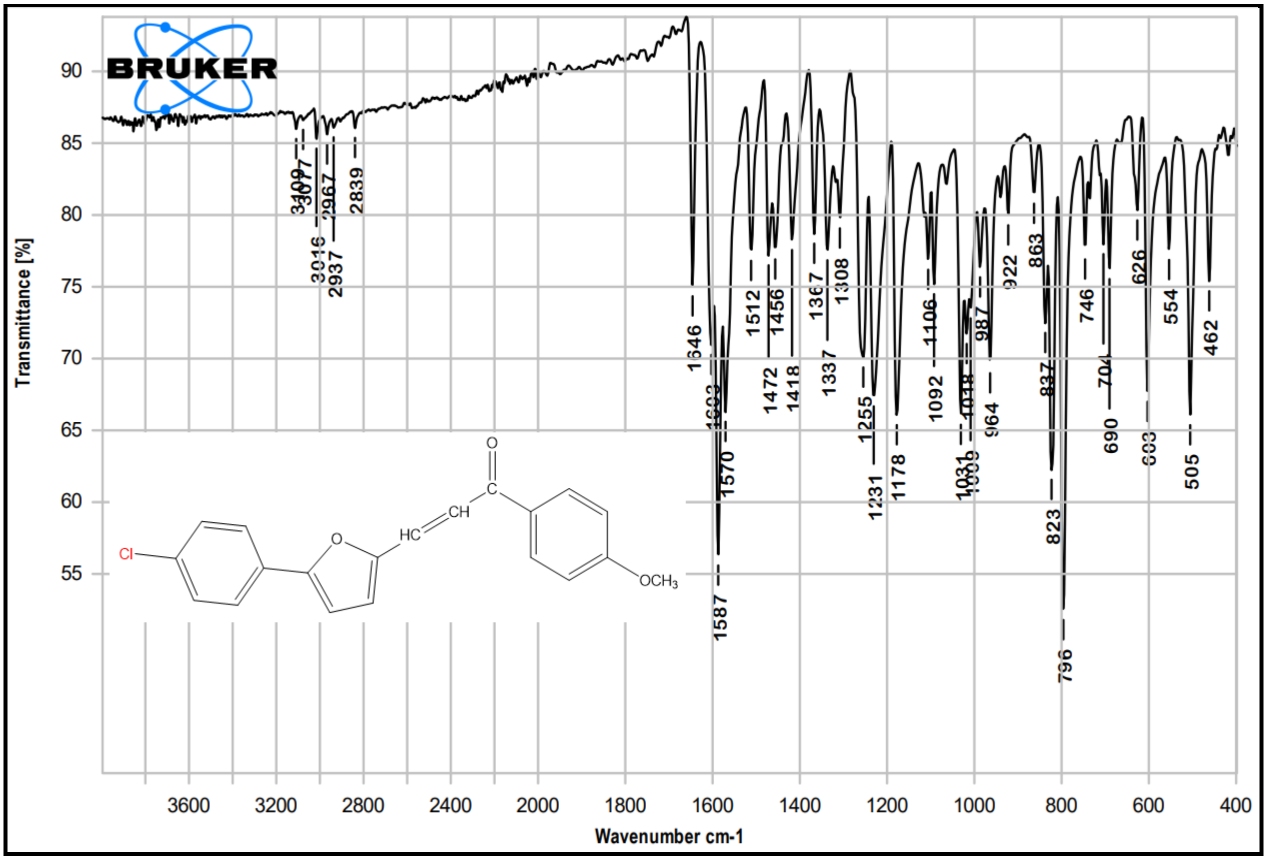

FTIR spectrum of compound 2a

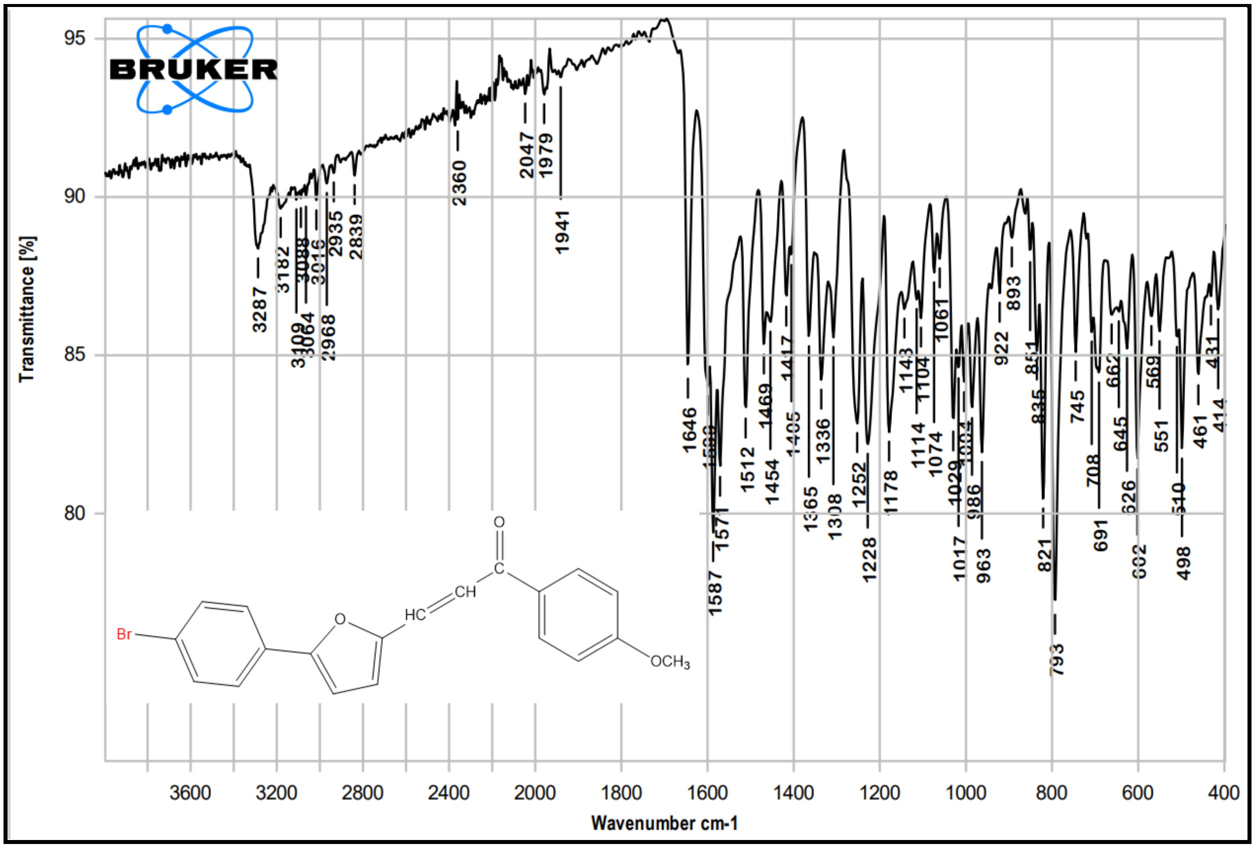

FTIR spectrum of compound 2b

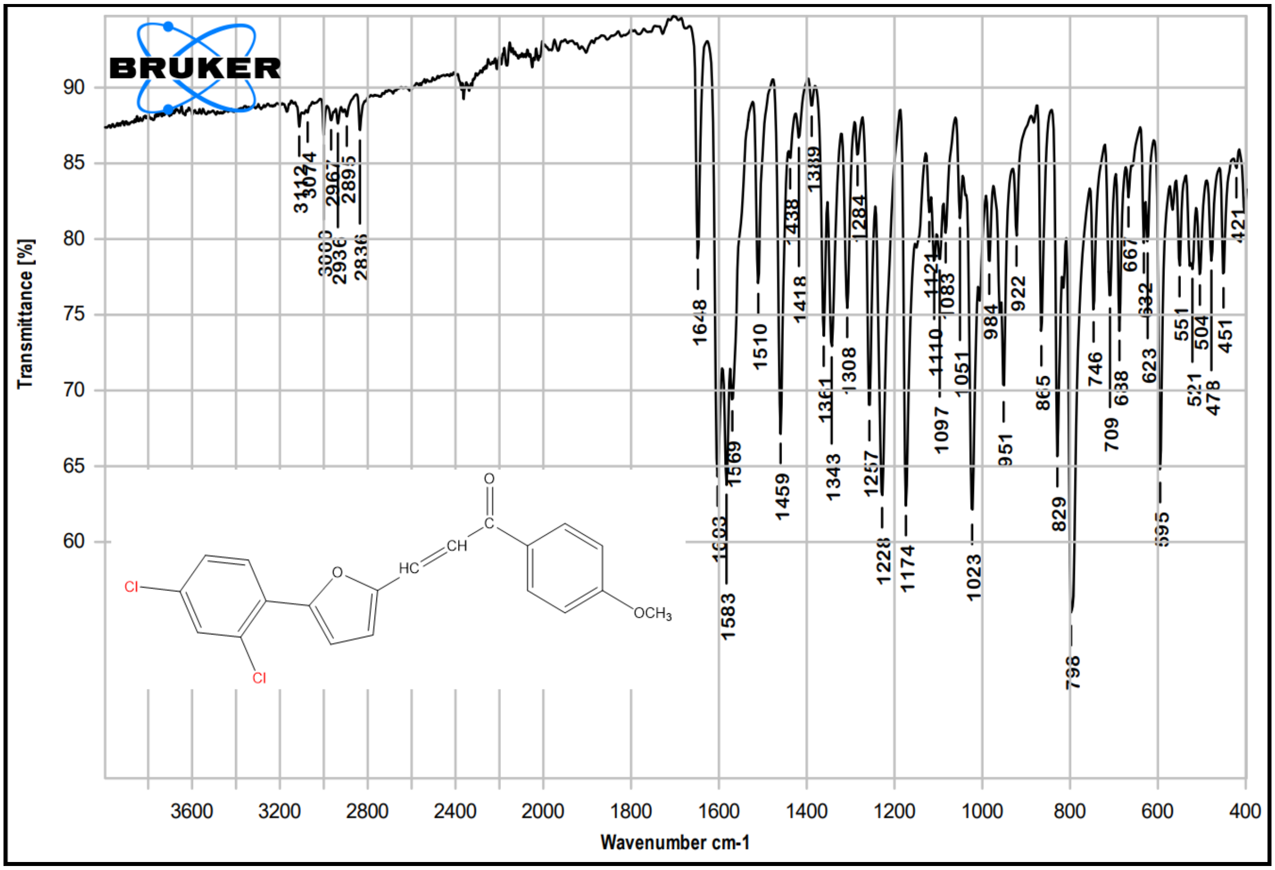

FTIR spectrum of compound 2c

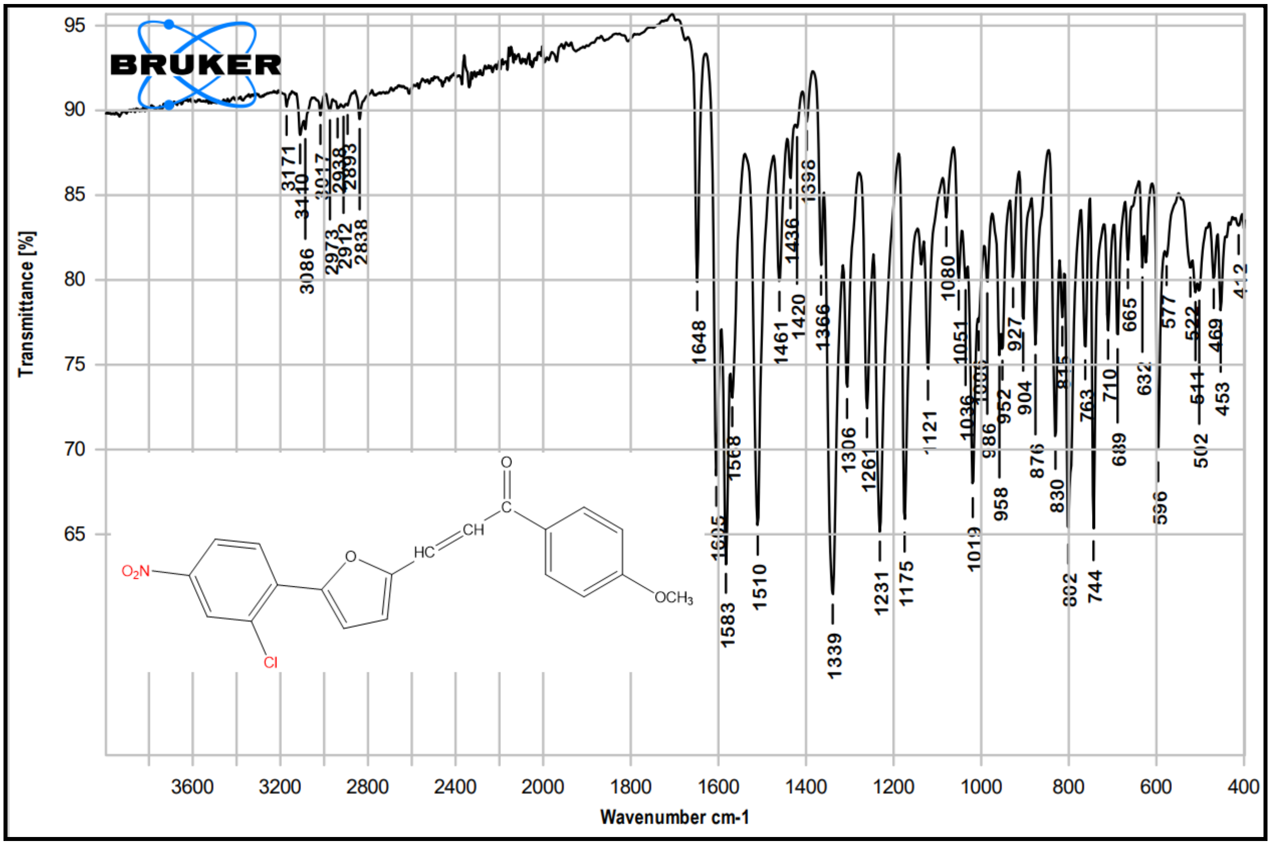

FTIR spectrum of compound 2d

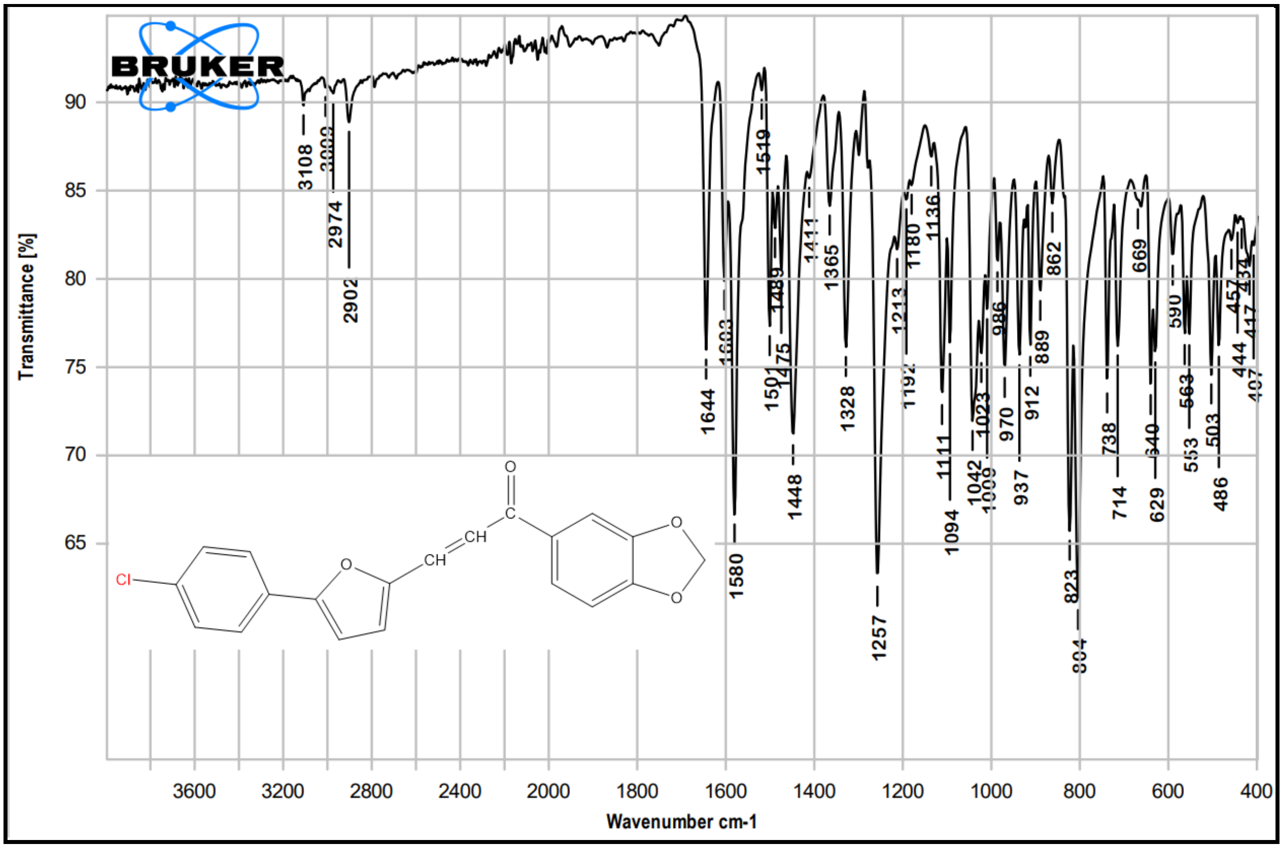

FTIR spectrum of compound 2e

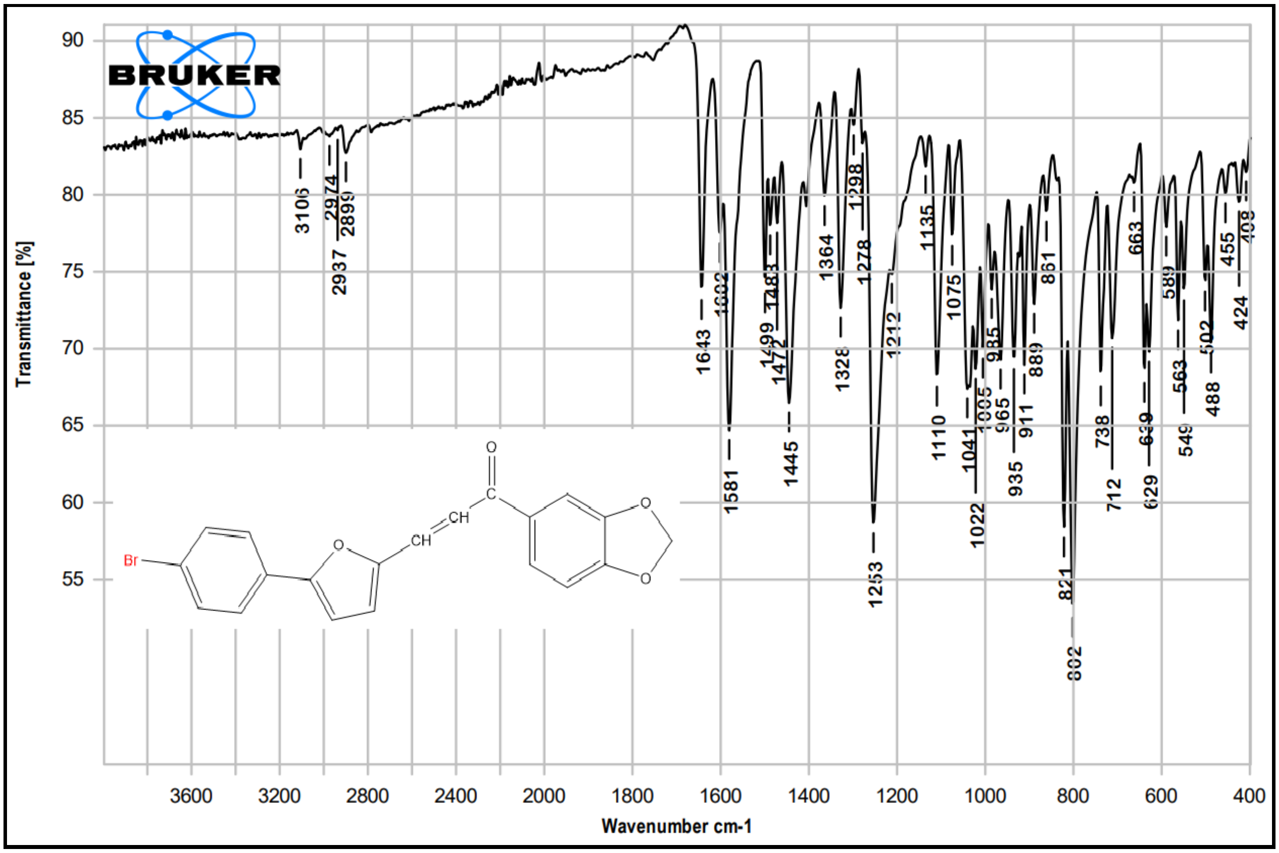

FTIR spectrum of compound 2f

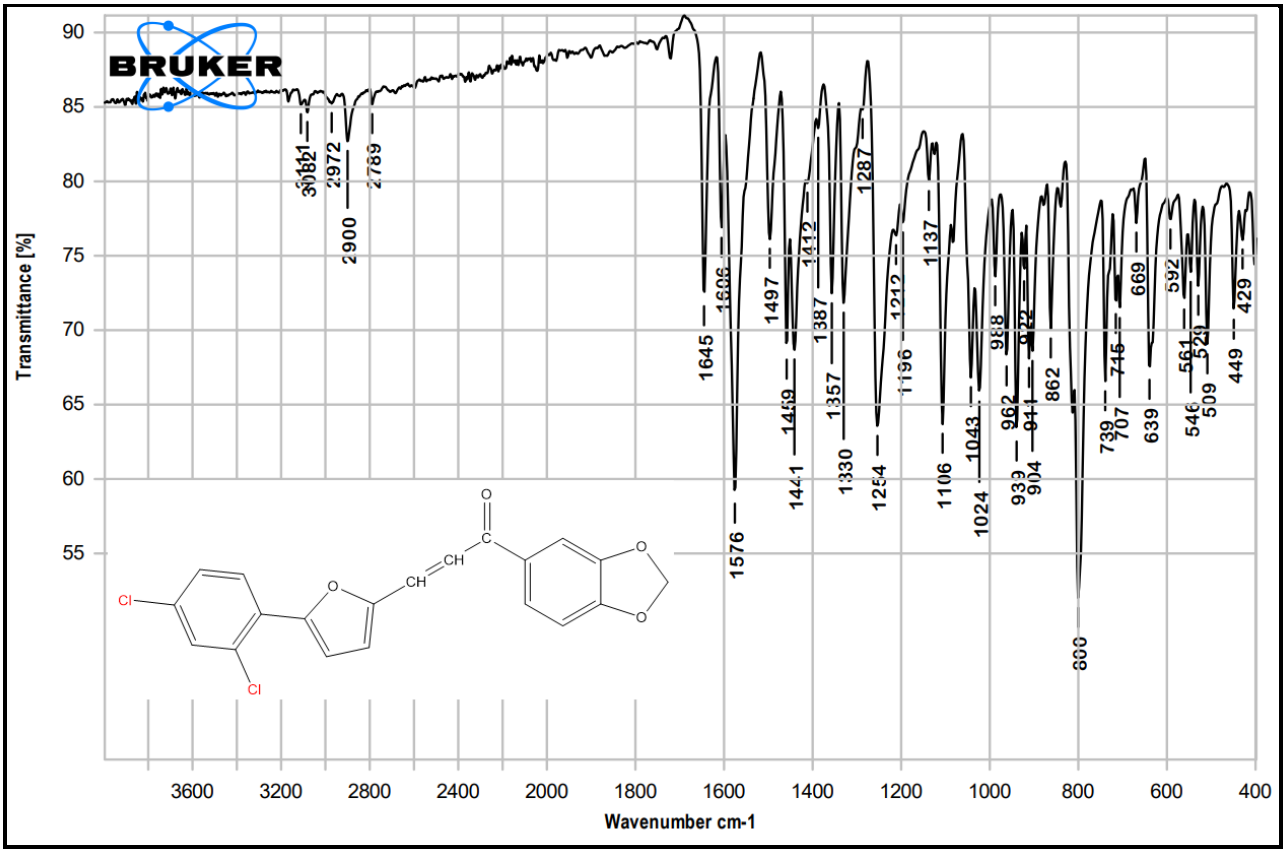

FTIR spectrum of compound 2g

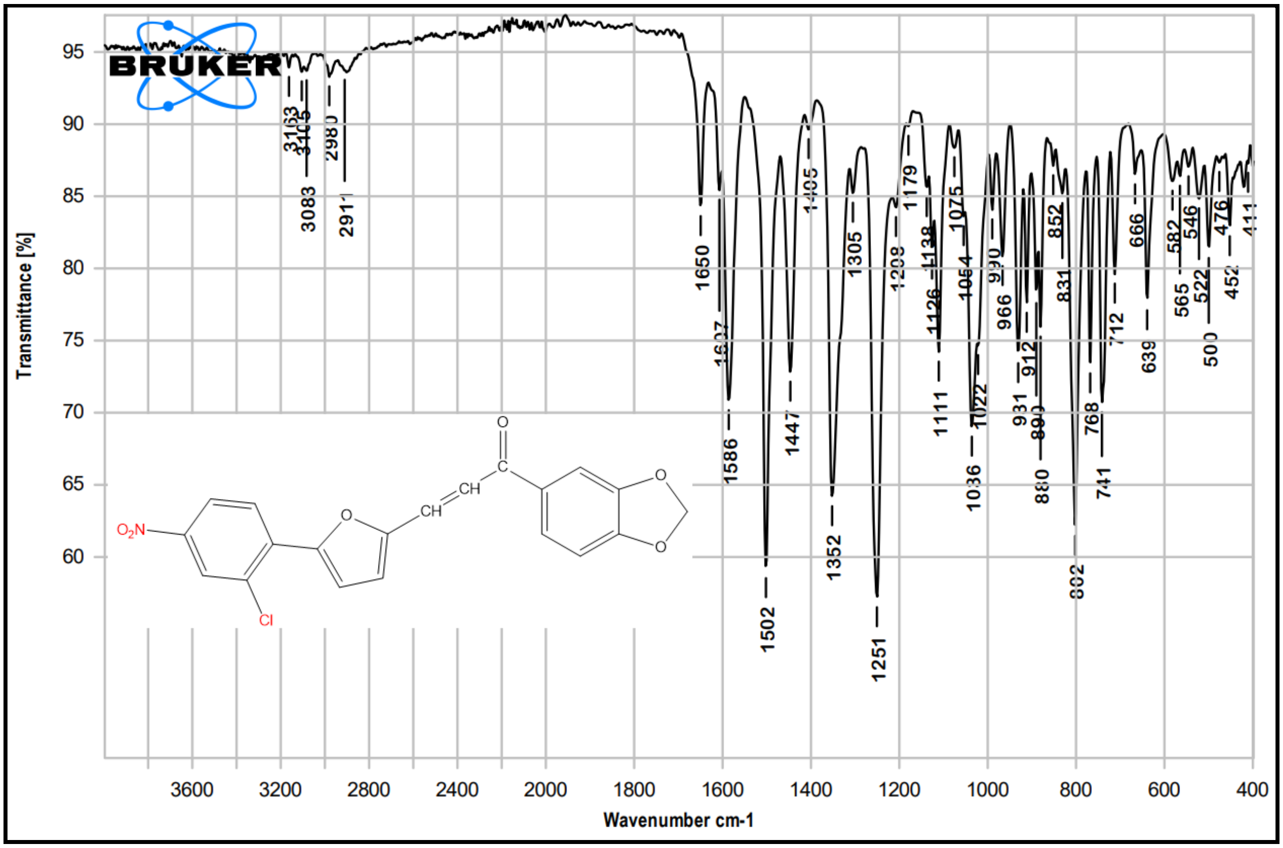

FTIR spectrum of compound 2h

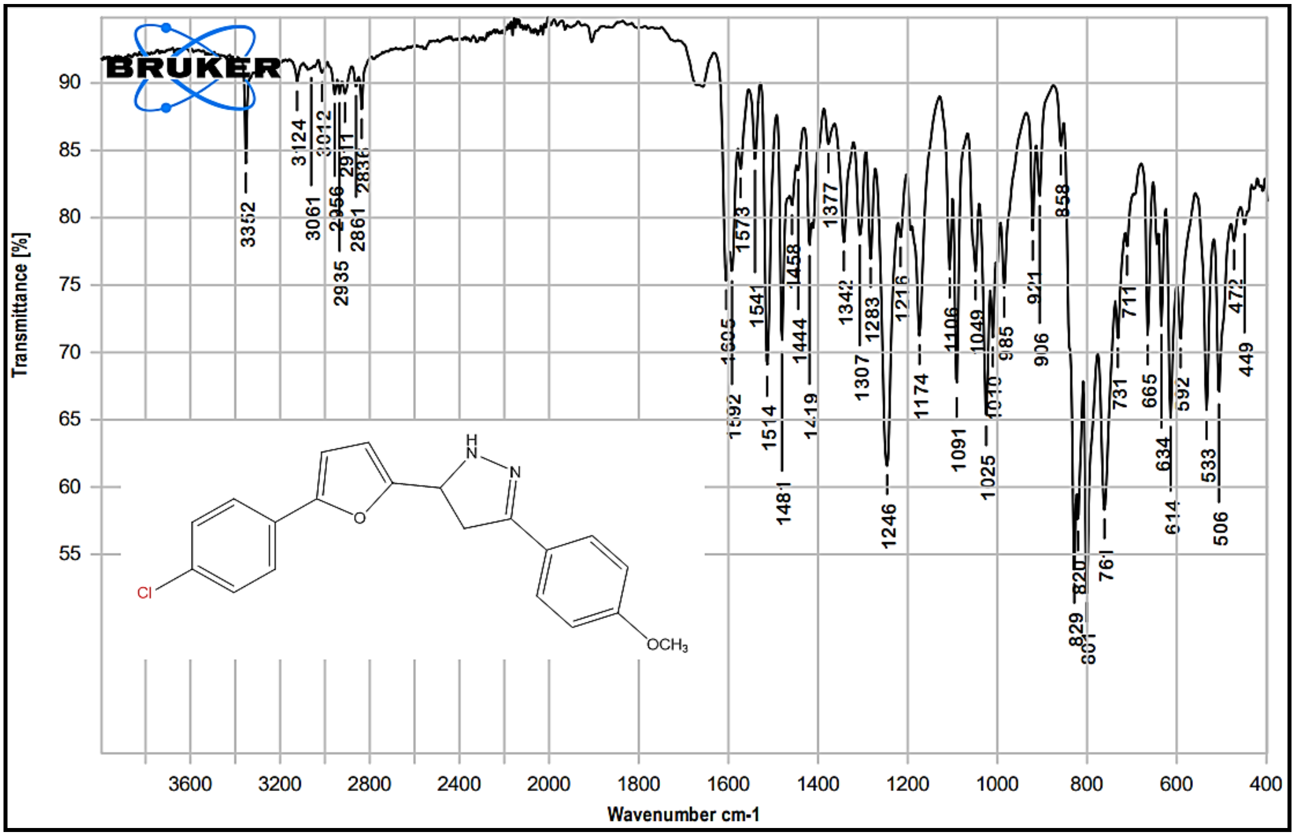

FTIR spectrum of compound 3a

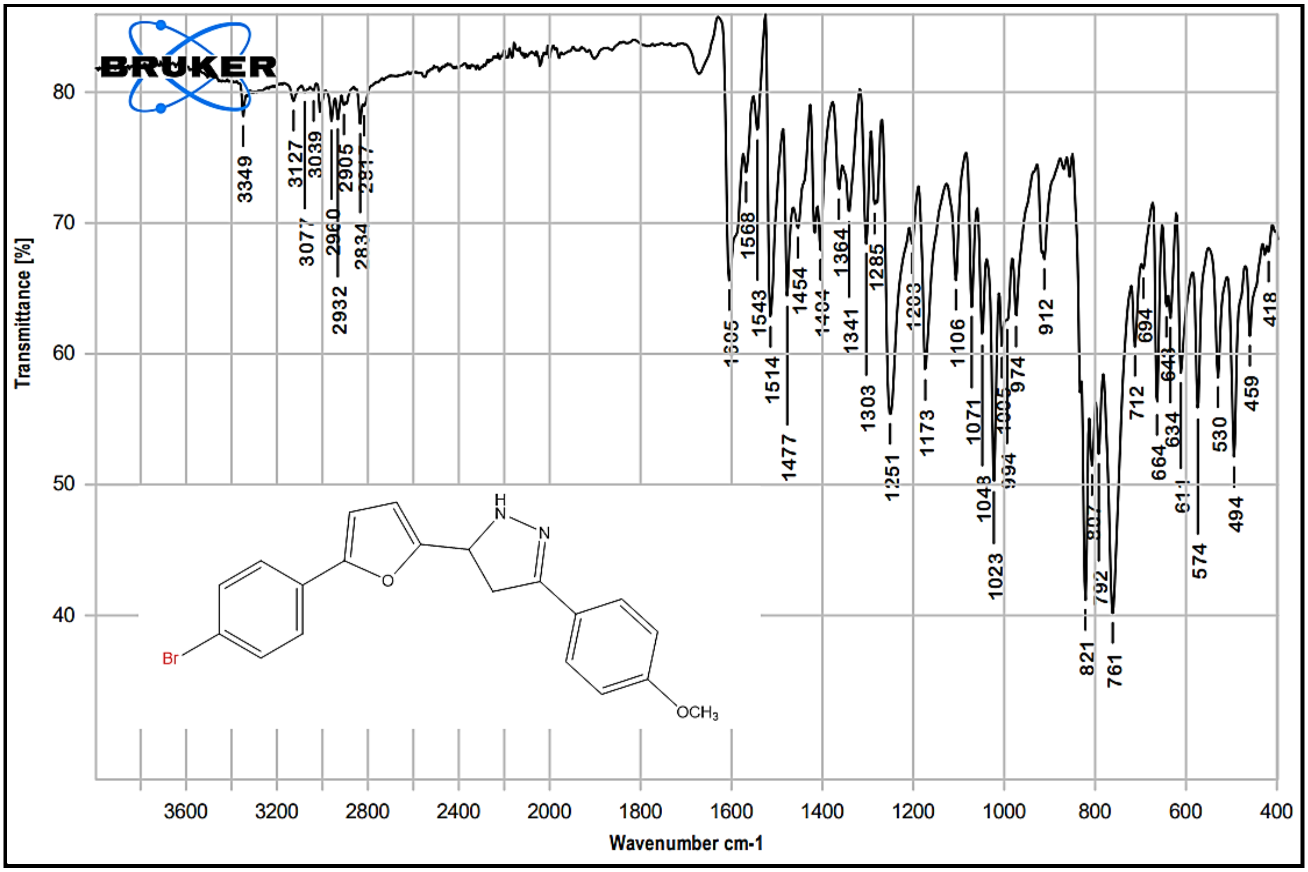

FTIR spectrum of compound 3b

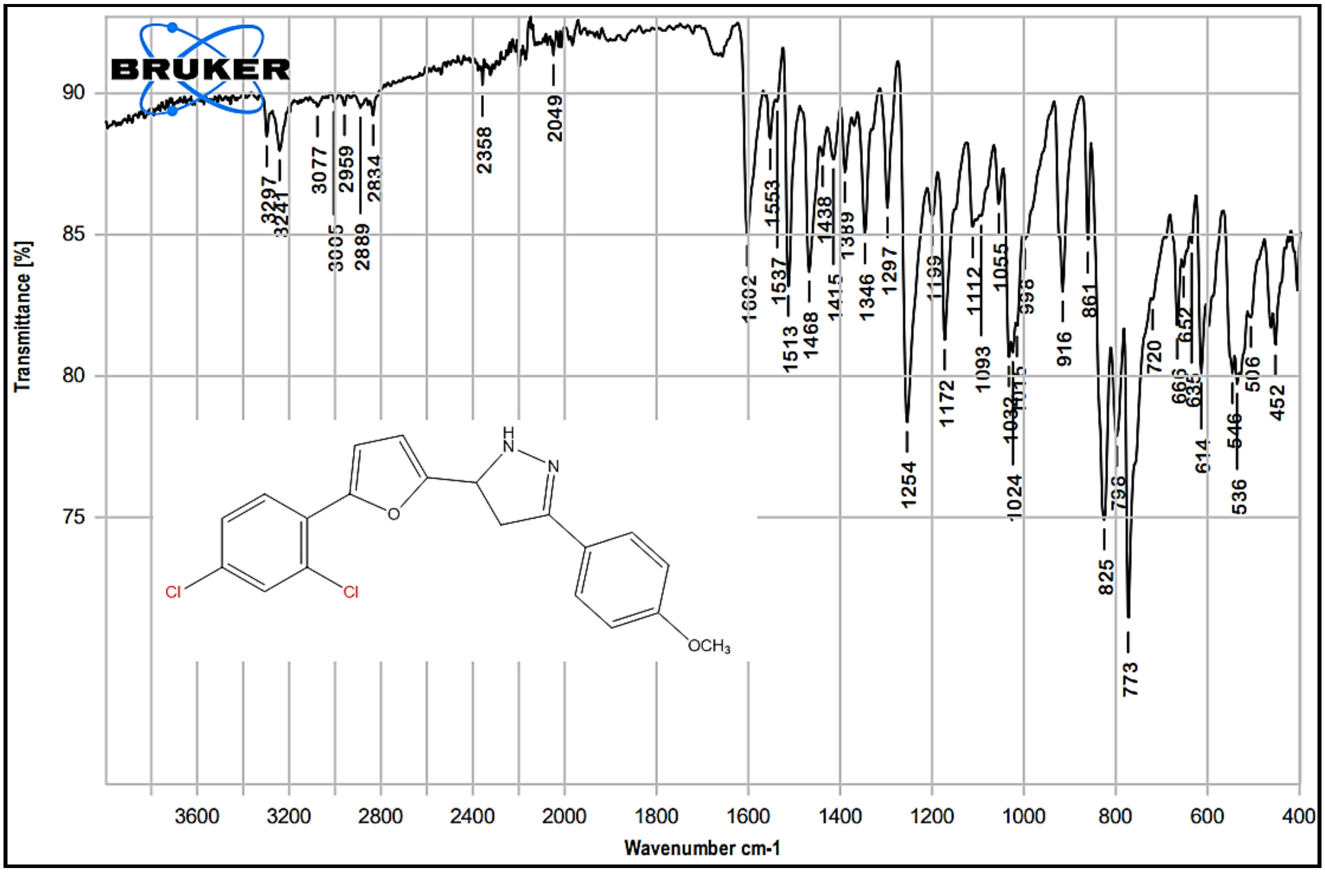

FTIR spectrum of compound 3c

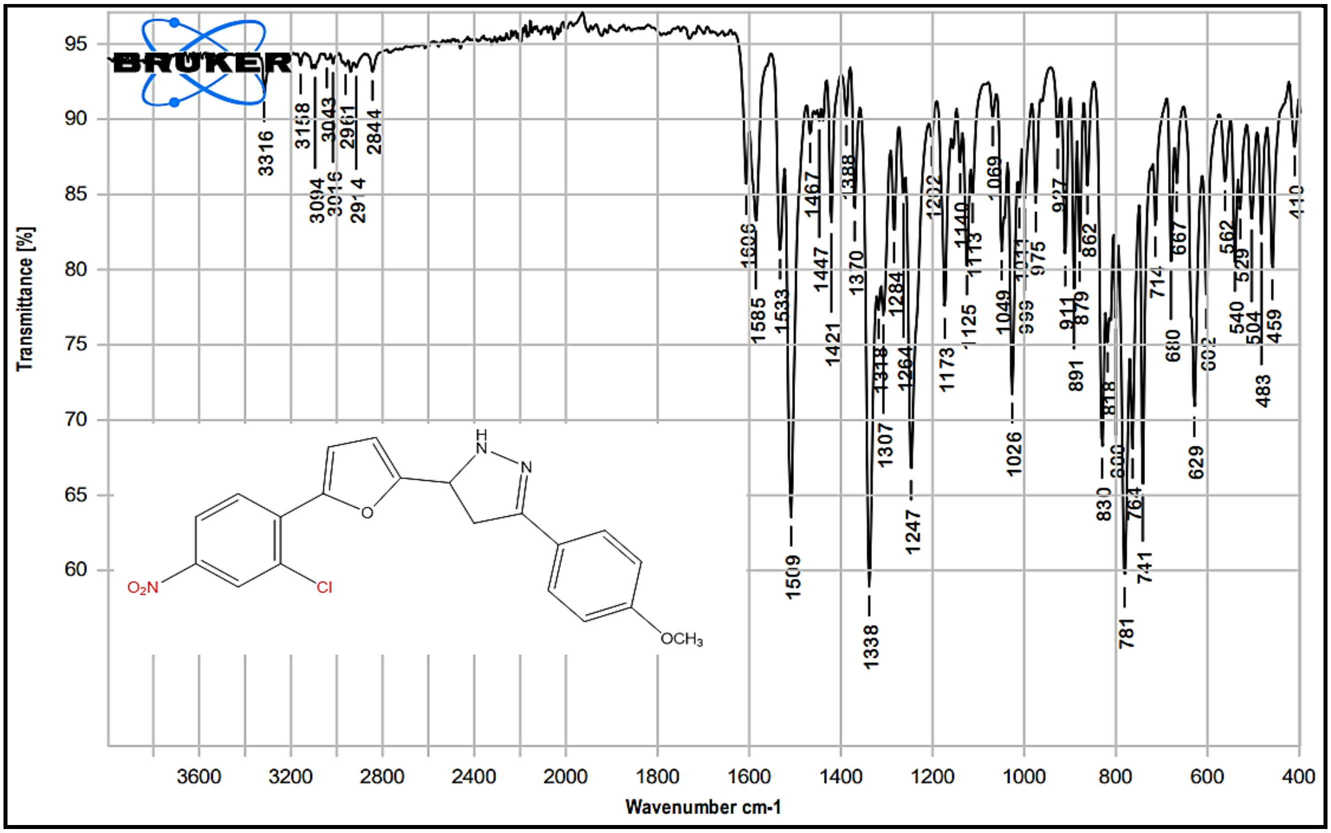

FTIR spectrum of compound 3d

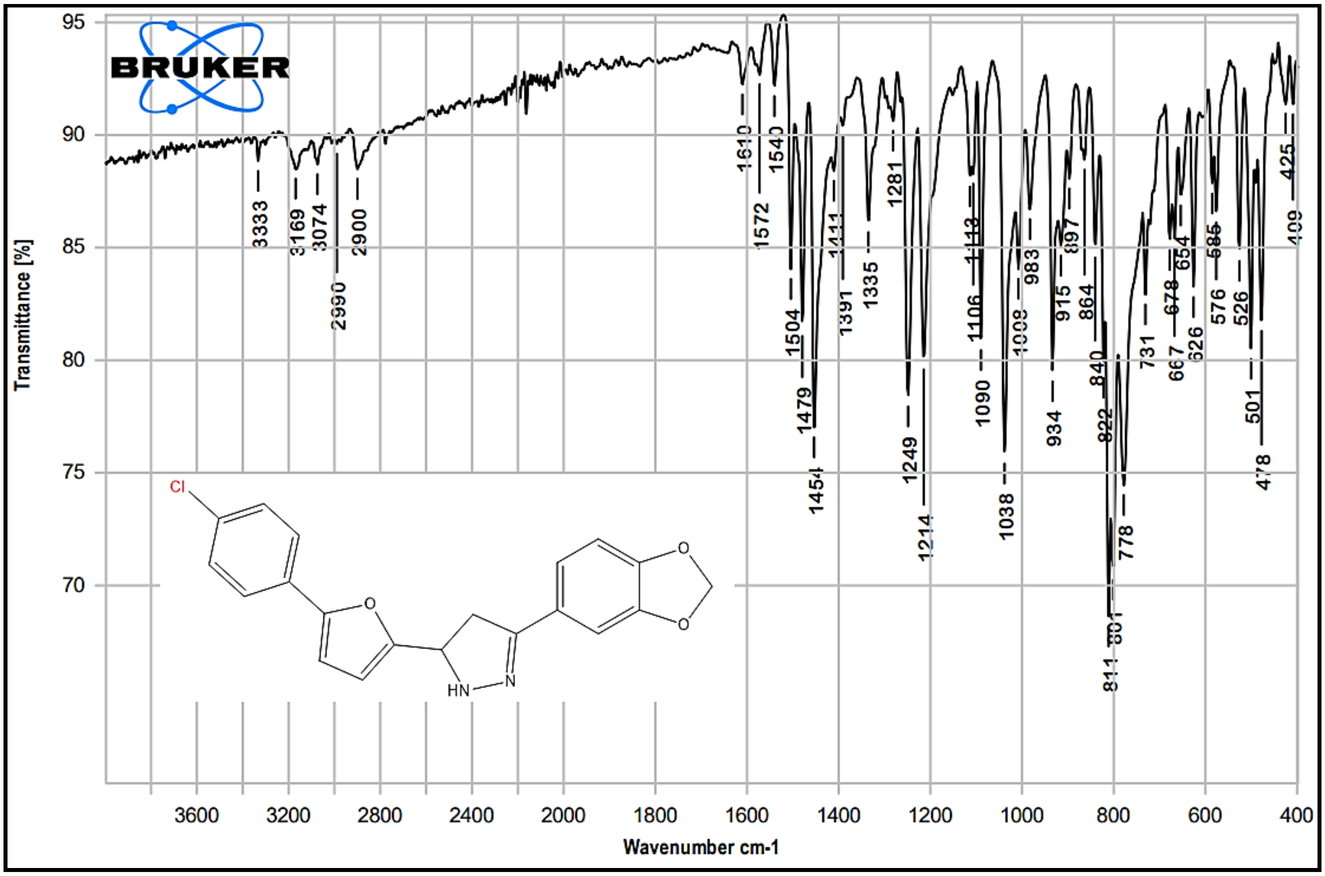

FTIR spectrum of compound 3e

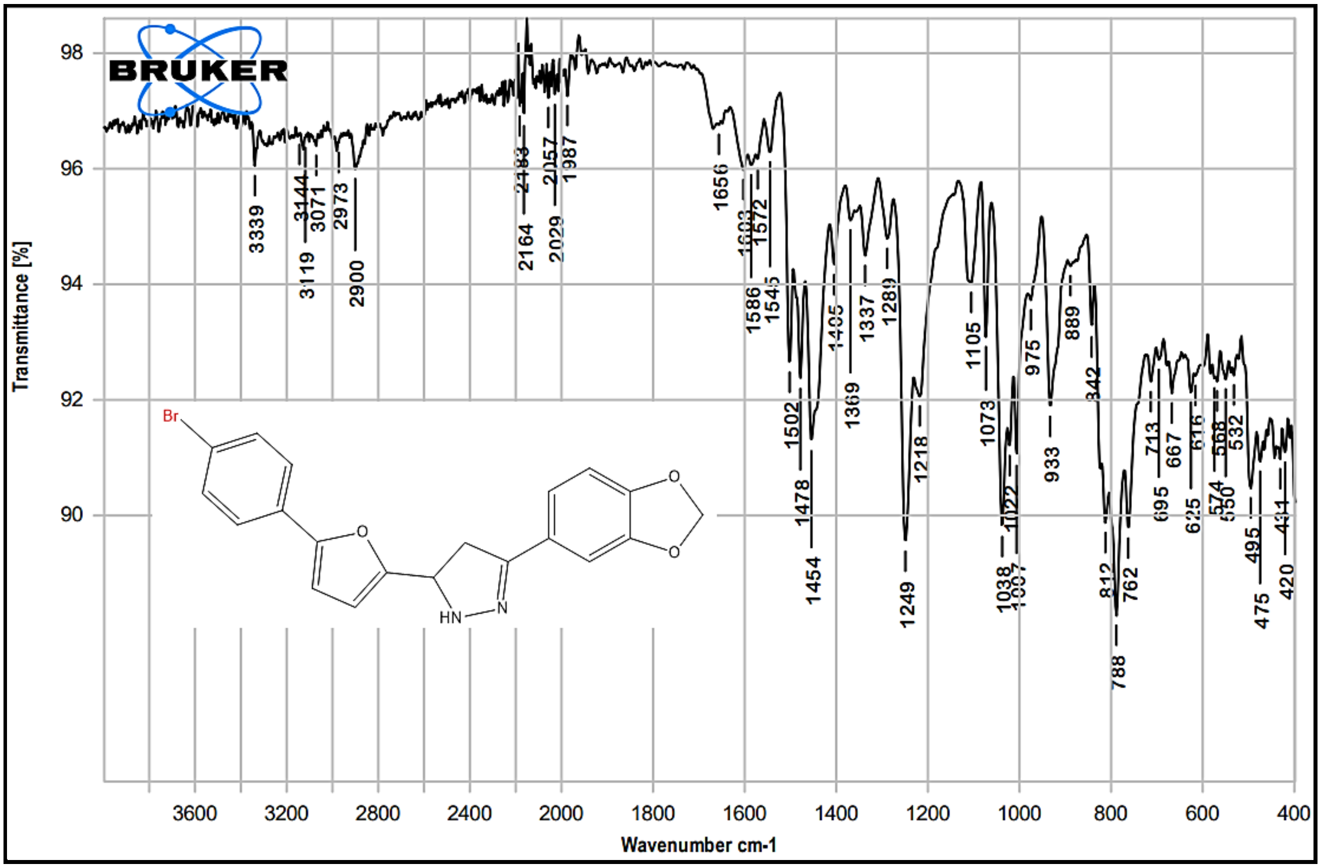

FTIR spectrum of compound 3f

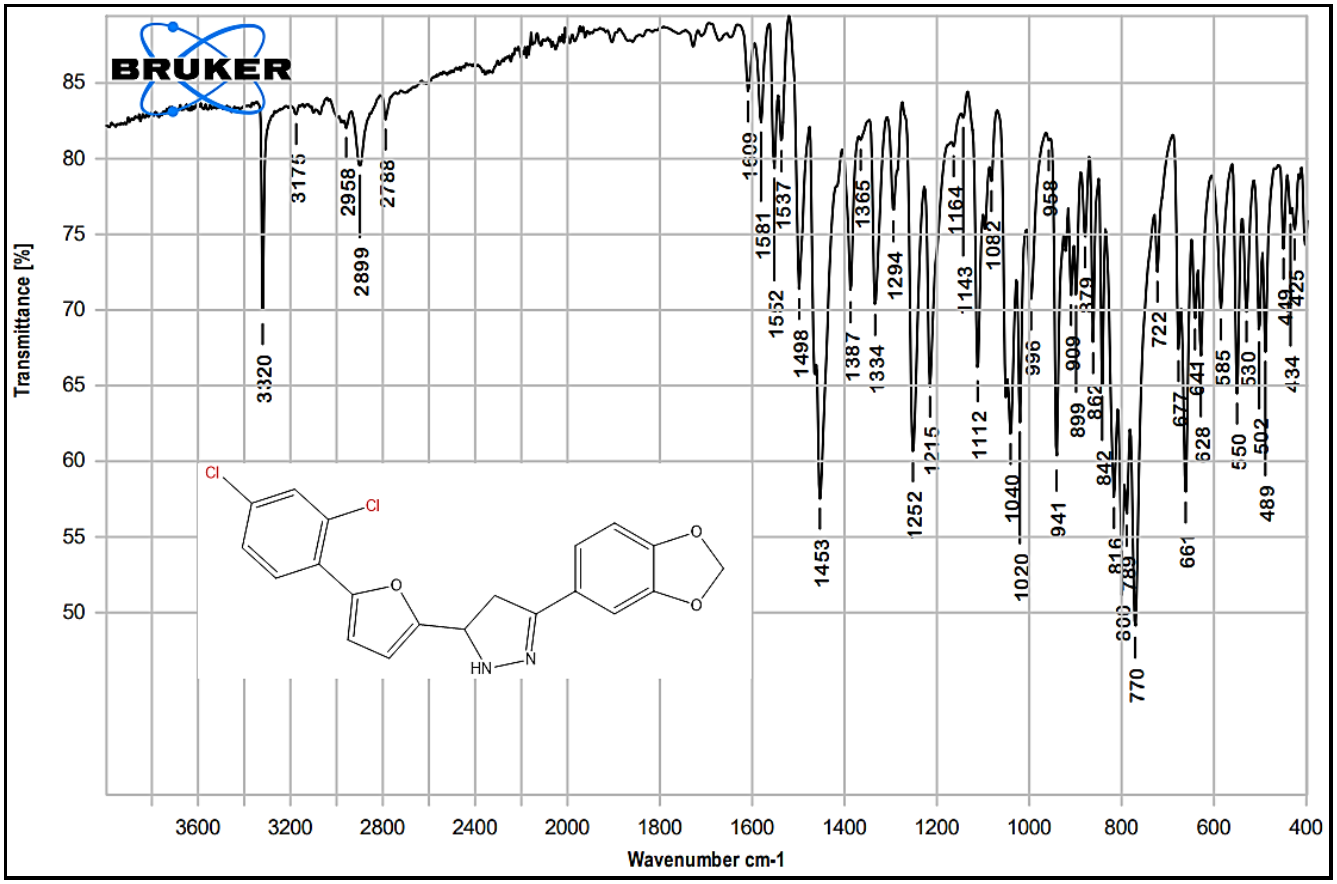

FTIR spectrum of compound 3g

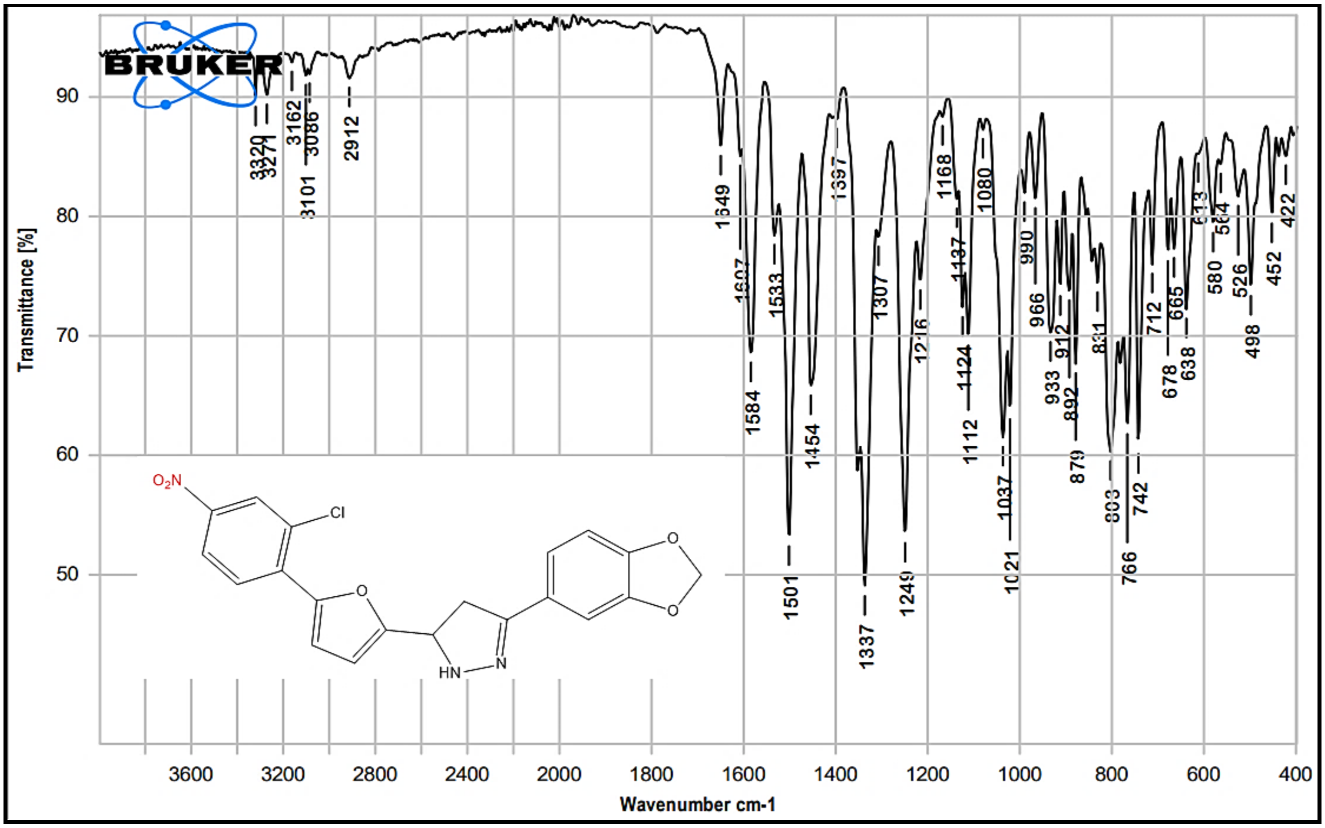

FTIR spectrum of compound 3h

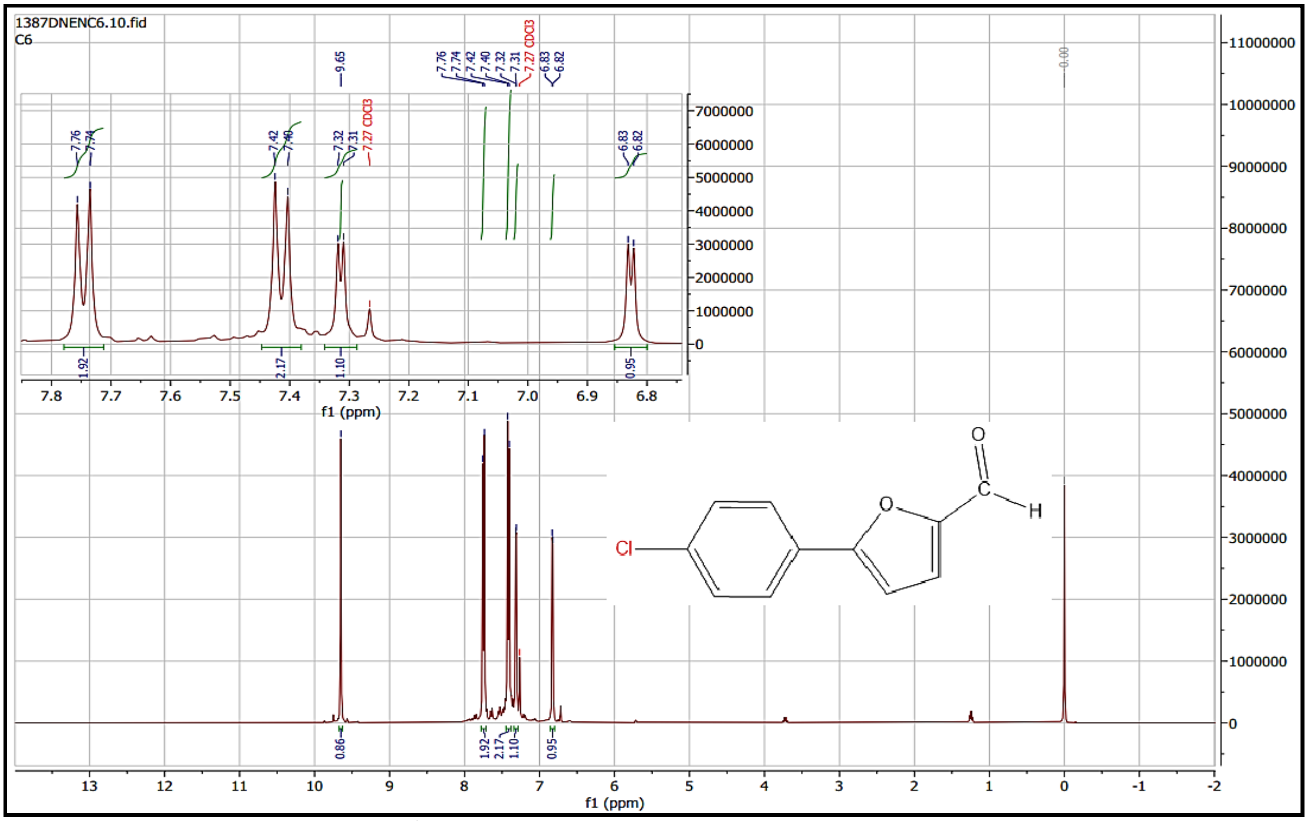

^{1}H-NMR spectrum of compound 1a

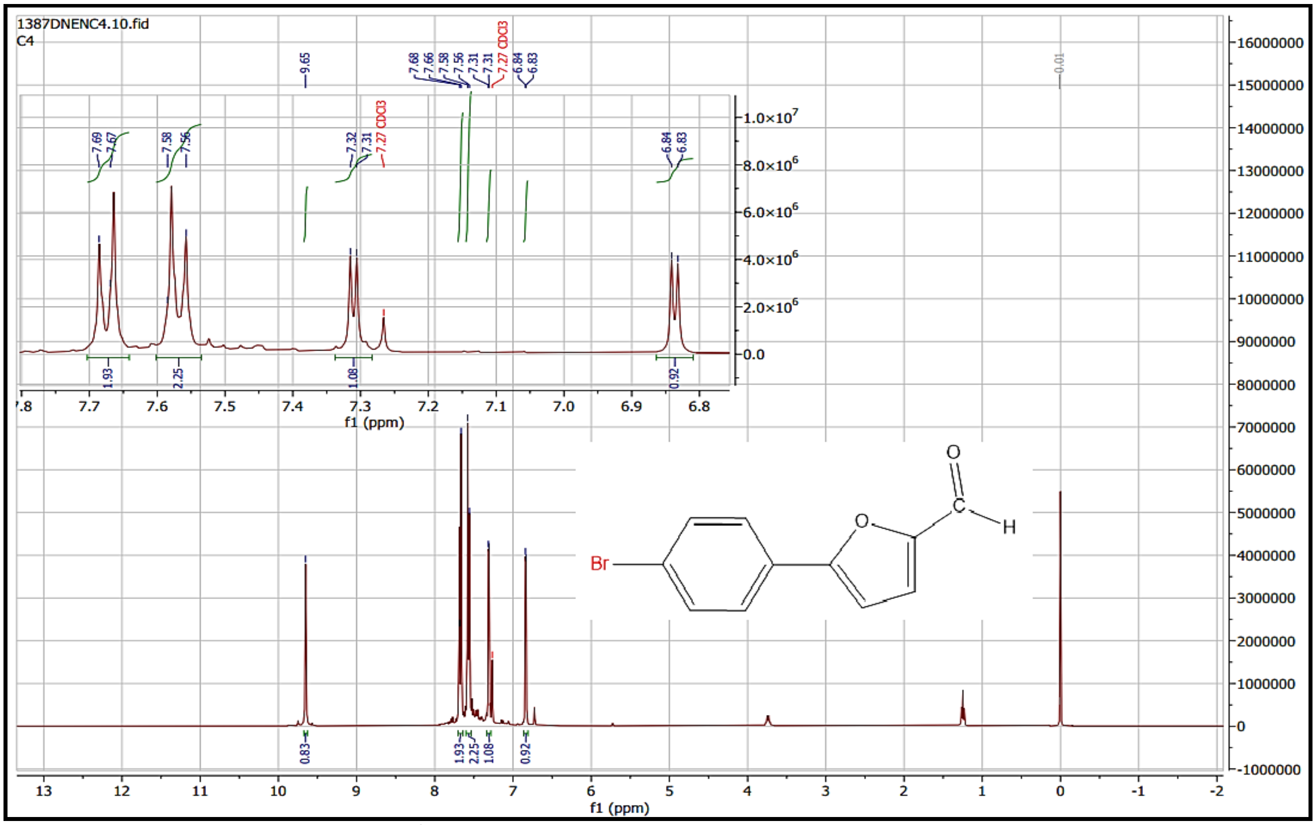

^{1}H-NMR spectrum of compound 1b

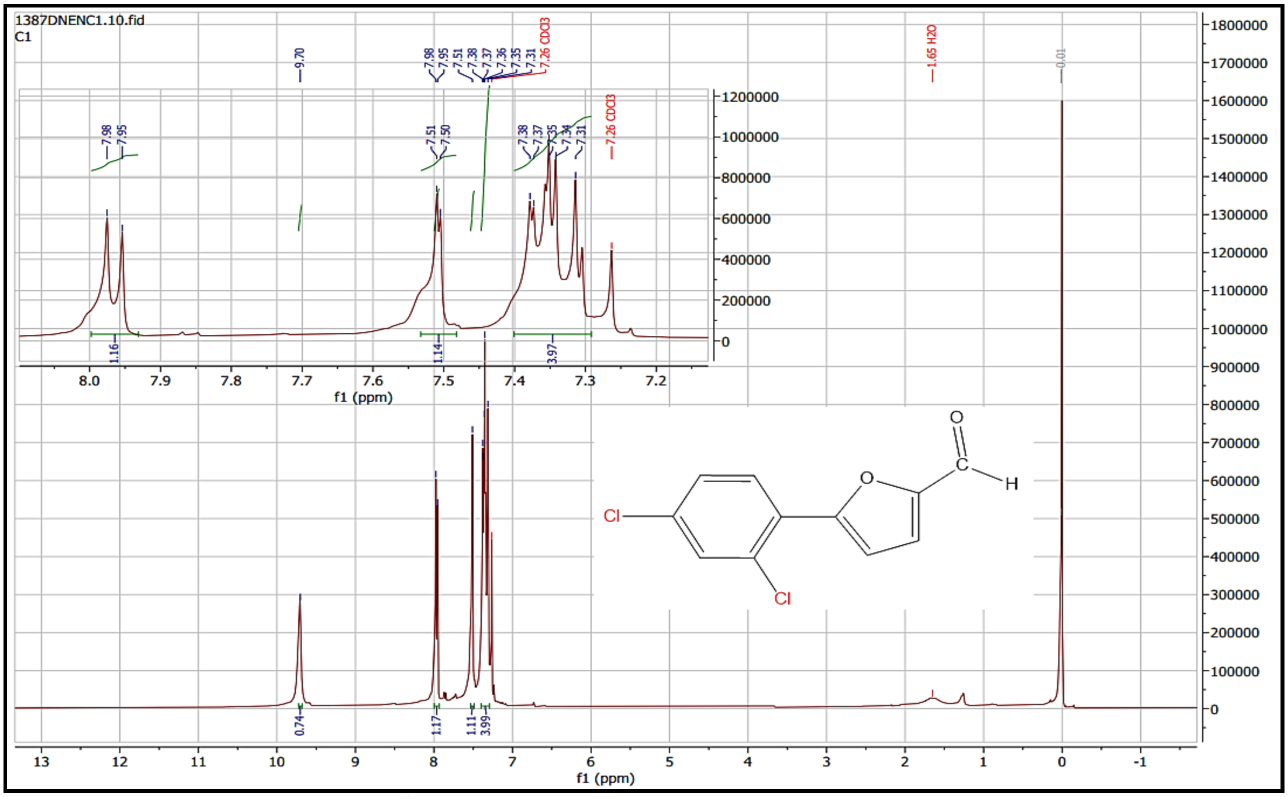

^{1}H-NMR spectrum of compound 1c

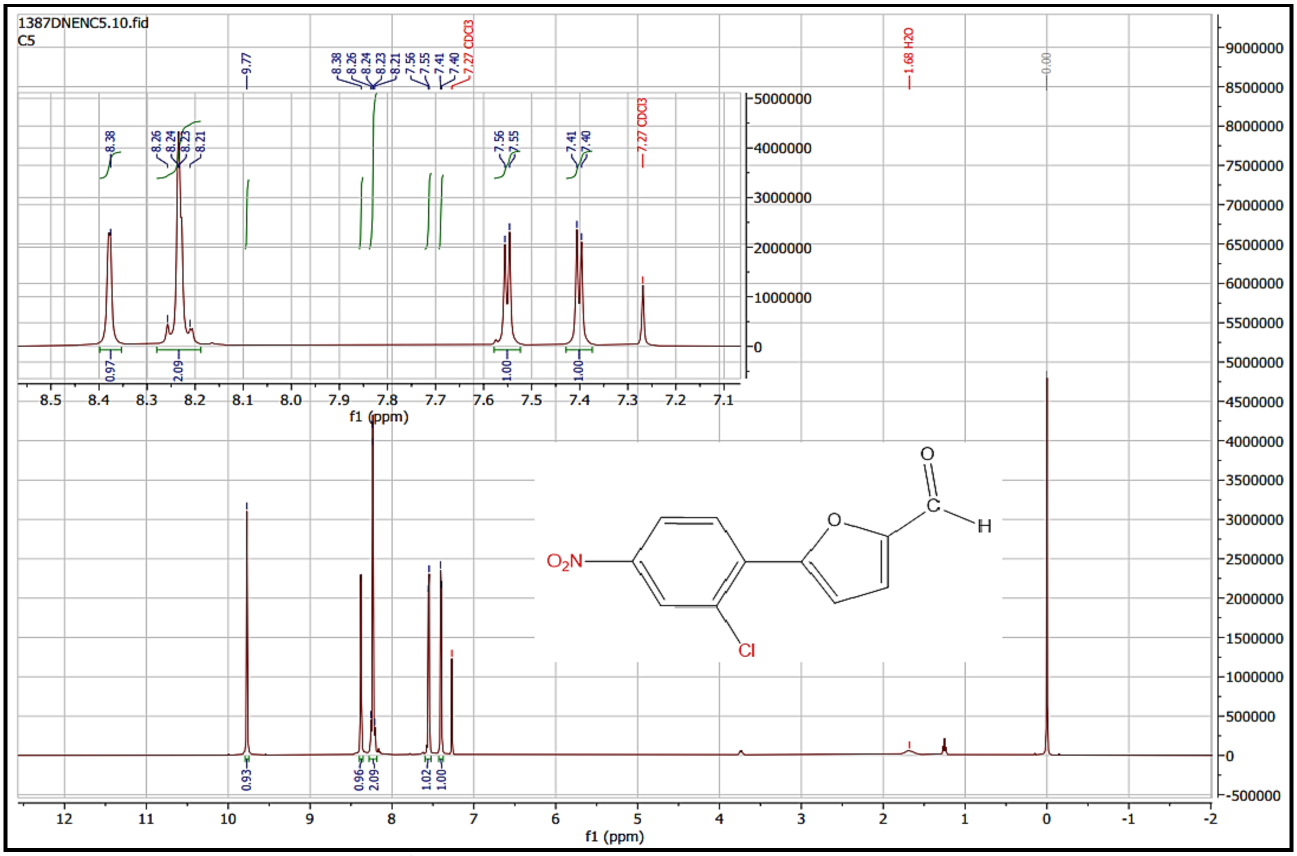

^{1}H-NMR spectrum of compound 1d

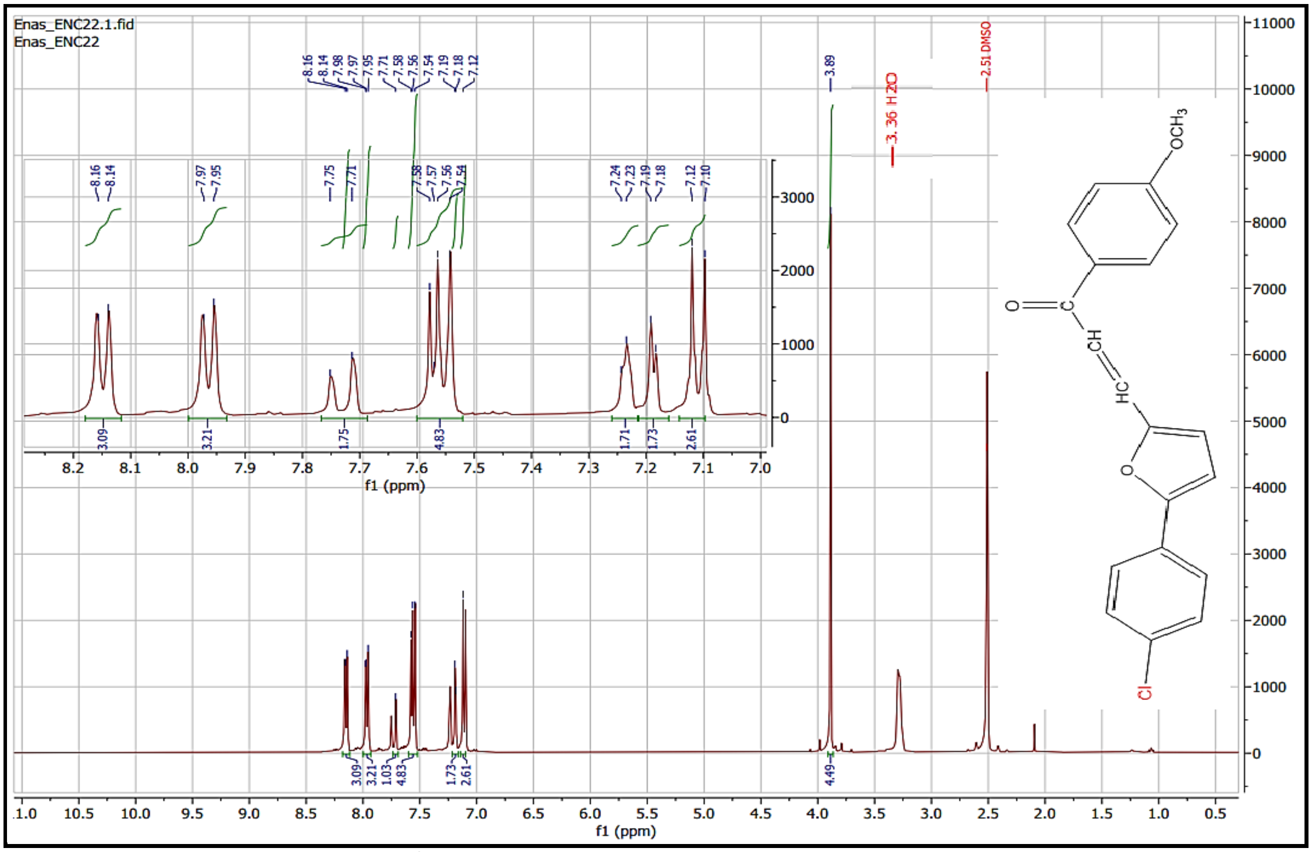

^{1}H-NMR spectrum of compound 2a

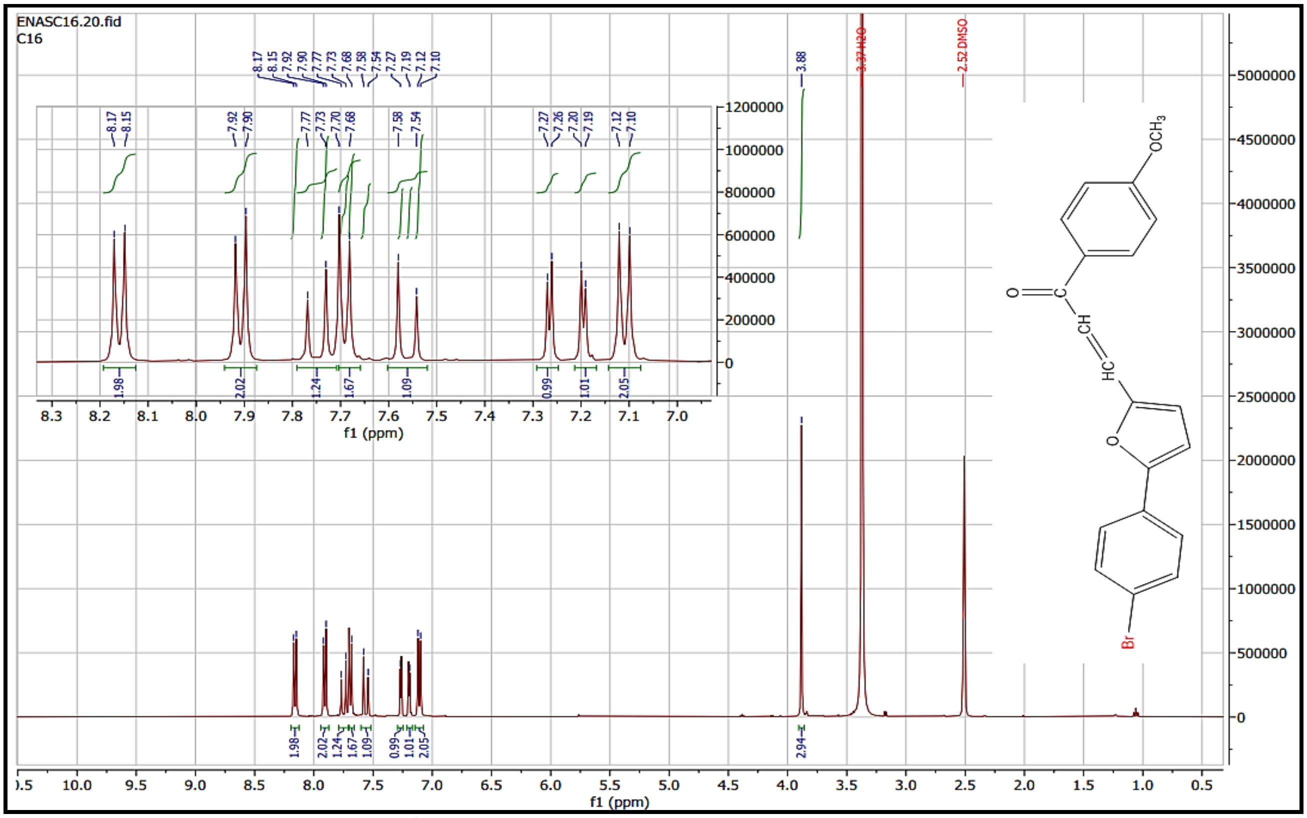

^{1}H-NMR spectrum of compound 2b

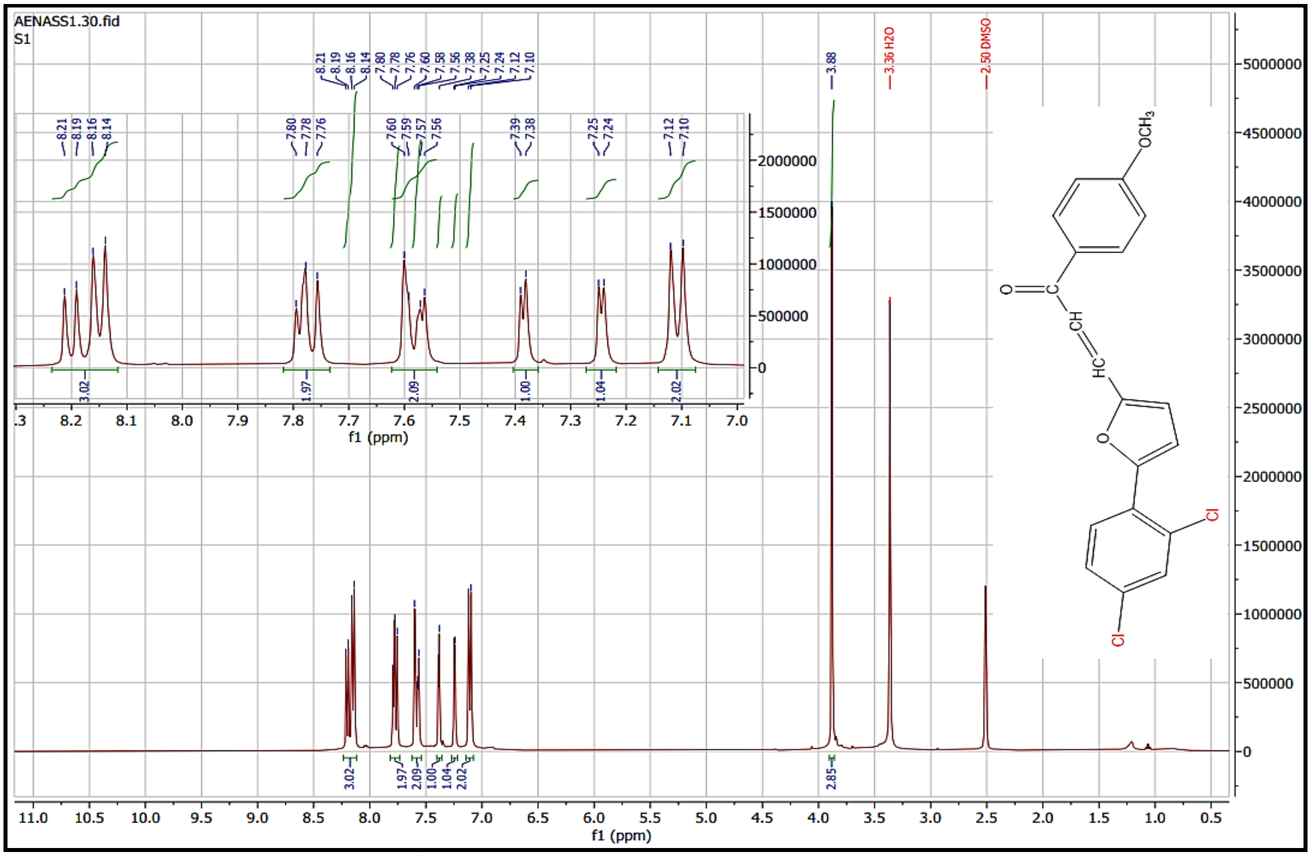

^{1}H-NMR spectrum of compound 2c

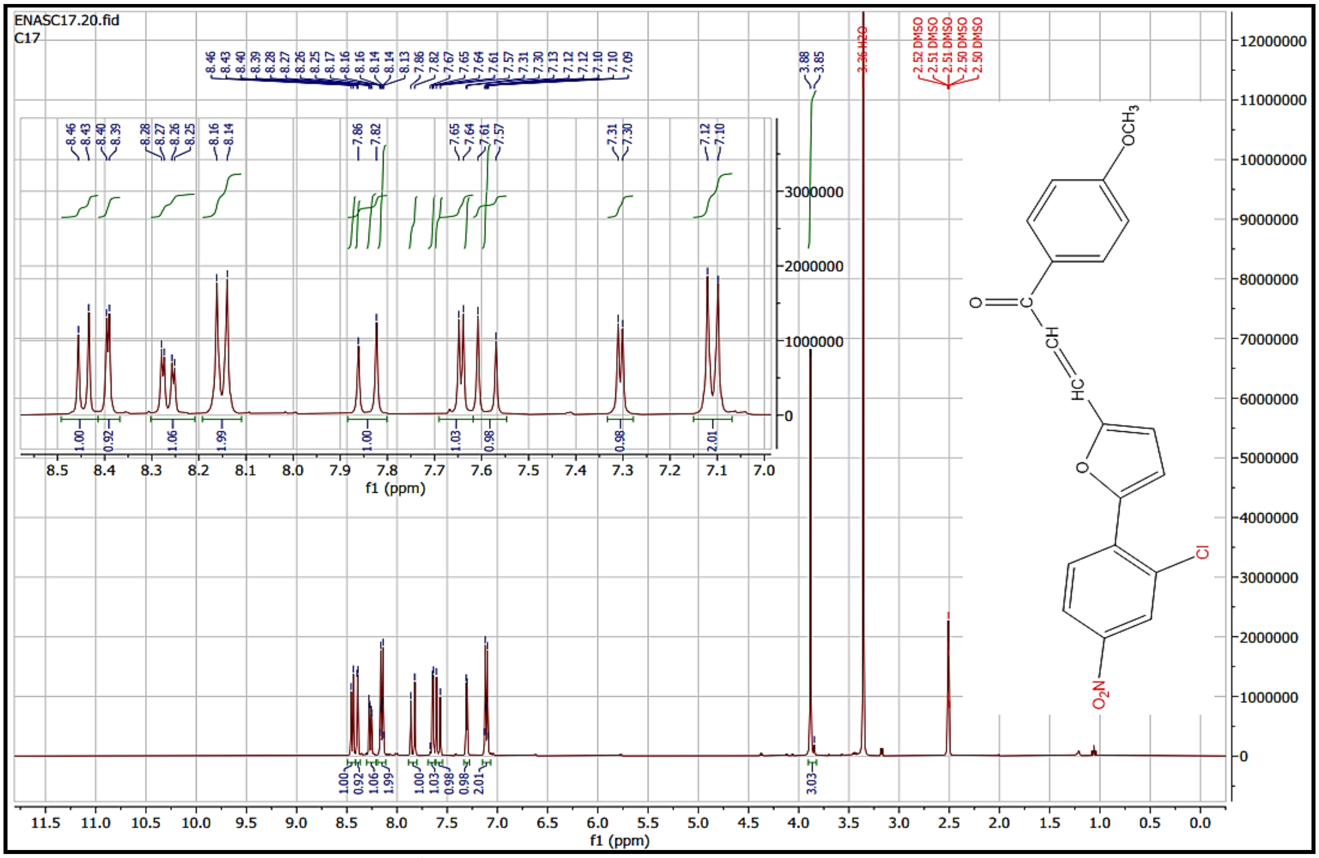

^{1}H-NMR spectrum of compound 2d

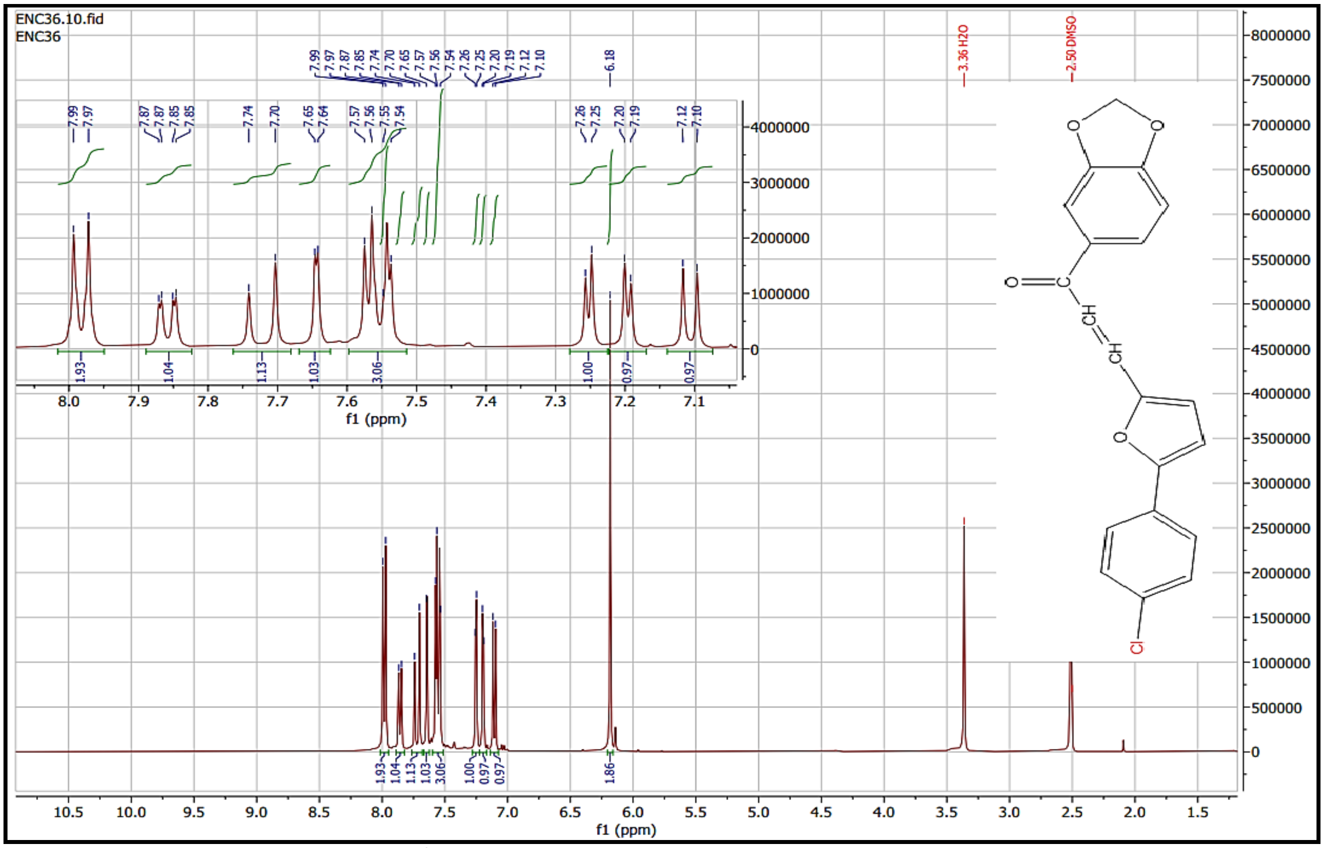

^{1}H-NMR spectrum of compound 2e

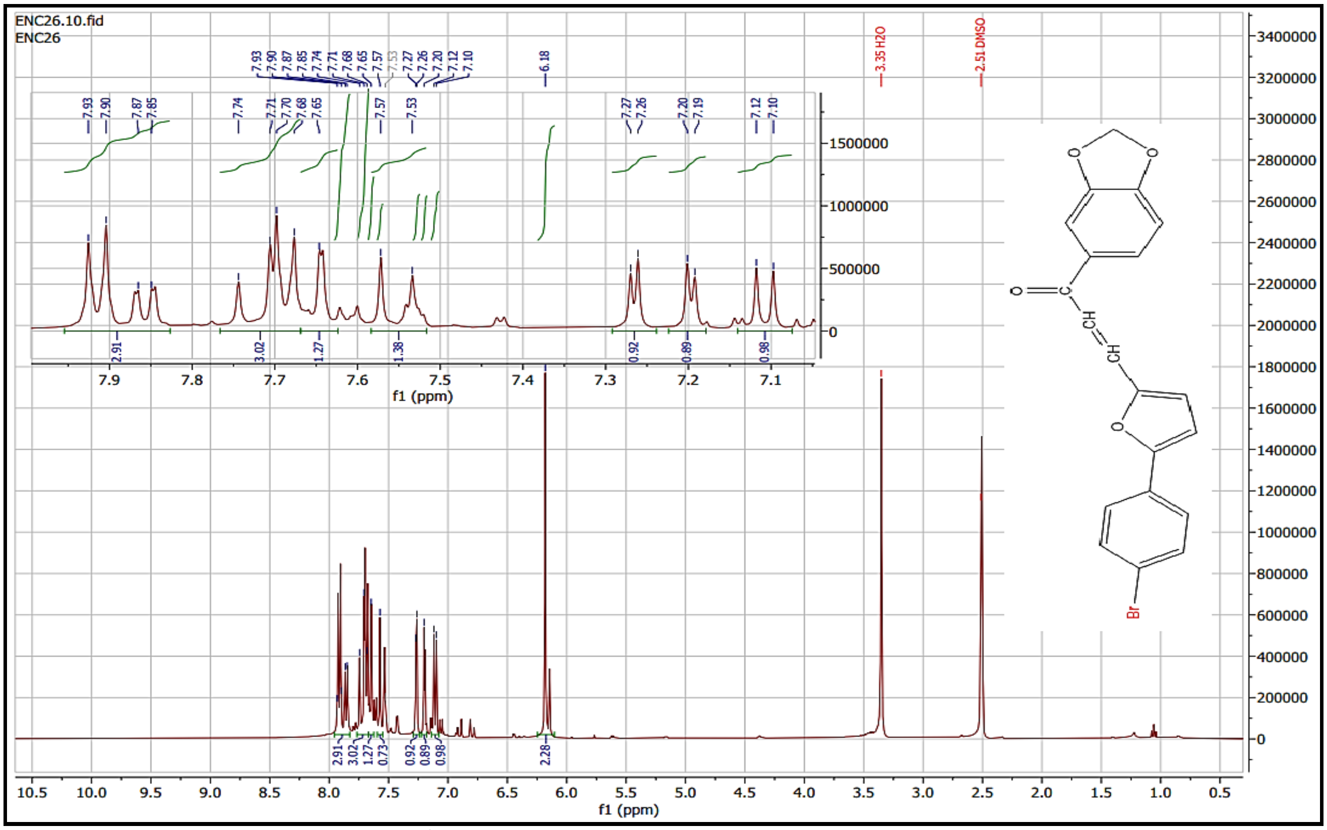

^{1}H-NMR spectrum of compound 2f

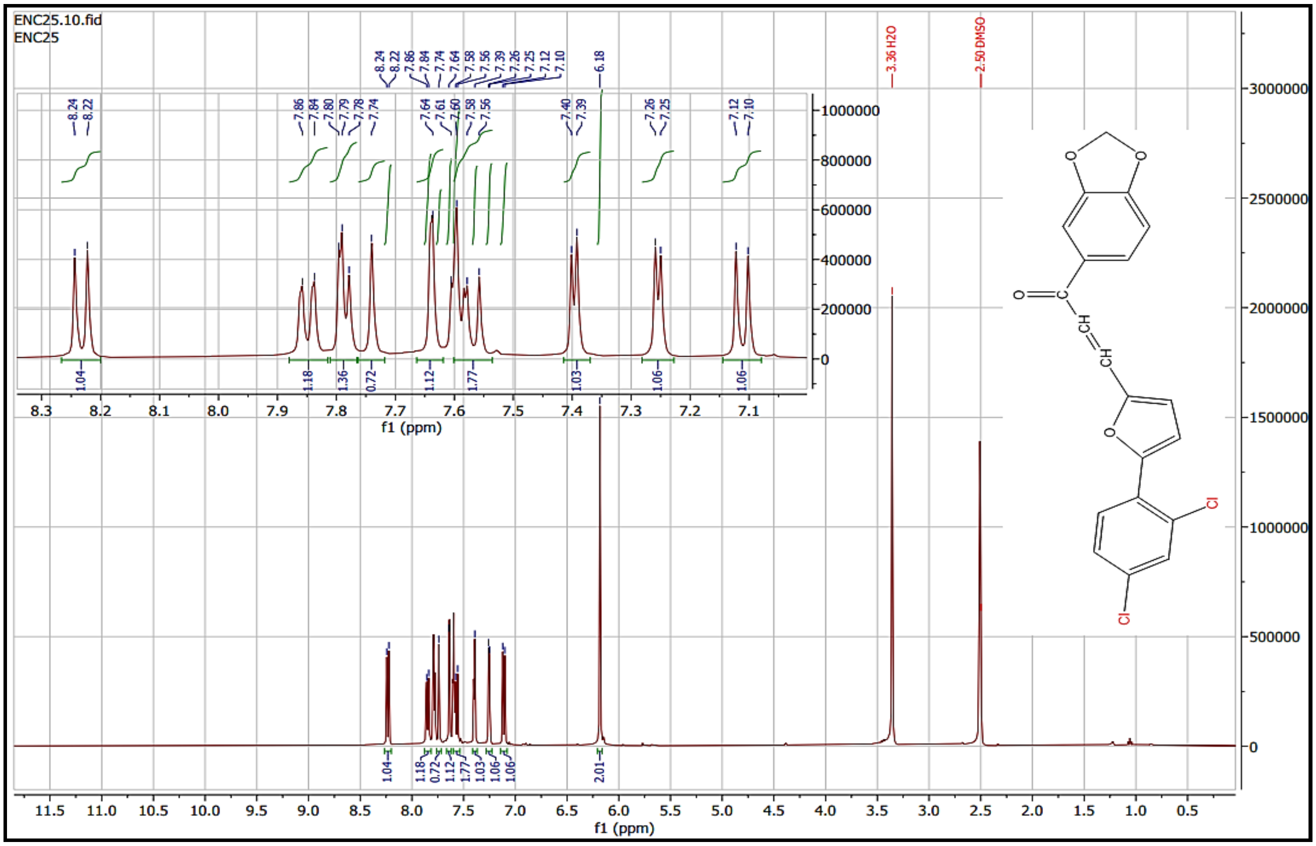

^{1}H-NMR spectrum of compound 2g

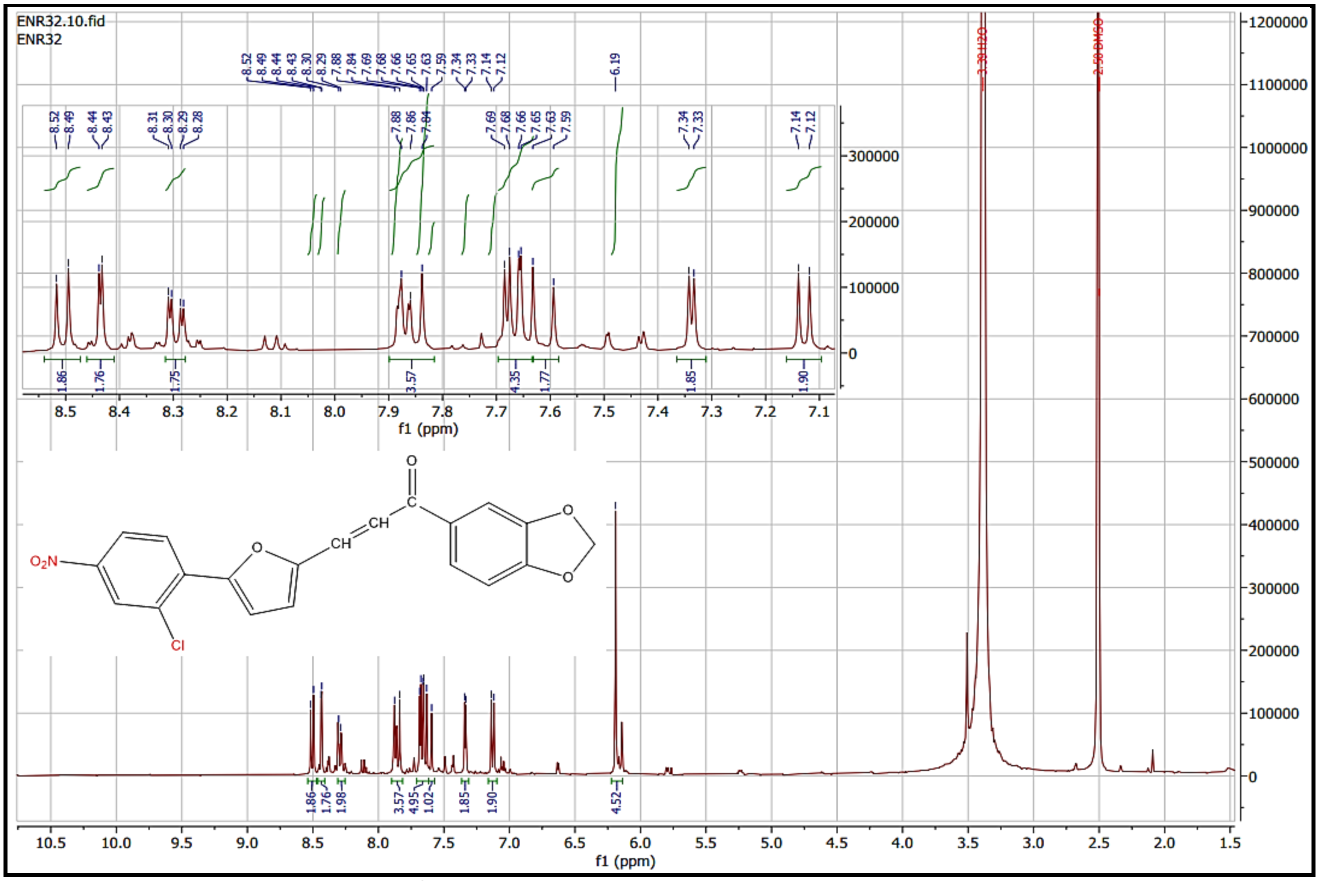

^{1}H-NMR spectrum of compound 2h

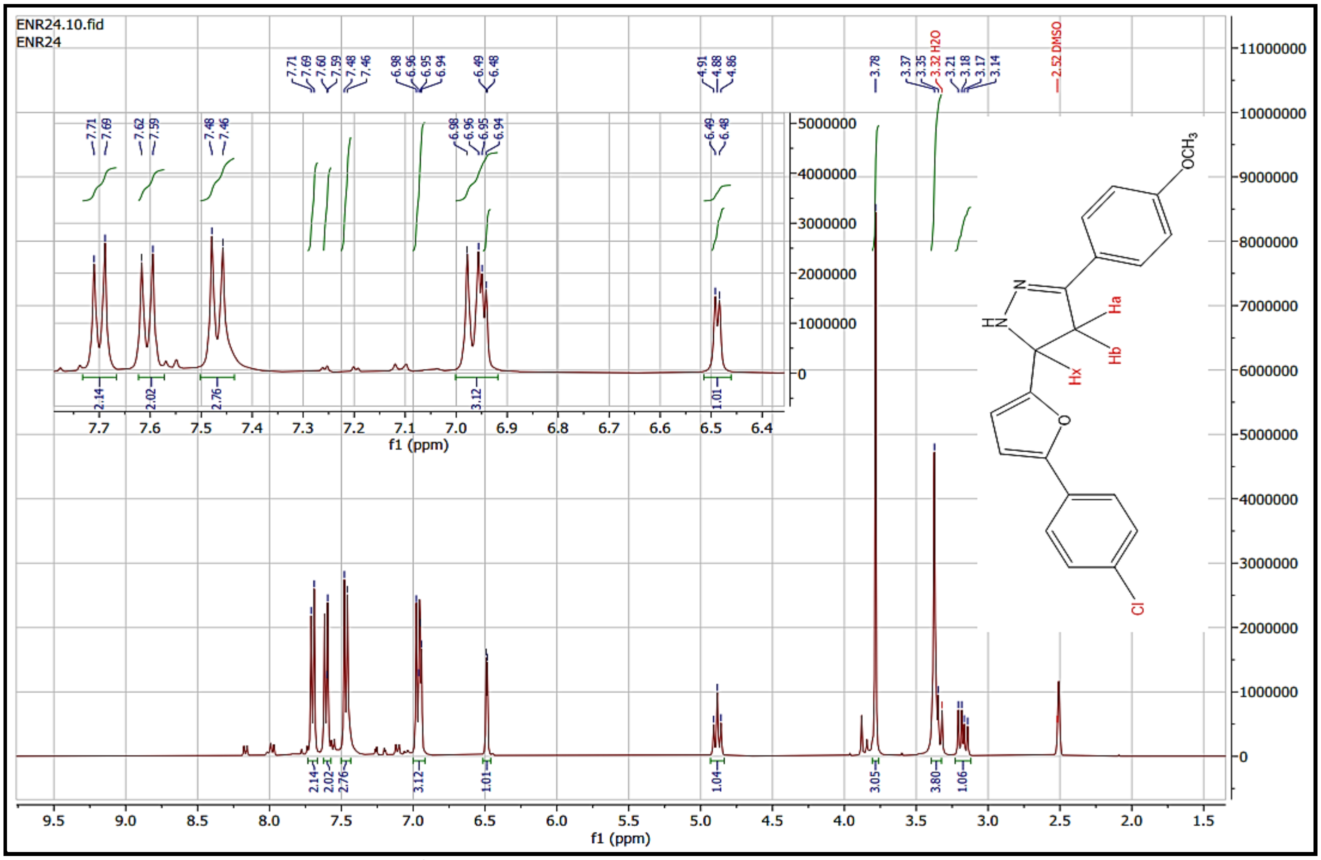

^{1}H-NMR spectrum of compound 3a

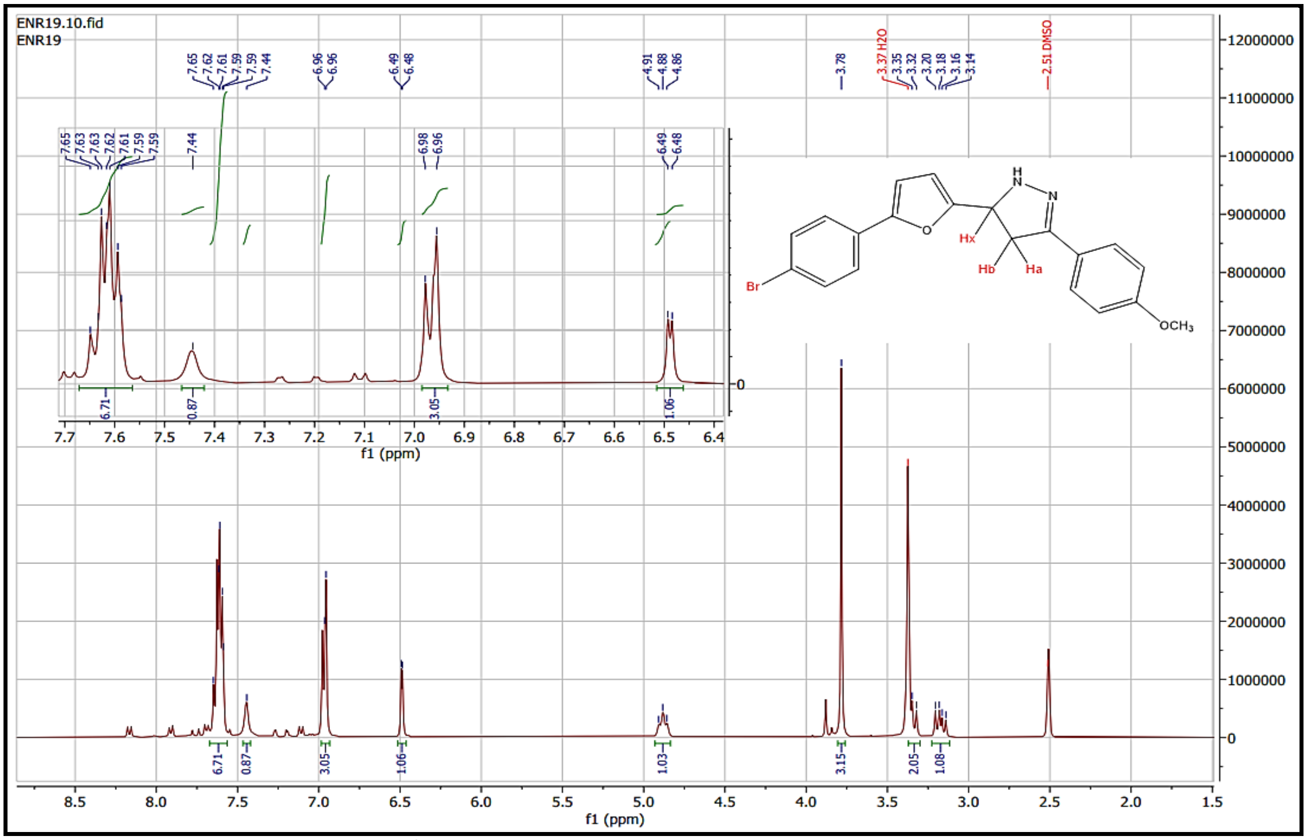

^{1}H-NMR spectrum of compound 3b

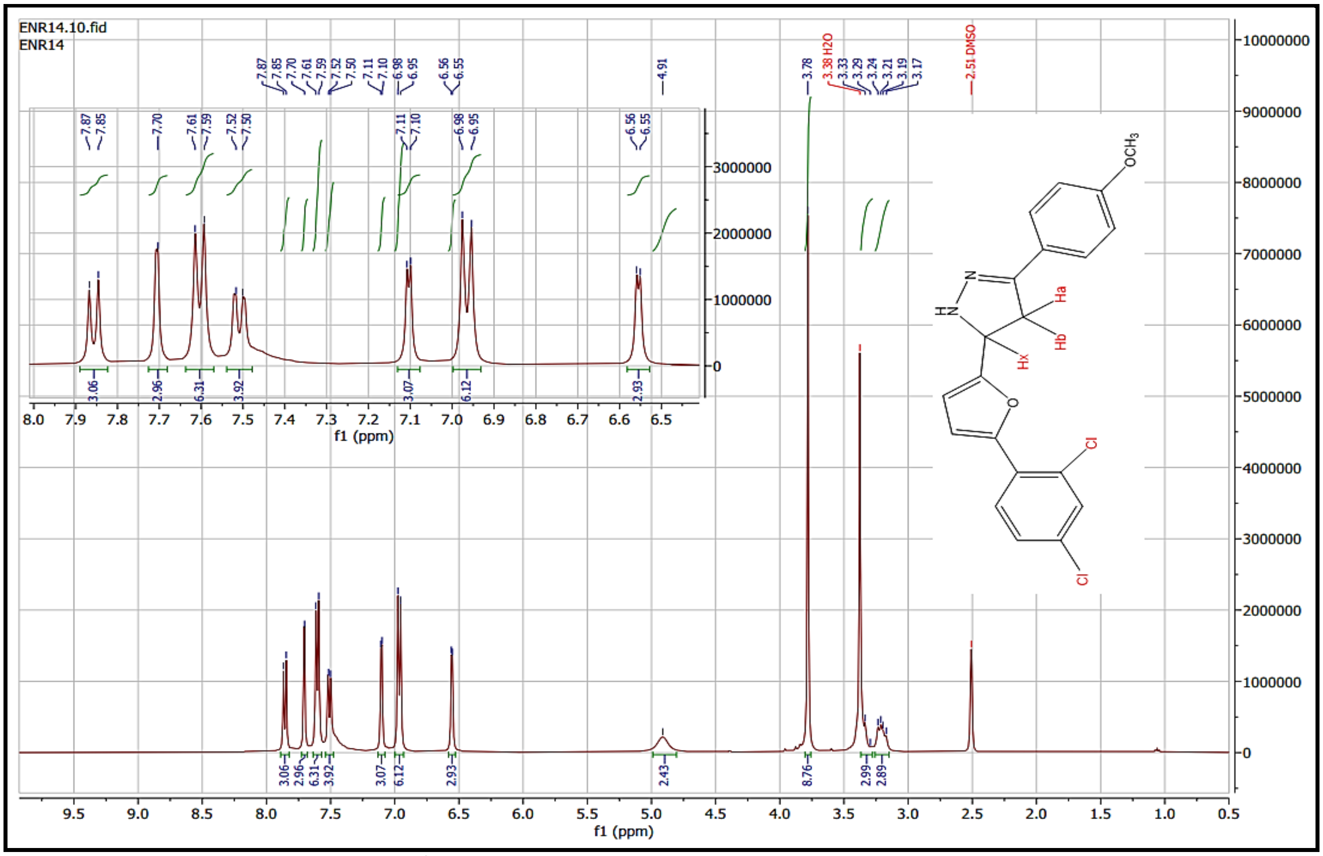

^{1}H-NMR spectrum of compound 3c

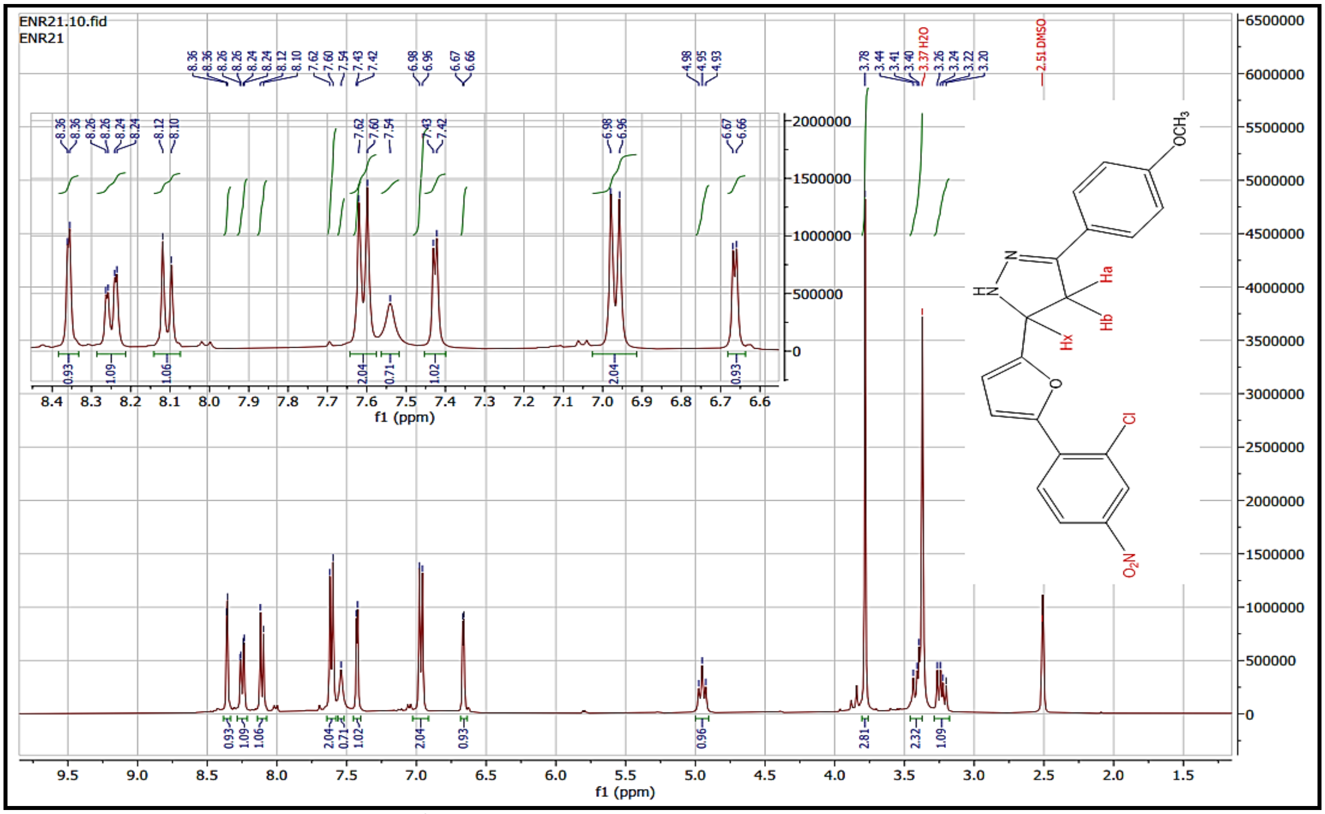

^{1}H-NMR spectrum of compound 3d

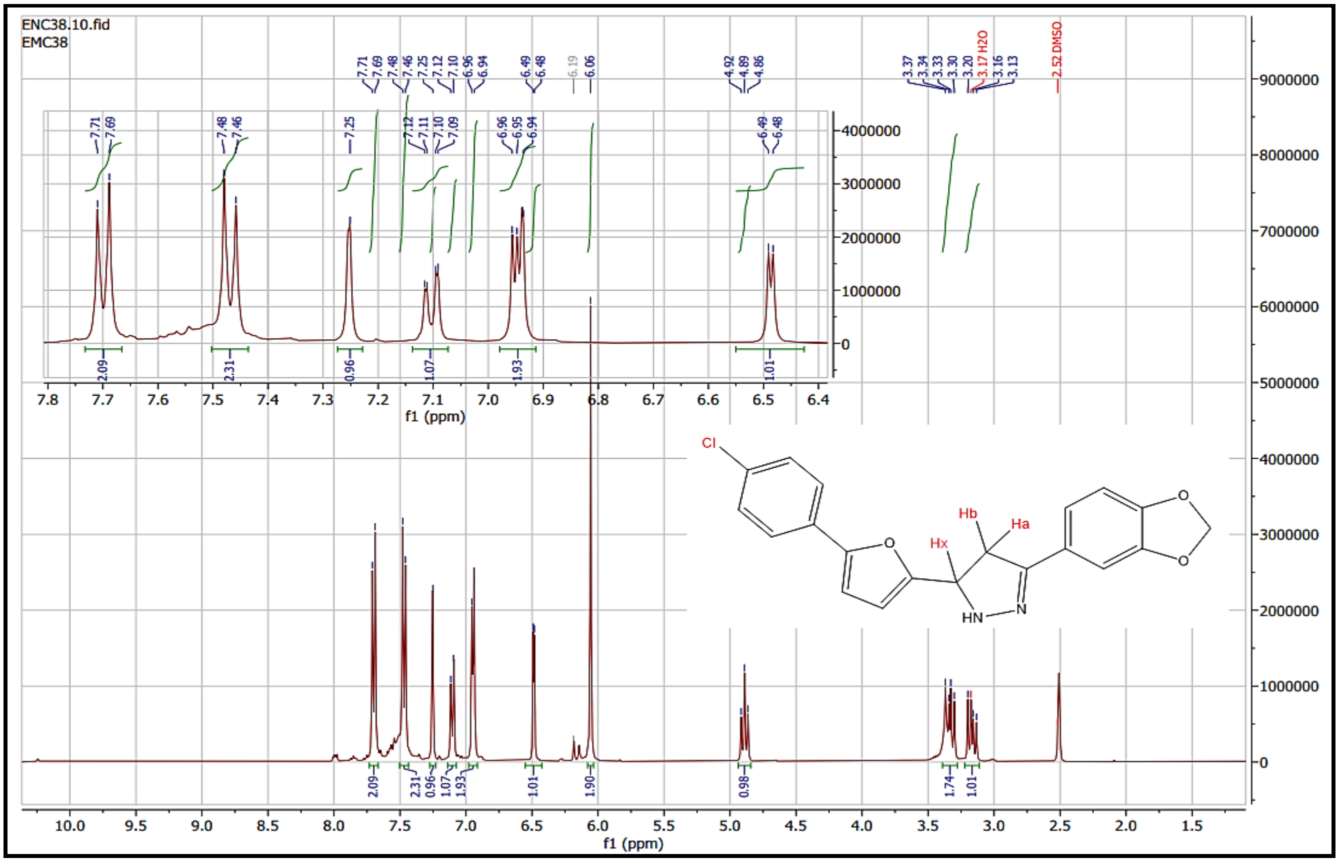

^{1}H-NMR spectrum of compound 3e

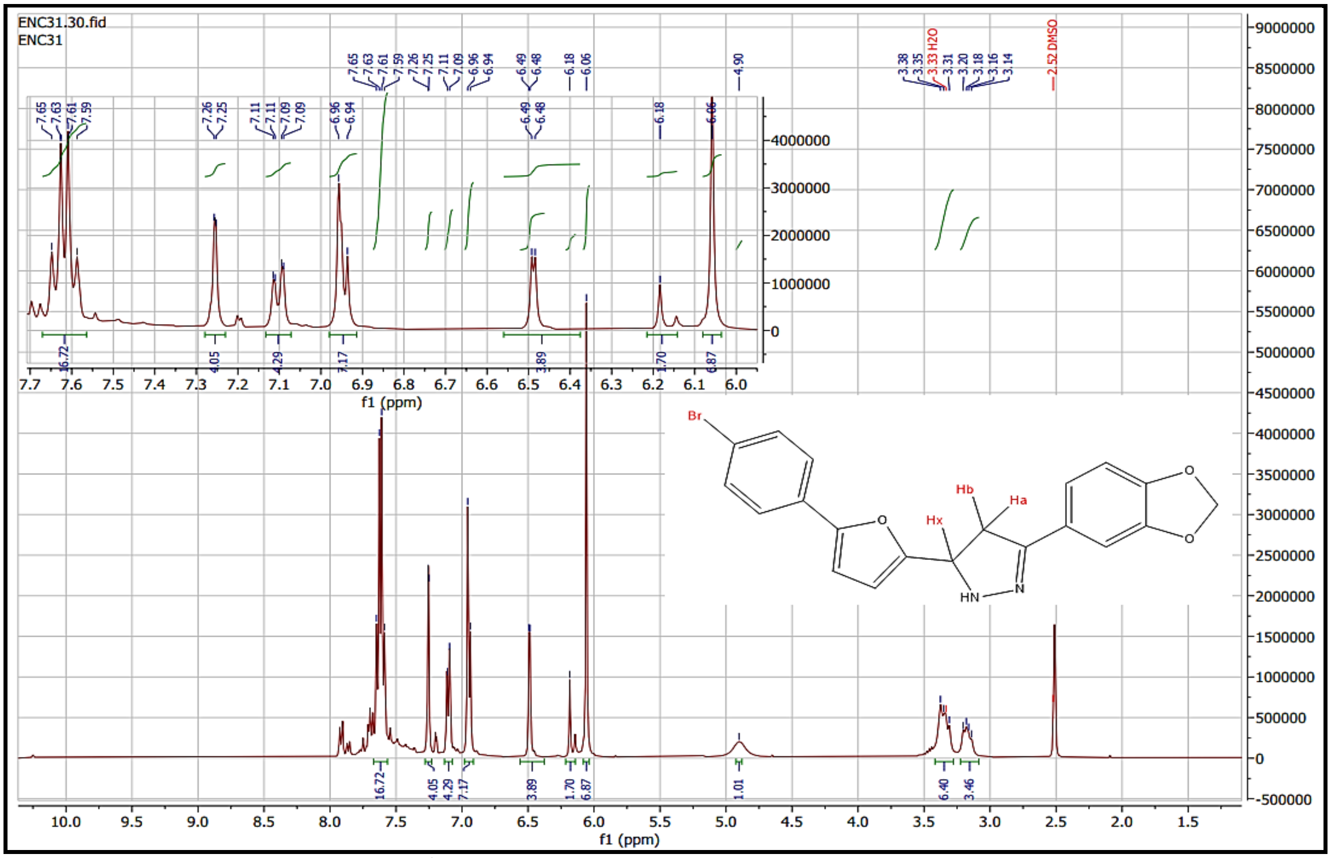

^{1}H-NMR spectrum of compound 3f

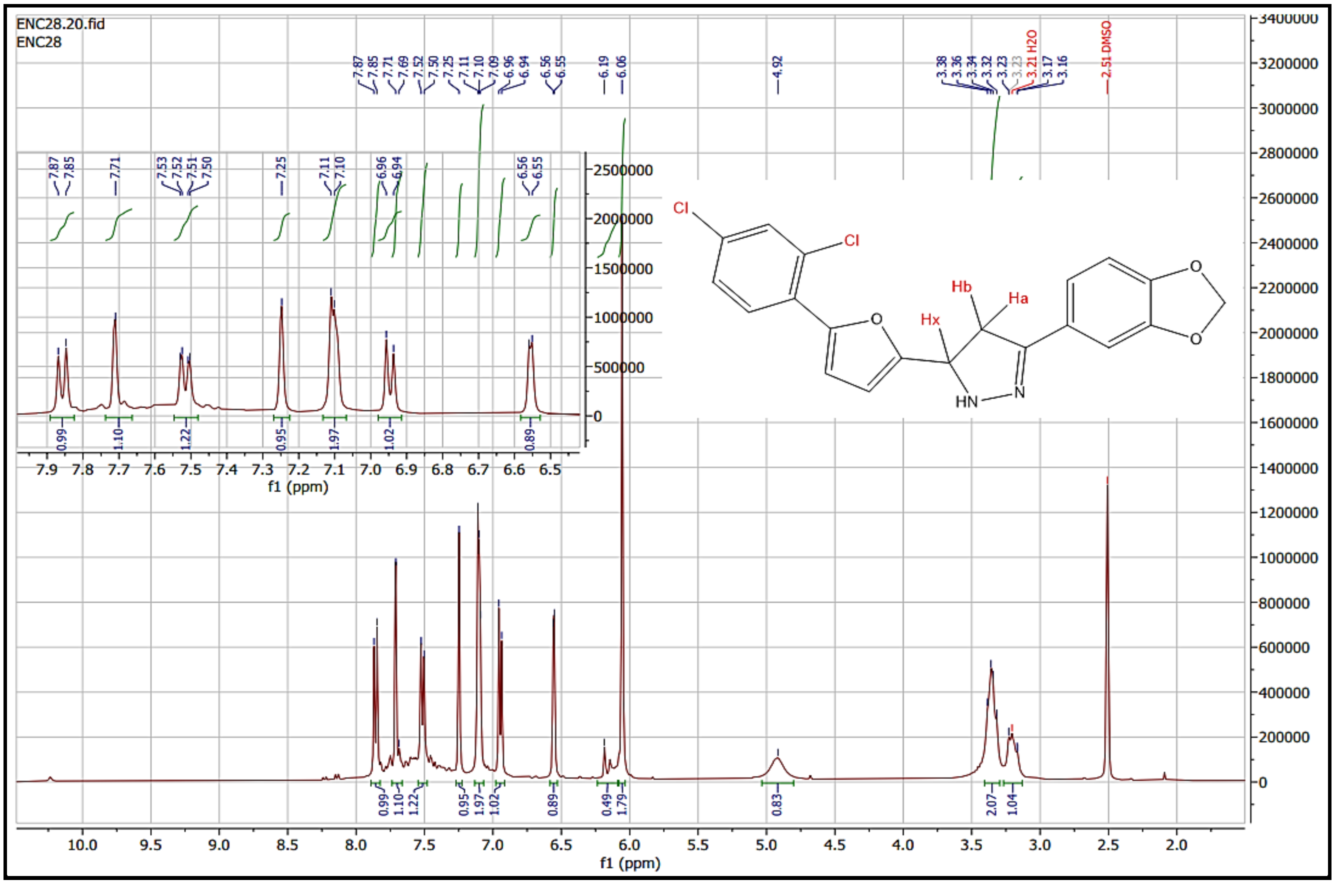

^{1}H-NMR spectrum of compound 3g

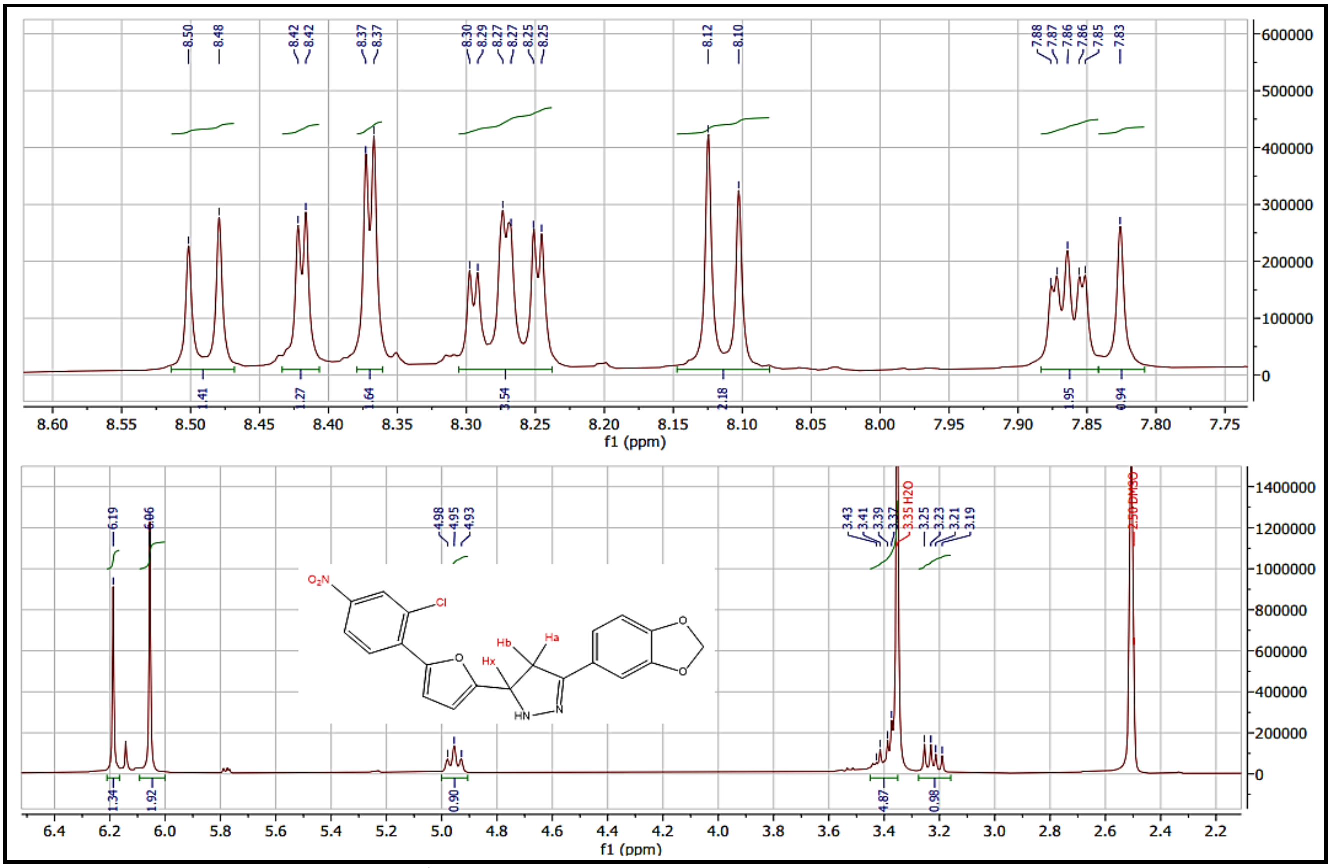

^{1}H-NMR spectrum of compound 3h

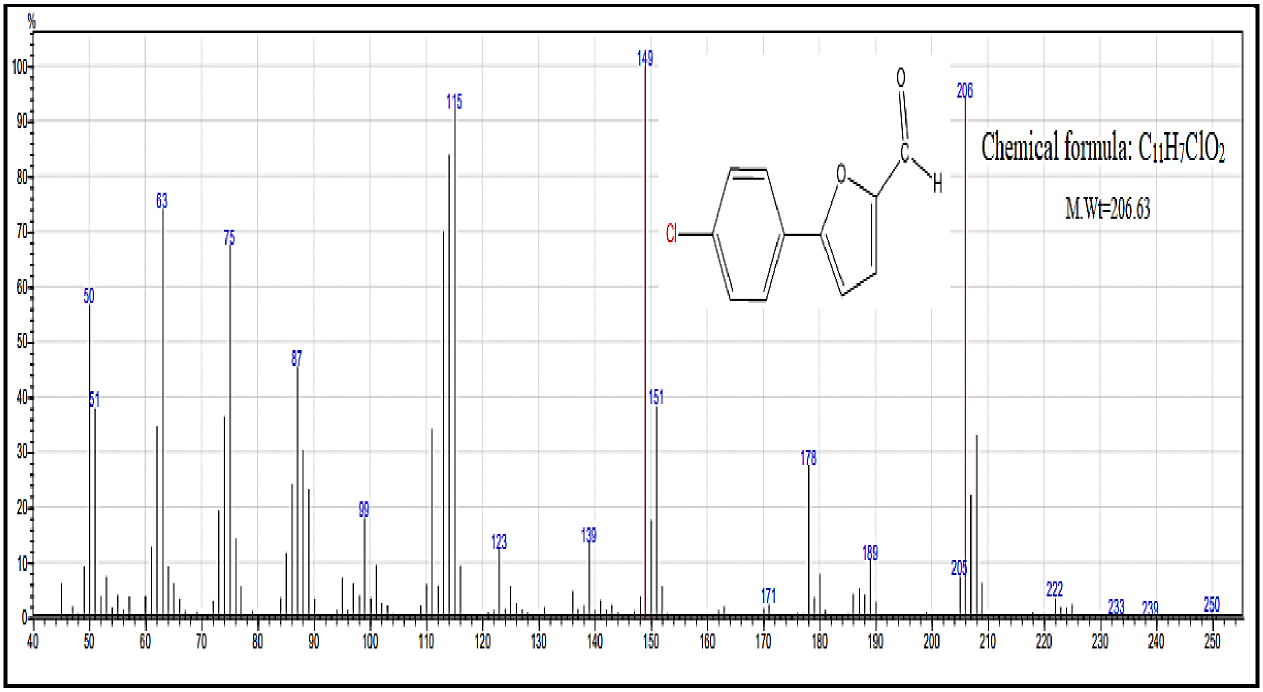

Mass Spectrum of compound 1a

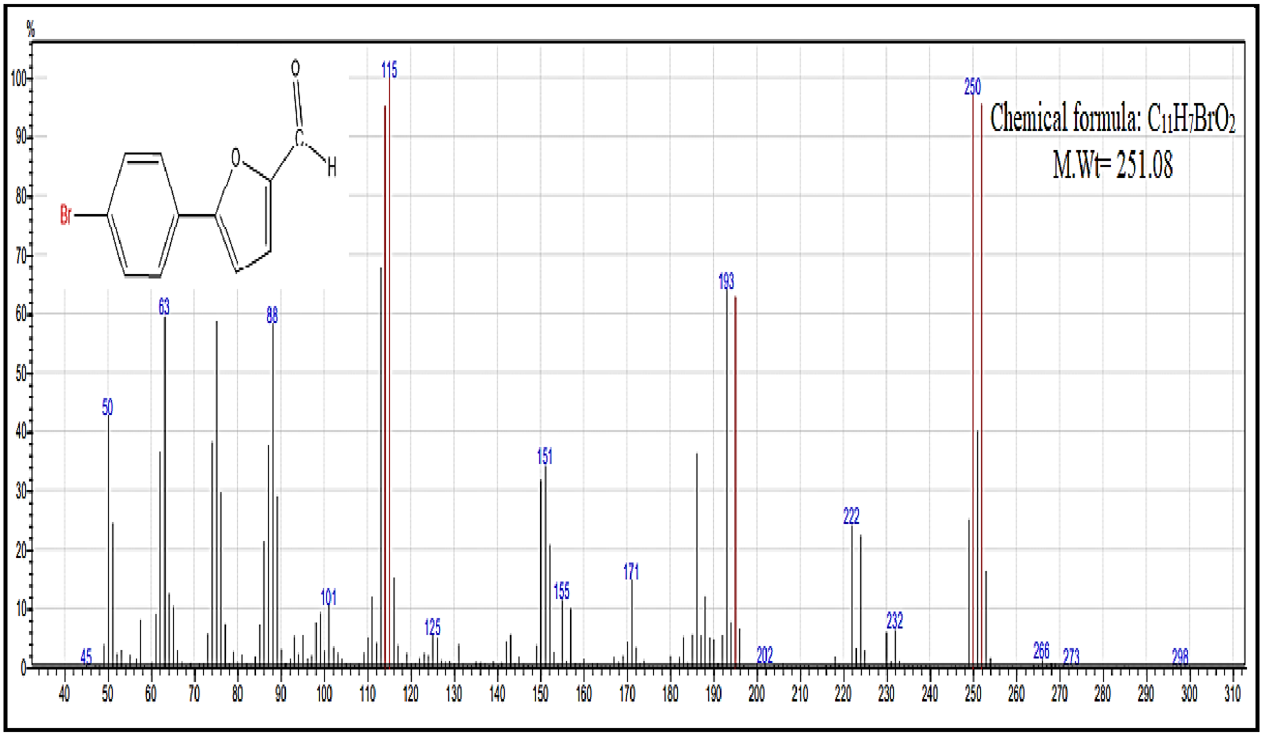

Mass Spectrum of compound 1b

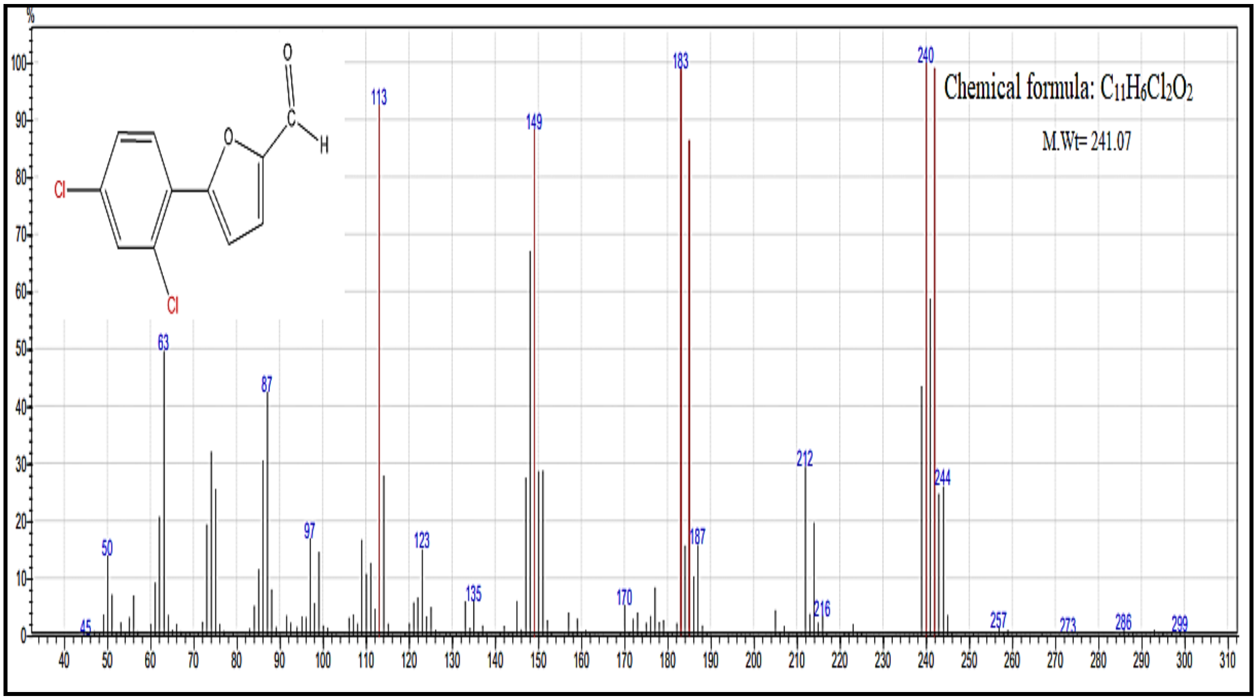

Mass Spectrum of compound 1c

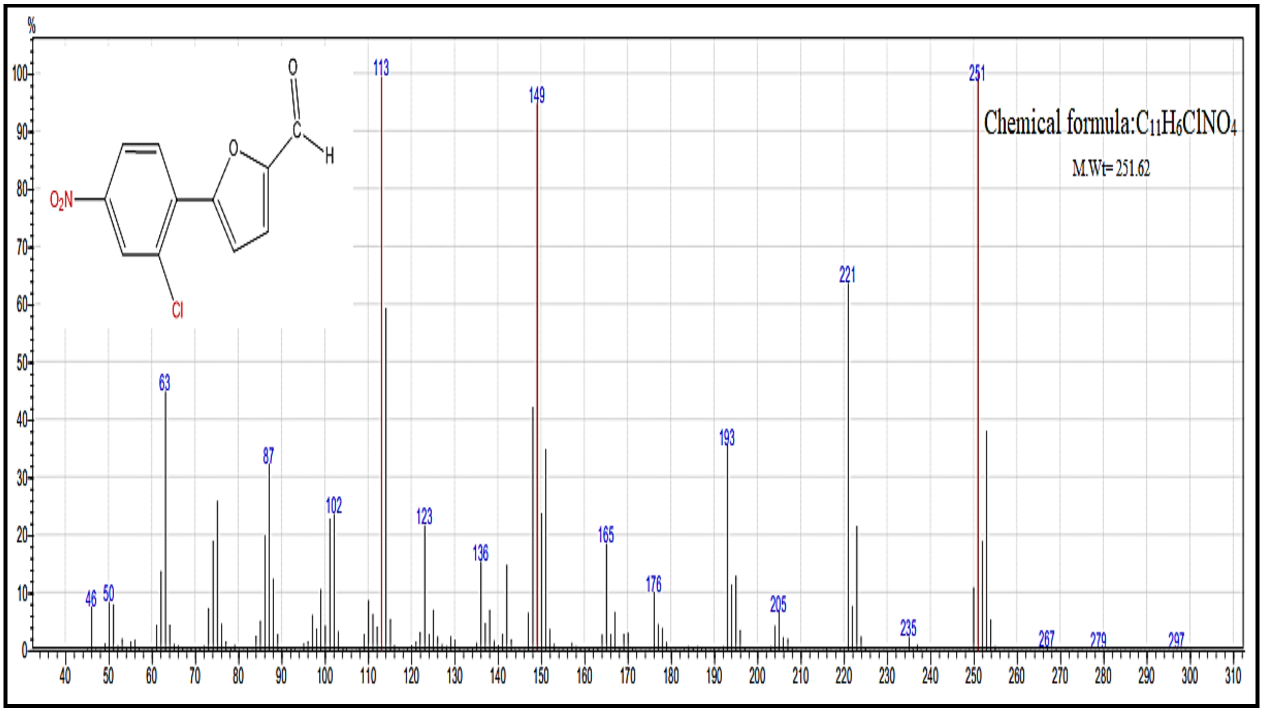

Mass Spectrum of compound 1d

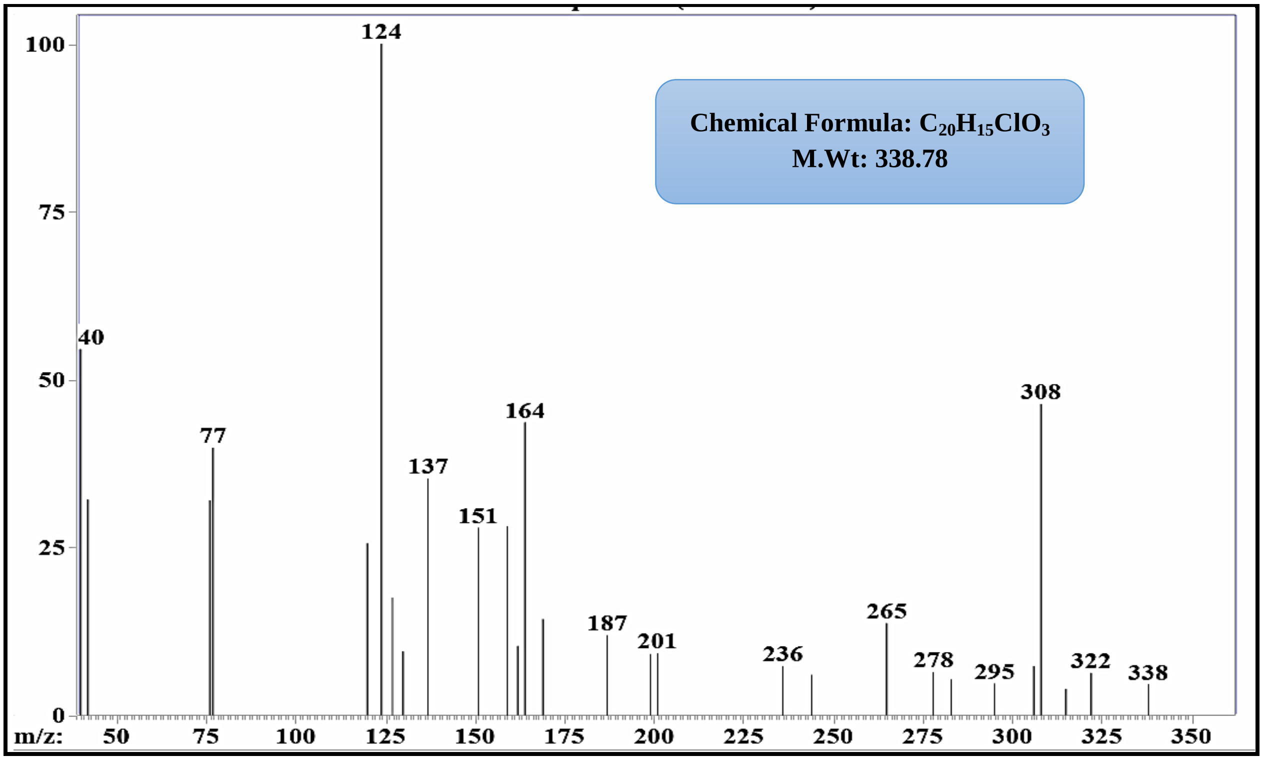

GC-Mass Spectrum of compound 2a

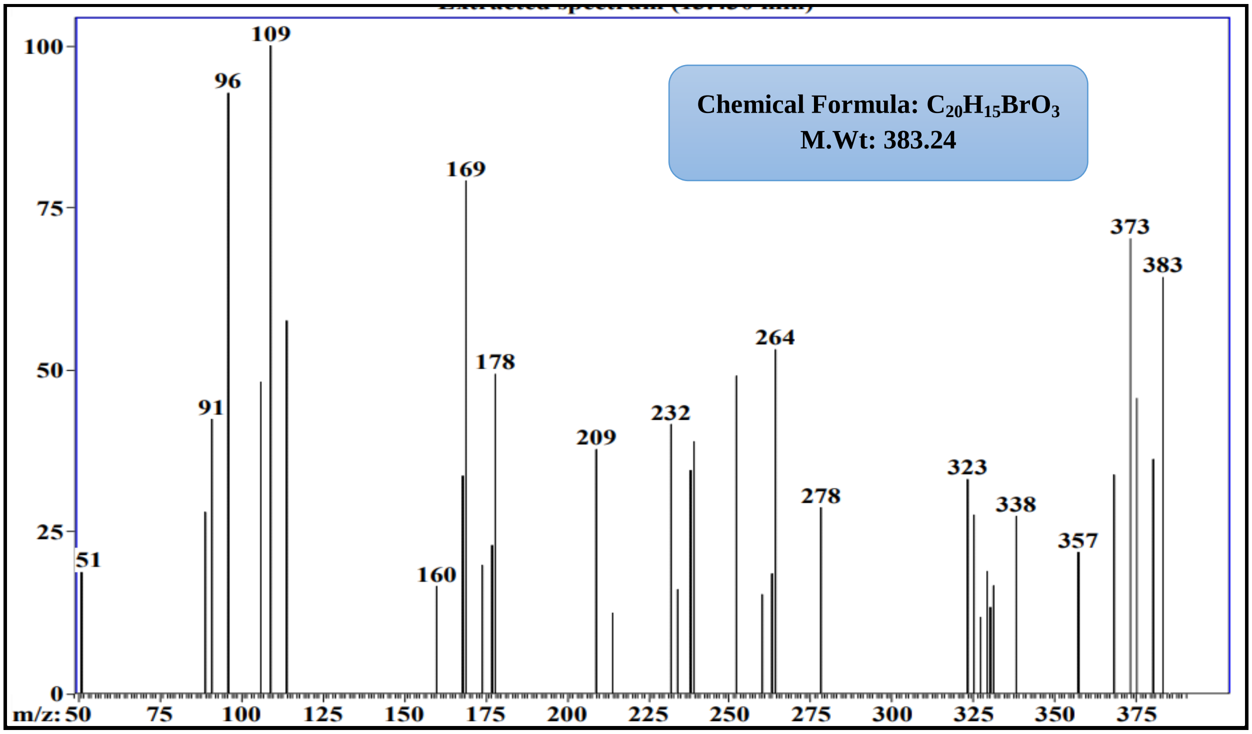

GC-Mass Spectrum of compound 2b

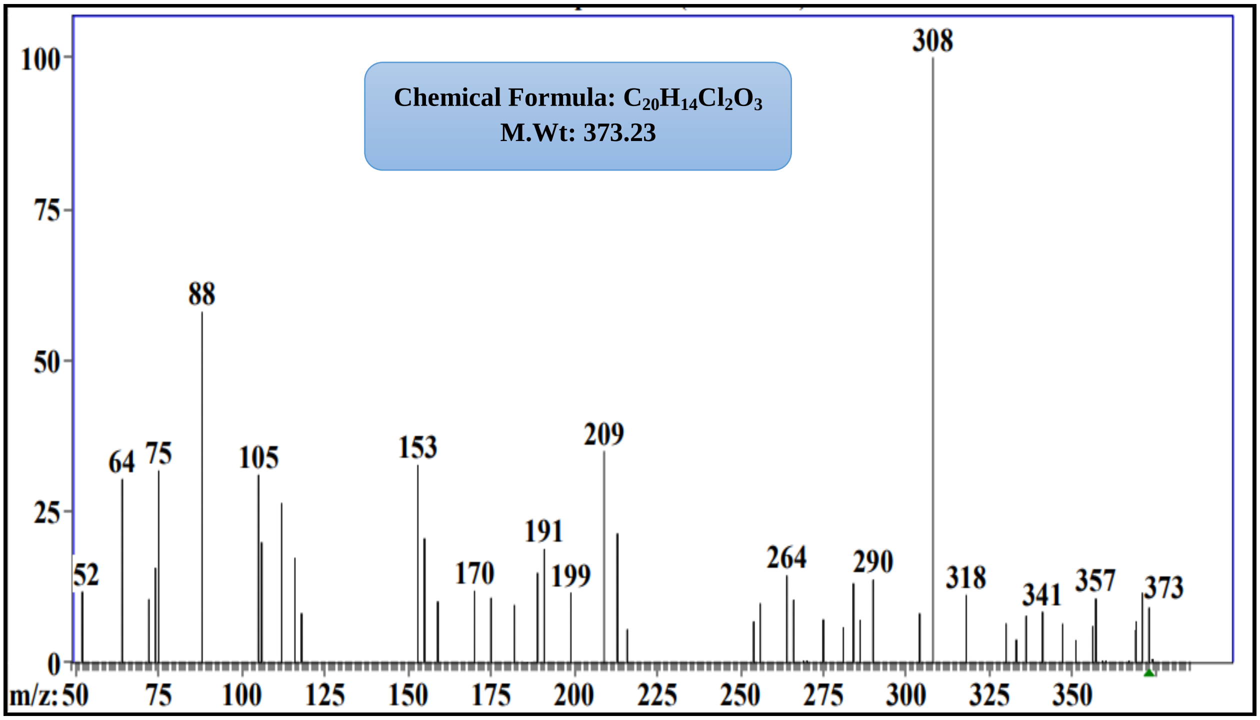

GC-Mass Spectrum of compound 2c

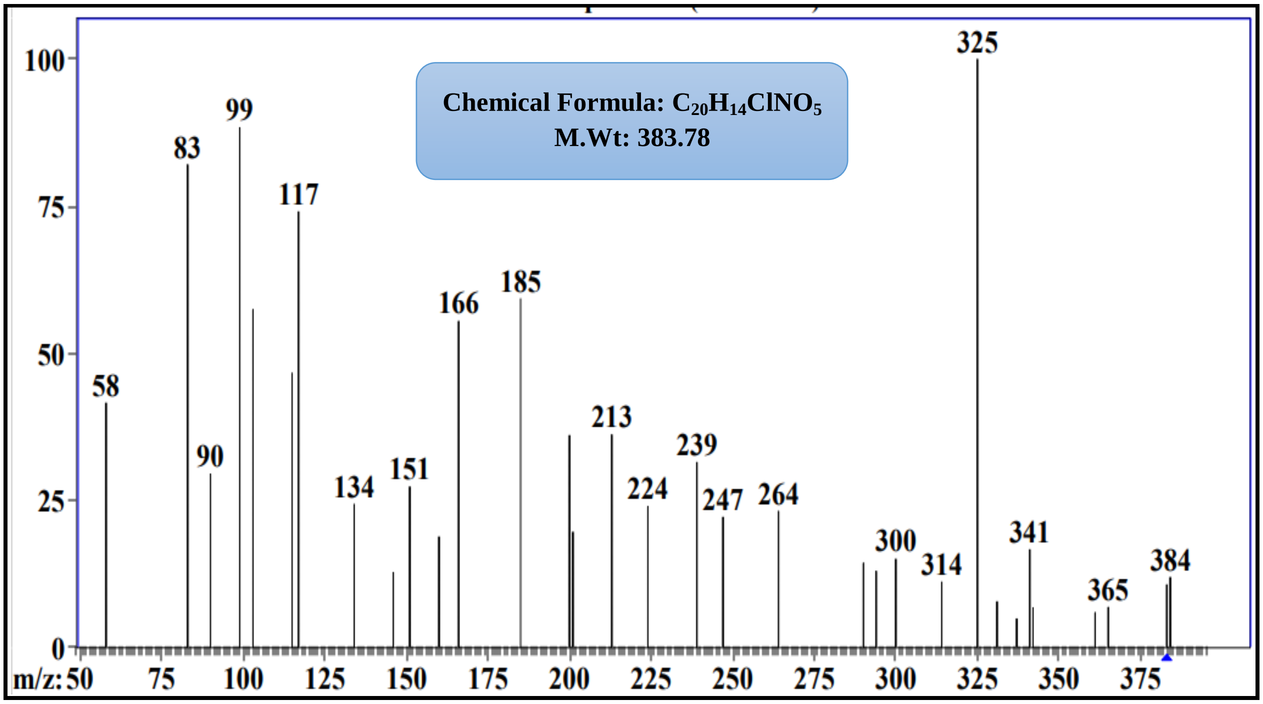

GC-Mass Spectrum of compound 2d

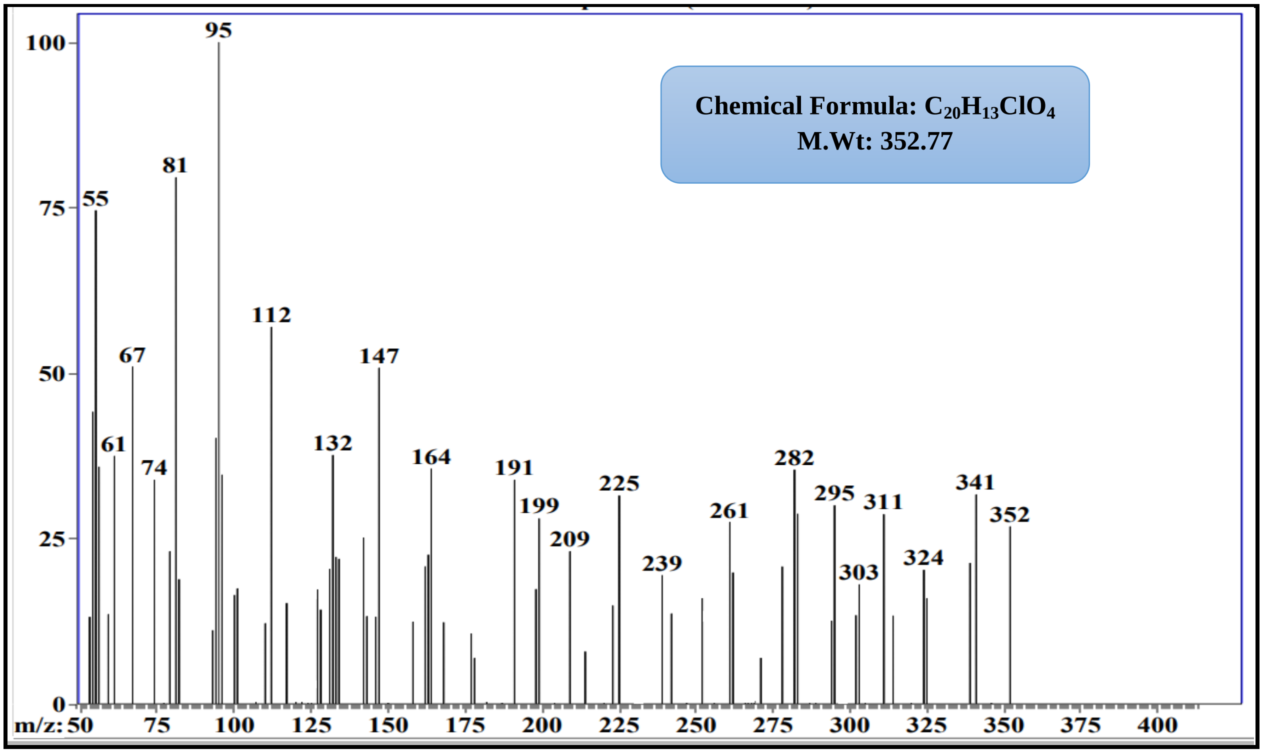

GC-Mass Spectrum of compound 2e

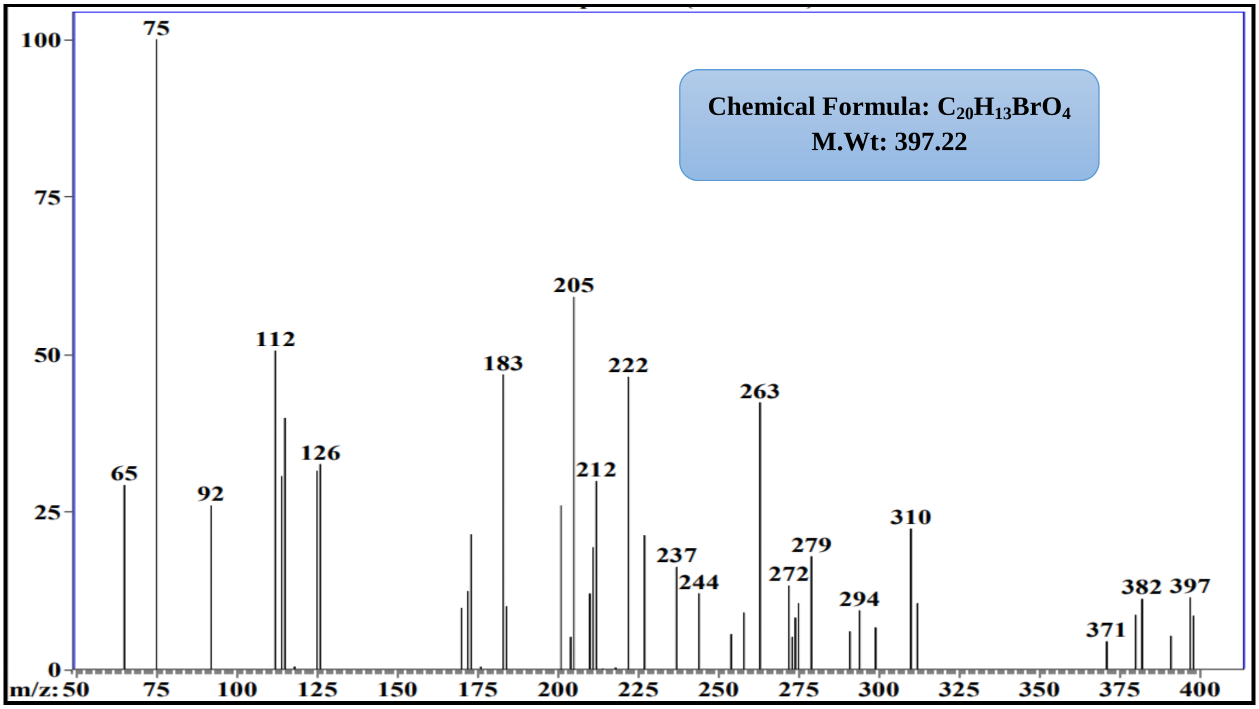

GC-Mass Spectrum of compound 2f

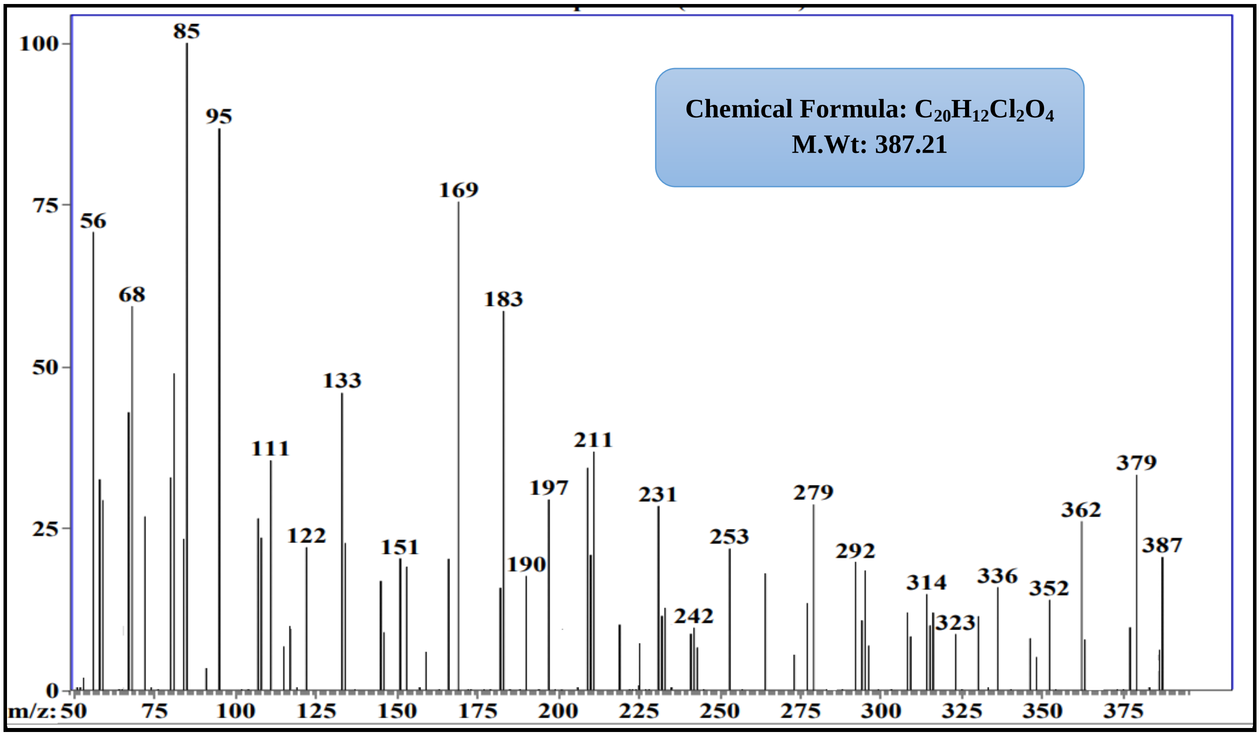

GC-Mass Spectrum of compound 2g

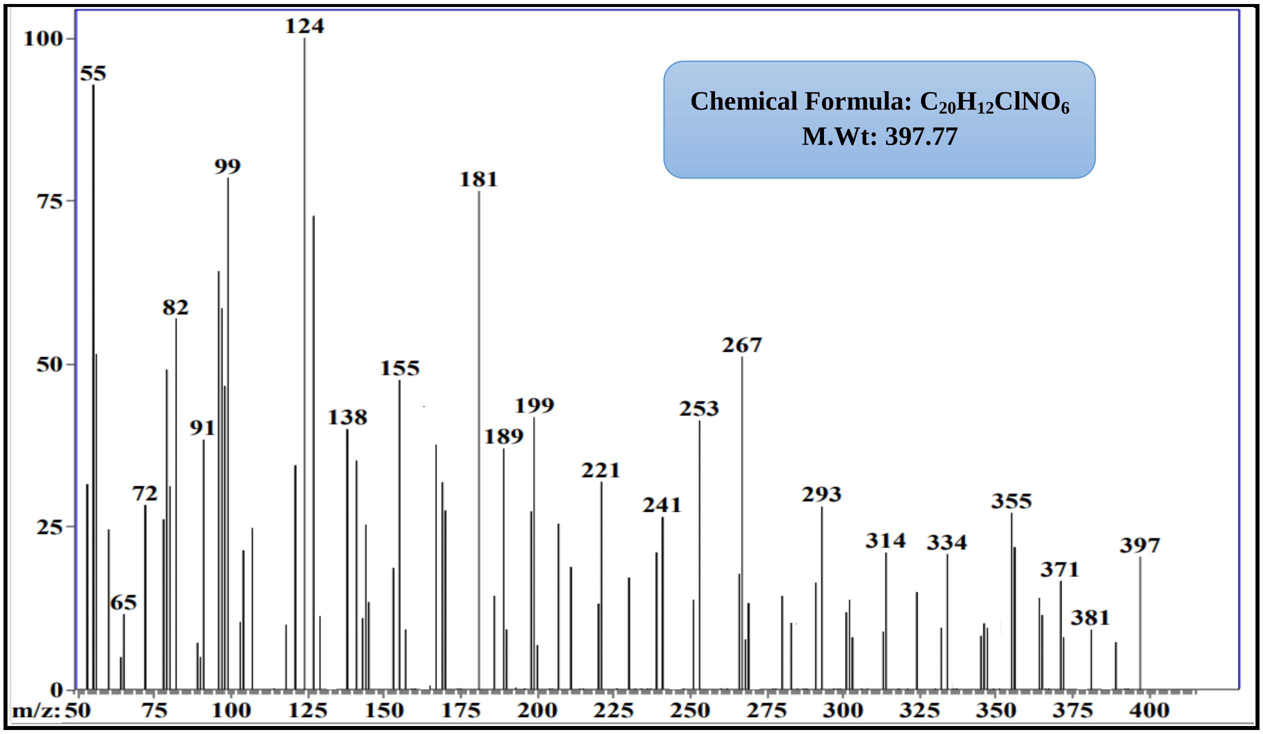

GC-Mass Spectrum of compound 2h

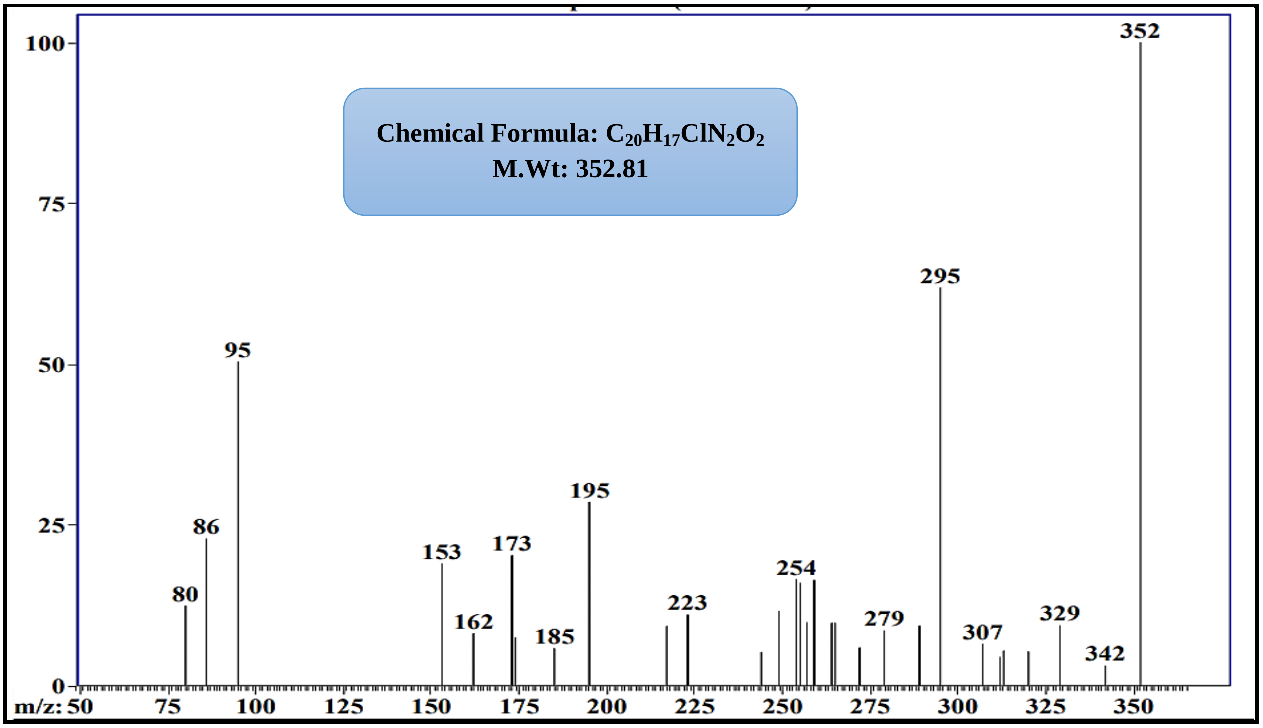

GC-Mass Spectrum of compound 3a

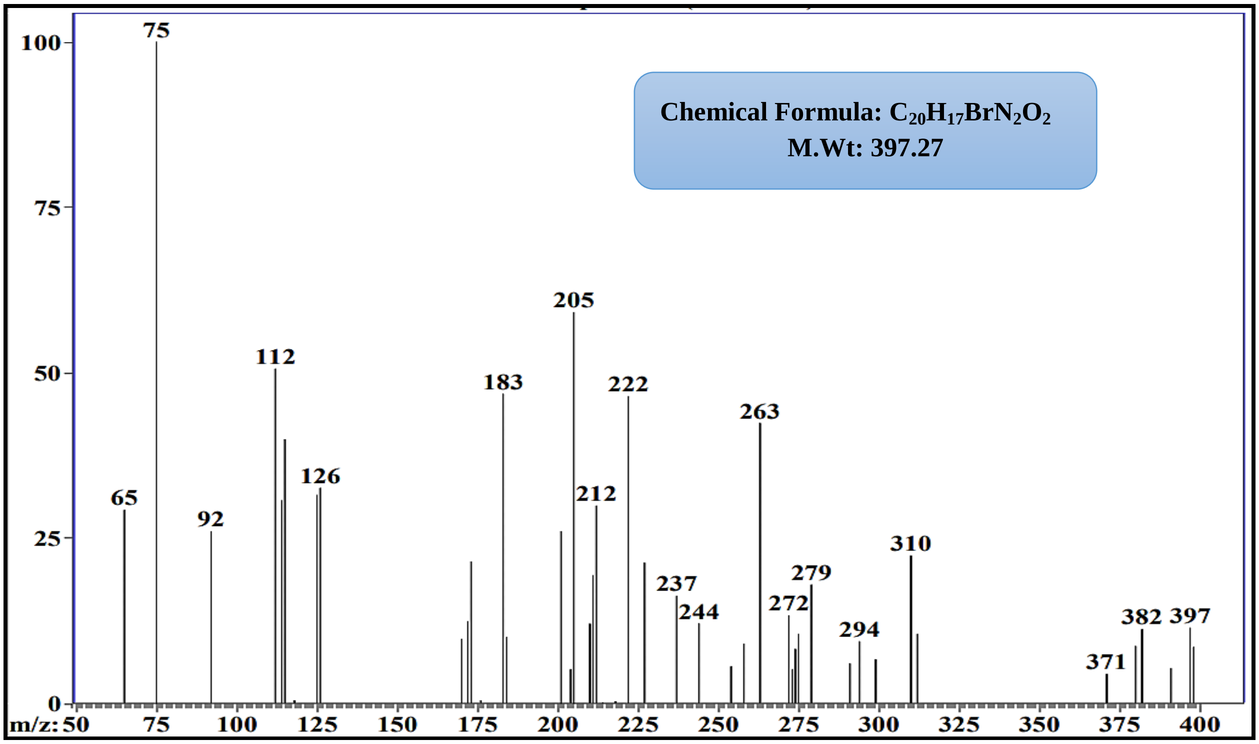

GC-Mass Spectrum of compound 3b

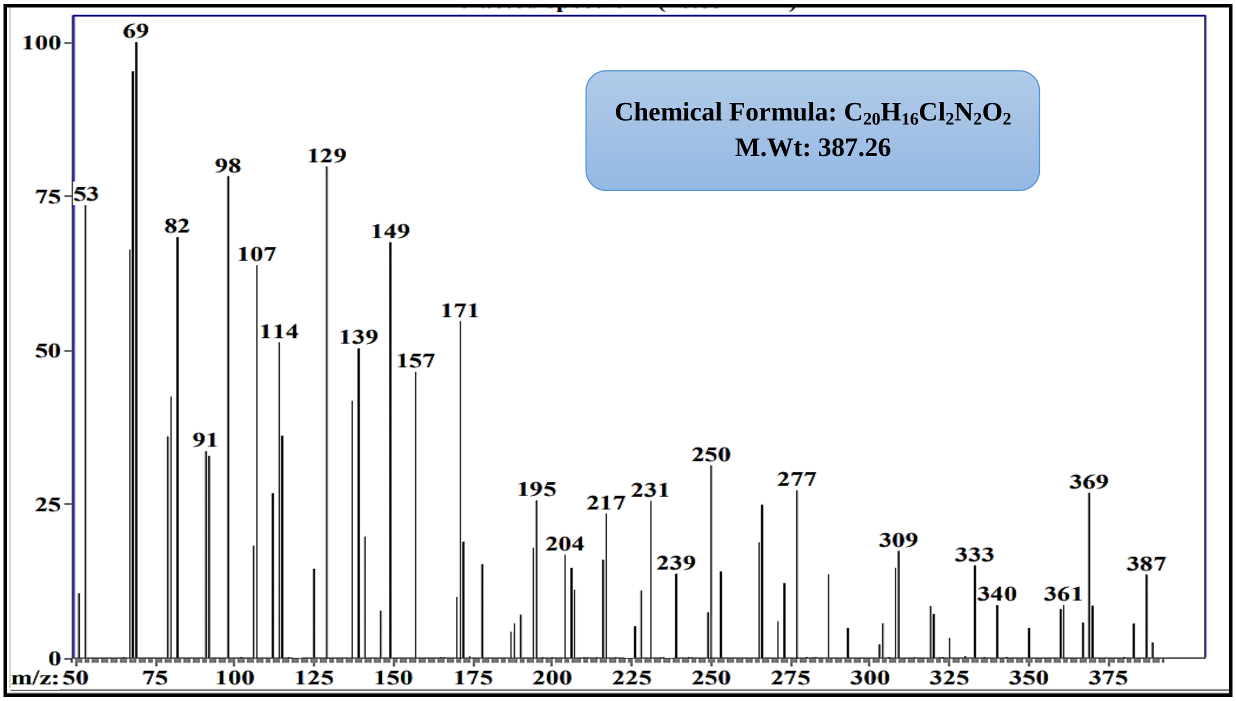

GC-Mass Spectrum of compound 3c

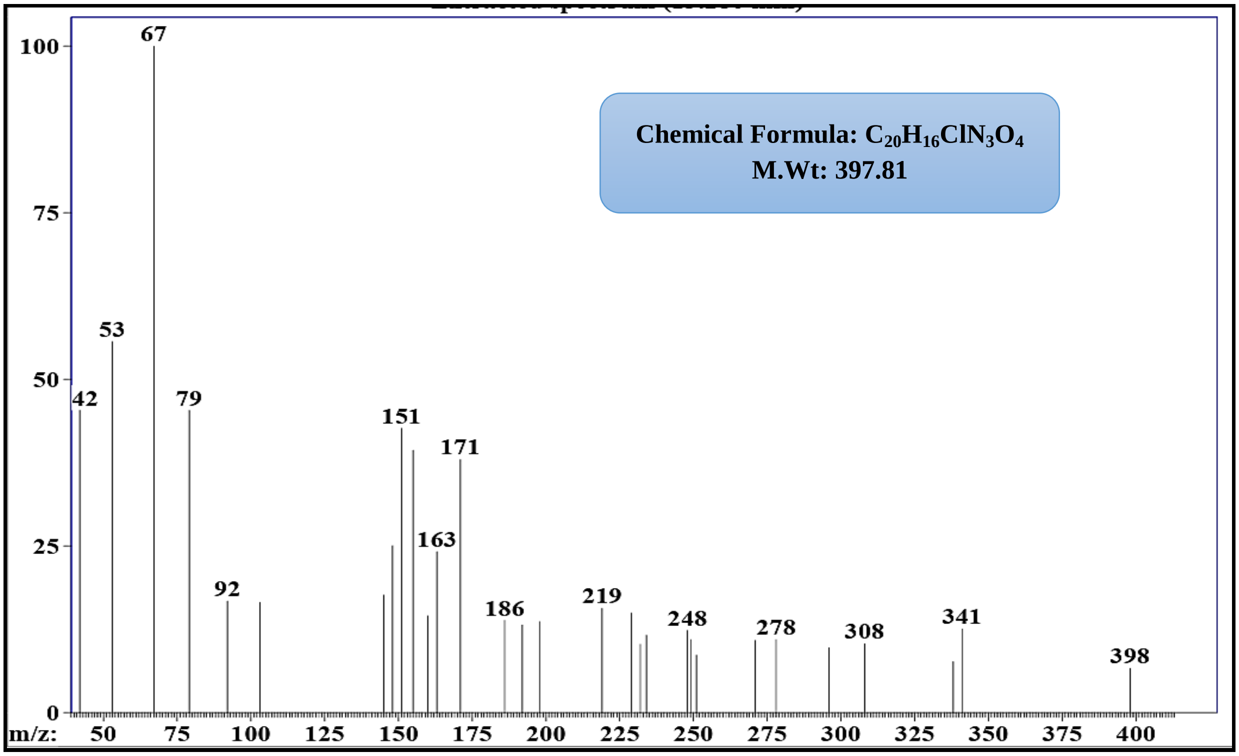

GC-Mass Spectrum of compound 3d

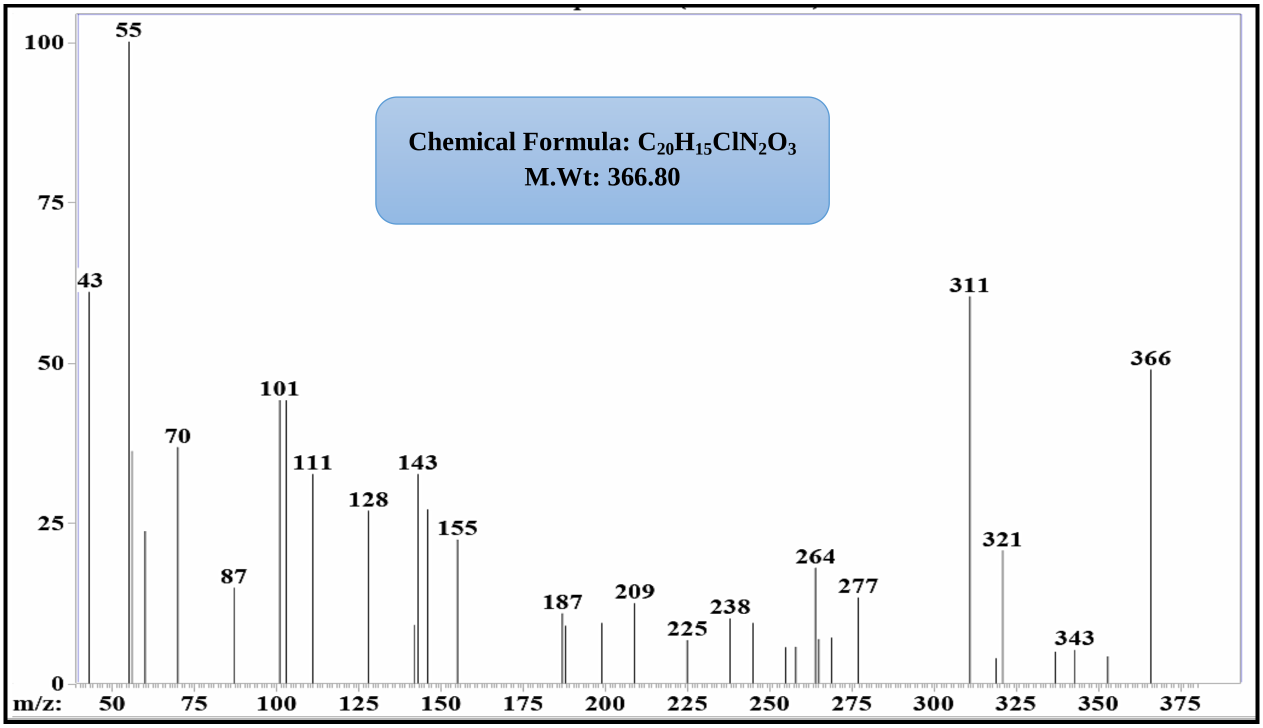

GC-Mass Spectrum of compound 3e

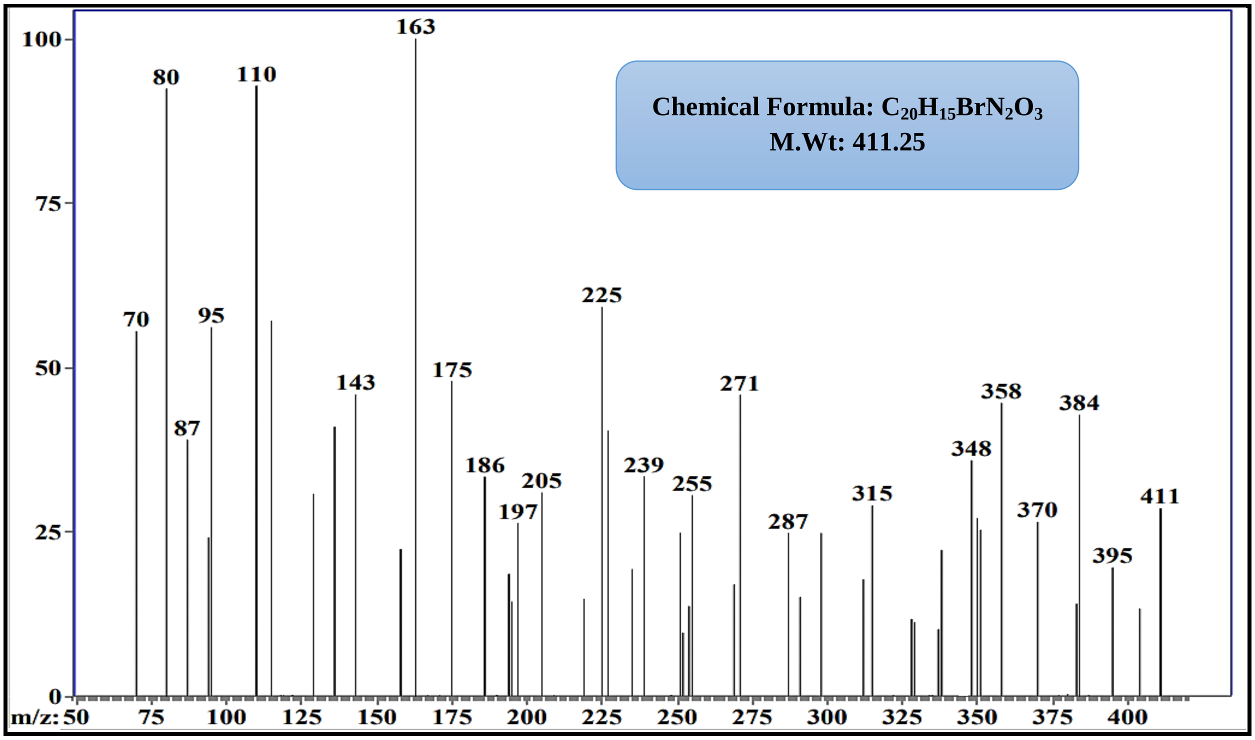

GC-Mass Spectrum of compound 3f

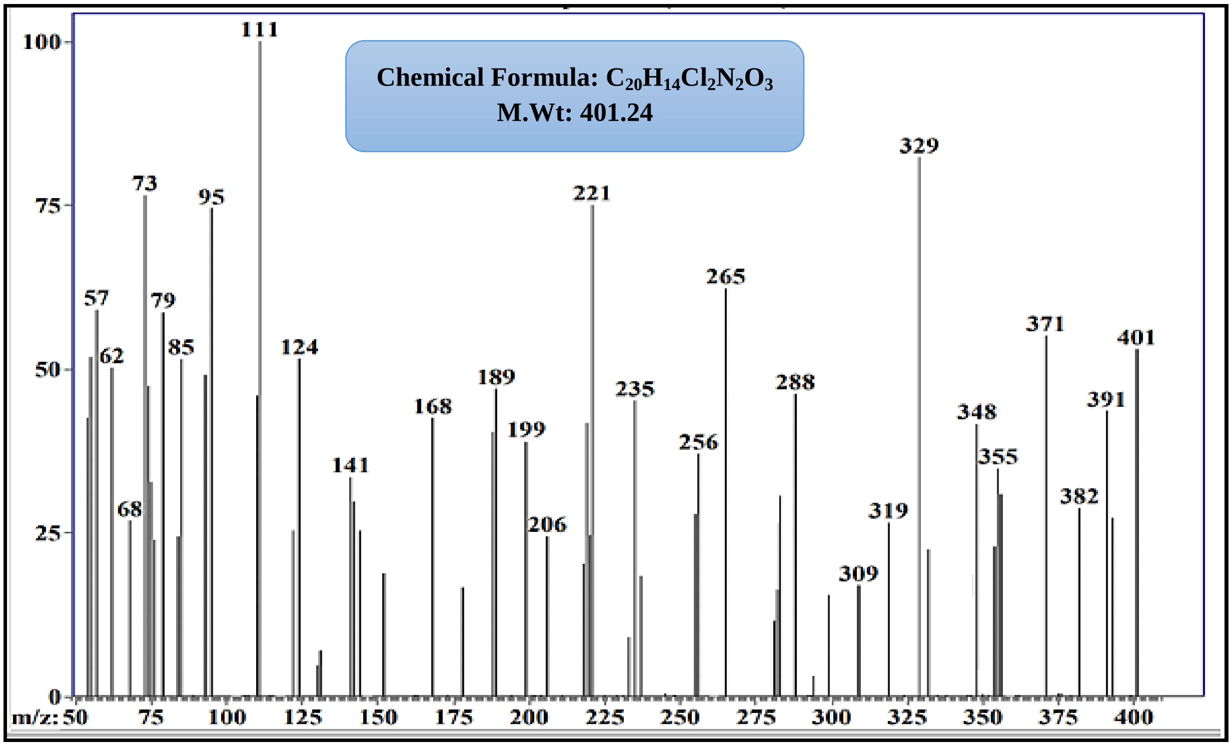

GC-Mass Spectrum of compound 3g

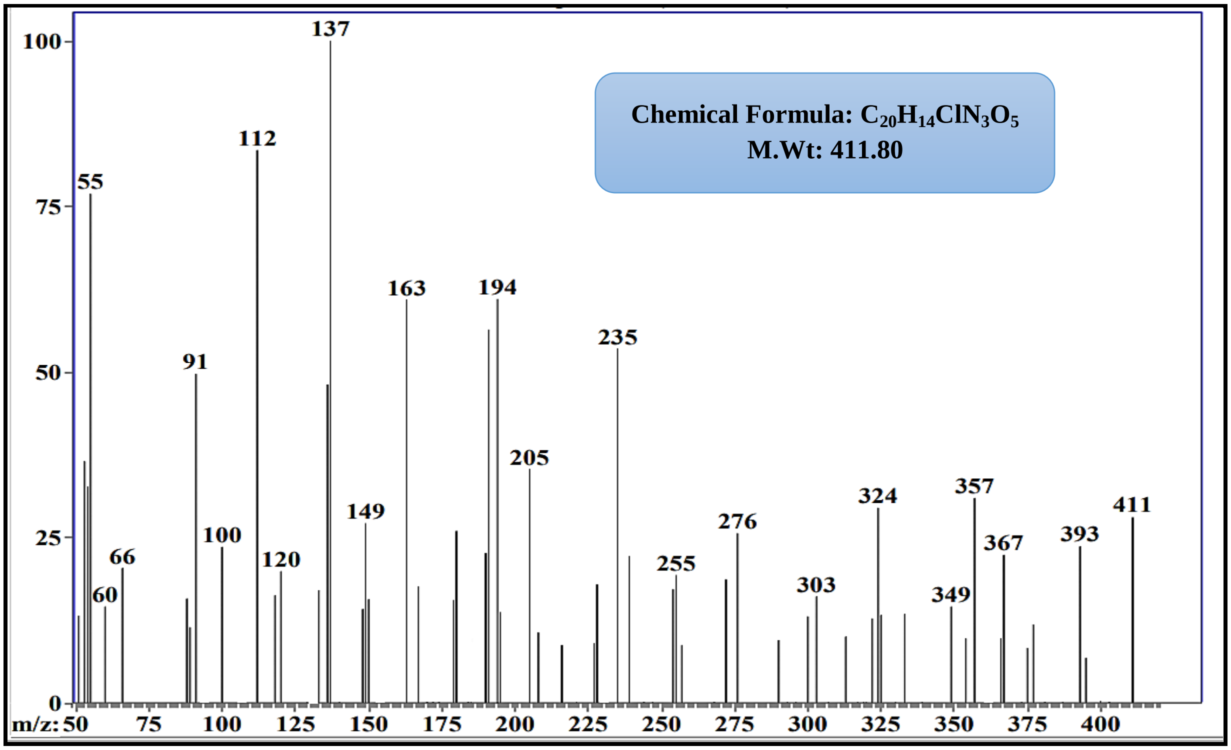

GC-Mass Spectrum of compound 3h

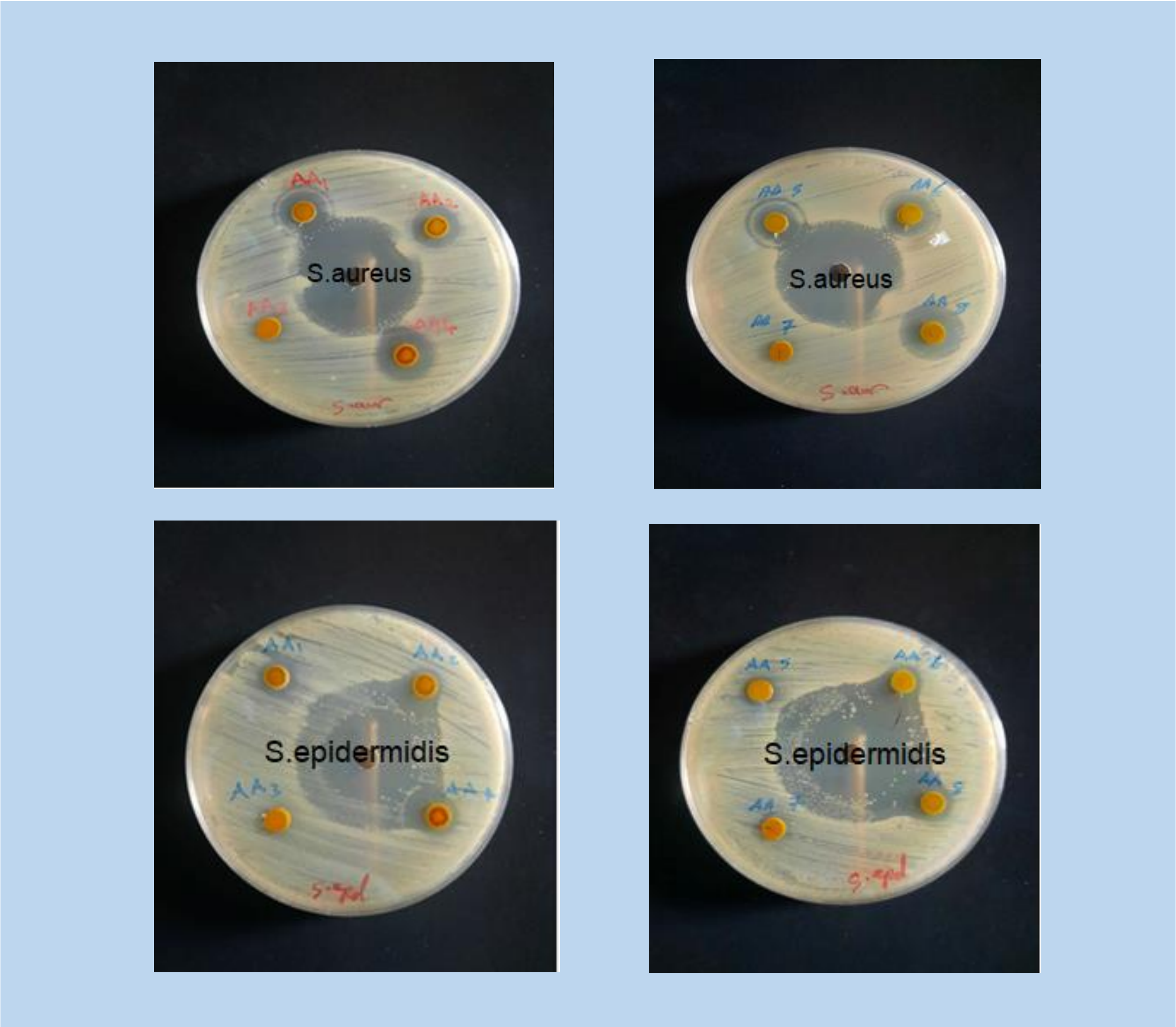

Inhibition zone of compounds 2a-h (AA1-AA8) against *Staphylococcus aureus* and *Staphylococcus epidermidis* (gram+ve).

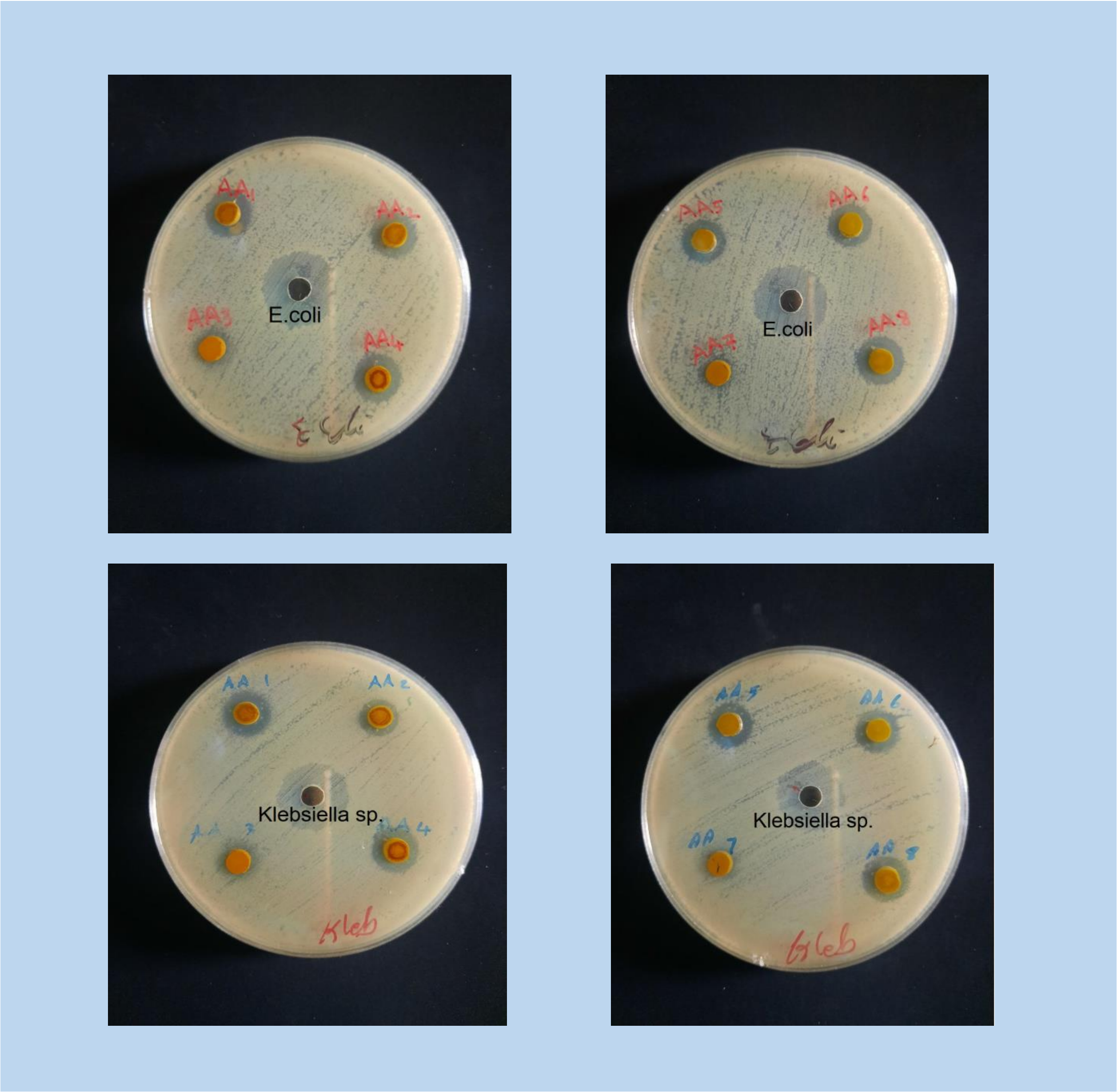

Inhibition zone of compounds 2a-h (AA1-AA8) against *Escherichia coli* and *klebsiella sp.* (gram-ve)

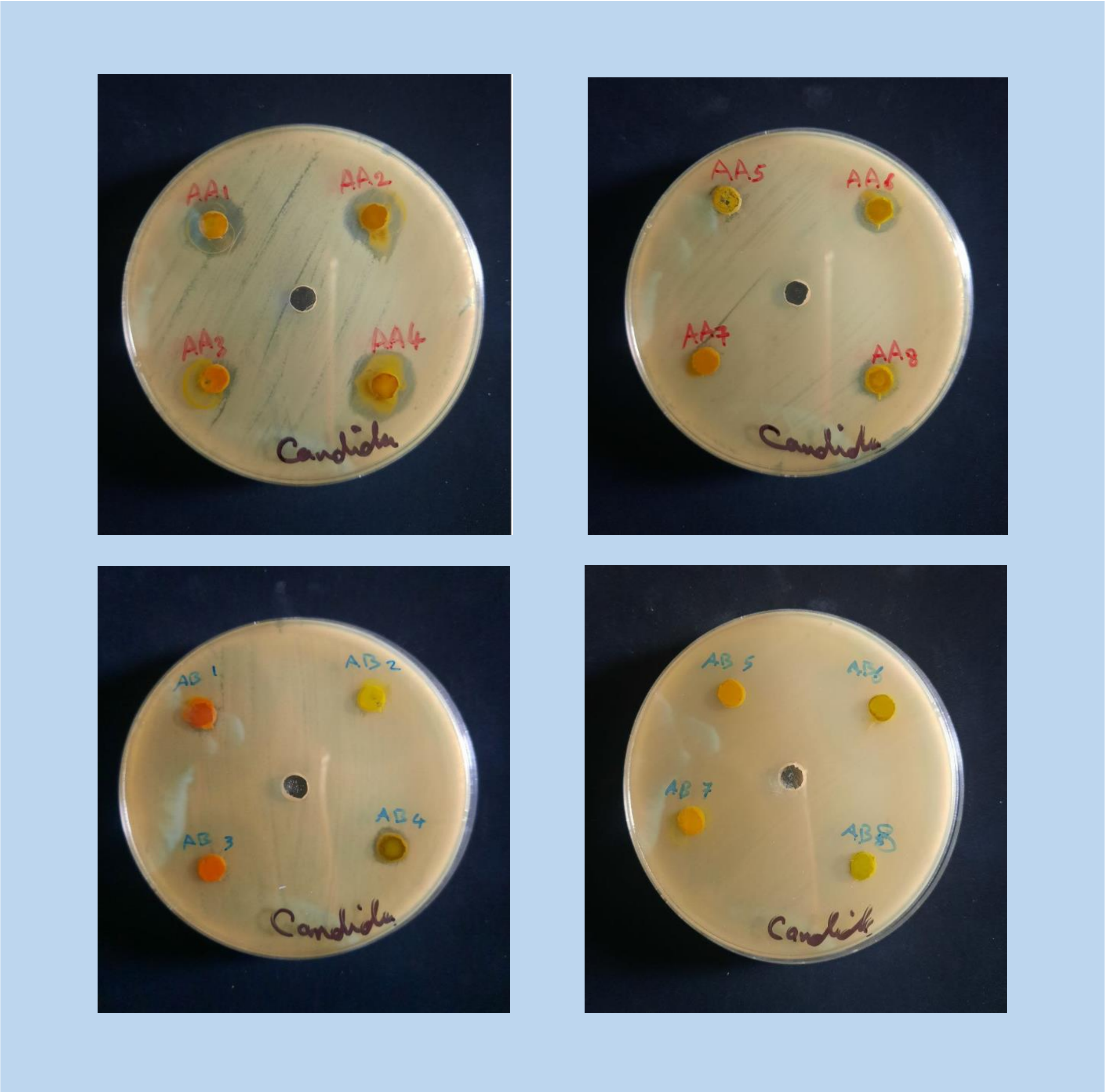

Inhibition zone of compounds 2a-h (AA1-AA8) and 3a-h (AB1-AB8) against *Candida albicans*

Docking parameters of compounds 2a, 2h and 3d

Compounds	Conformers	Binding Energy (Kcal mol-1)	Inhibition constant (µM)	Intermolecular energy (kcalmol^{-1})	H-bonds	Bonding
2a	1	-7.91	1.60	- 9.40	3	SER303:HG:LIG:O<br>SER401:HG:LIG:O<br>GLN348:HN:LIG:O
	2	-7.91	1.58	- 9.41	3	SER303:HG:LIG:O<br>SER401:HG:LIG:O<br>GLN348:HN:LIG:O
	3	-7.72	2.20	- 9.21	3	SER303:HG:LIG:O<br>SER401:HG:LIG:O<br>GLN348:HN:LIG:O
	4	-7.70	2.25	- 9.20	3	SER303:HG:LIG:O<br>SER401:HG:LIG:O<br>GLN348:HN:LIG:O
	5	-7.70	2.26	- 9.19	3	SER303:HG:LIG:O<br>SER401:HG:LIG:O<br>GLN348:HN:LIG:O
	6	-7.66	2.44	- 9.15	2	SER401:HG:LIG:O<br>GLN348:HN:LIG:O
	7	-7.55	2.91	- 9.04	3	SER303:HG:LIG:O<br>SER401:HG:LIG:O<br>GLN348:HN:LIG:O
	8	-7.53	3.01	- 9.02	3	SER303:HG:LIG:O<br>SER401:HG:LIG:O<br>GLN348:HN:LIG:O
	9	-7.37	3.94	- 8.87	3	THR352:HG1:LIG:O<br>SER401:HG:LIG:O<br>LYS603:HZ3:LIG:O
	10	-7.36	4.02	- 8.85	2	THR352:HG1:LIG:O<br>LYS603:HZ3:LIG:O

Compounds	Conformers	Binding Energy (Kcal mol-1)	Inhibition constant (μM)	Intermolecular energy (kcalmol^{-1})	H-bonds	Bonding
2h	1	-8.31	0.80 *10^{-3}	- 9.80	4	SER401:HG:LIG:O<br>ALA602:HN: LIG:O<br>SER401:HN:LIG:O<br>VAL607:HN: LIG:O
	2	-8.12	1.12	- 9.61	4	SER401:HG:LIG:O<br>ALA602:HN: LIG:O<br>SER401:HN:LIG:O<br>VAL607:HN: LIG:O
	3	-7.98	1.42	- 9.47	4	ASN600:HD22:LIG:O<br>ALA602:HN:LIG:O<br>SER401:HN:LIG:O<br>SER401:HG:LIG:O
	4	-7.69	2.30	- 9.18	2	ASN305:HD22:LIG:O<br>LYS603:HZ3:LIG:O
	5	-7.52	3.10	- 9.01	3	SER349:HN:LIG:O<br>GLN348:HN:LIG:O<br>SER401:HG:LIG:O
	6	-7.34	4.18	- 8.83	2	SER349:HN:LIG:O<br>SER401:HG:LIG:O
	7	-7.32	4.31	- 8.81	2	GLN348:HN:LIG:O<br>SER401:HG:LIG:O
	8	-7.08	6.45	- 8.57	2	SER401:HG:LIG:O<br>SER349:HN:LIG:O
	9	-6.89	8.83	- 8.39	2	THR352:HG1:LIG:O<br>LYS603:HZ3:LIG:O
	10	-6.65	13.44	- 8.14	2	GLN348:HN:LIG:O<br>SER349:HN:LIG:O

Compounds	Conformers	Binding Energy (Kcal mol-1)	Inhibition constant (μM)	Intermolecular energy ($kcalmol^{-1}$)	H-bonds	Bonding
3d	1	-7.45	2.96	- 9.03	1	THR352:HG1: LIG:O
	2	-7.51	3.11	- 9.00	1	THR352:HG1: LIG:O
	3	-7.49	3.23	- 8.98	1	THR352:HG1: LIG:O
	4	-7.47	3.36	- 8.96	2	GLY301:HN: LIG:O<br>GLN348:HN:LIG:O
	5	-7.47	3.33	- 8.96	1	THR352:HG1: LIG:O
	6	-7.37	3.93	- 8.87	3	GLN348:HN:LIG:O<br>THR352:HG1:LIG:O<br>SER303:HG:LIG:O
	7	-7.26	4.79	- 8.75	4	THR352:HG1:LIG:O<br>SER303:HG:LIG:O<br>GLN348:HN:LIG:O<br>GLY301:HN: LIG:O
	8	-7.21	5.22	- 8.70	2	CYS300:HN: LIG:O<br>THR352:HG1:LIG:O
	9	-6.82	10.03	- 8.31	2	GLN348:HN:LIG:O<br>SER349:HN:LIG:O
	10	-6.77	10.85	- 8.26	3	GLN348:HN:LIG:O<br>SER349:HN:LIG:O<br>ASP354:HN: LIG:O
